# Potassium *tert-*Butoxide Promoted Proton Transfer-Electron Transfer in Aerobic C(sp^3^)–H Hydroxylations

**DOI:** 10.1021/acs.inorgchem.6c00906

**Published:** 2026-04-28

**Authors:** Changming Li, Hongdan Zhu, Qian Peng

**Affiliations:** † State Key Laboratory of Elemento-Organic Chemistry, Tianjin Key Laboratory of Biosensing and Molecular Recognition, College of Chemistry, Frontiers Science Center for New Organic Matter, 12538 Nankai University, Tianjin 300071, China; ‡ Haihe Laboratory of Sustainable Chemical Transformations, Tianjin 300192, China

## Abstract

Aerobic C­(sp^3^)–H hydroxylation is a crucial chemical process, where alkali-metal reagents act as key mediators that profoundly influence the reaction mechanism. However, the mechanistic understanding of such a reaction remains limited, probably due to the complex electronic structure of the reaction involving the O_2_ and the formed clusters of alkali metal reagent aggregations. Herein, we propose a new Alkali Metal Reagent Promoted Proton Transfer-Electron Transfer (A-PTET) mechanism in aerobic C­(sp^3^)–H hydroxylations by density functional theory (DFT) calculations to understand the different radical detection outcomes depending on the amount of KO^*t*^Bu. With catalytic KO^*t*^Bu, the reaction proceeds through the carbanion pathway, and thus, radical species are undetected experimentally. In contrast, when 3 equiv of KO^*t*^Bu are employed, the positive electrostatic field generated by K^+^ cations stabilizes radical intermediates that induce the single electron transfer in the A-PTET mechanism, leading to the experimentally observed radical species. This study establishes a theoretical foundation for the development of aerobic reactions, and the A-PTET mechanism may extend to other reactions based on new insights into alkali-metal reagents.

## Graphical abstract

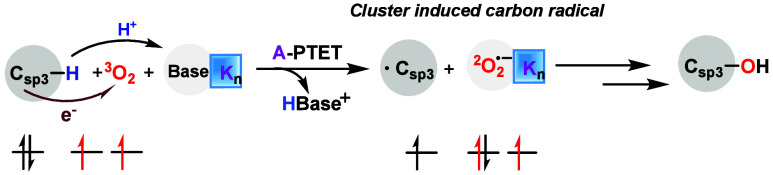

## 1 Introduction

The aerobic C­(sp^3^)–H oxidations represent a highly attractive yet challenging area in synthetic chemistry.^1–4^ This strategy enables the direct conversion of ubiquitous inert C­(sp^3^)–H bonds into various oxygen-containing functional groups (e.g., alcohols, ketones, aldehydes, carboxylic acids), thereby offering a direct and green pathway for the synthesis of high-value fine chemicals.^5–8^ The C­(sp^3^)–H bond dissociation energy is relatively high (90–100 kcal/mol), rendering such bonds rather inert. Moreover, their direct reaction with triplet oxygen (^3^O_2_) is spin-forbidden.^9–12^ As a result, aerobic C­(sp^3^)–H oxidations are generally difficult to achieve. The cleavage of the C­(sp^3^)–H bond generates the carbanion or carbon radical intermediate, which can subsequently react with oxygen to form the final product.^1,13–15^ The carbanion mechanism involves the deprotonation of the C­(sp^3^)–H bond by a strong base, allowing nucleophilic attack on the ^3^O_2_ by the resulting *p*-orbital of carbanion.^16–18^ The carbon radical mechanism is usually achieved through the addition of transition metals,^19^ organic radicals,^20^ or photocatalysts^21^ that facilitate hydrogen atom transfer (HAT) of the C­(sp^3^)–H bond. However, aerobic C­(sp^3^)–H oxidation with alkali-metal reagents via a carbon radical mechanism rather than a carbanion mechanism remains scarce.

The aerobic C­(sp^3^)–H hydroxylation facilitated by alkali-metal reagents represents an important field.^22^ The groups led by Jiao,^23^ Gnanaprakasam,^24^ and Li^25^ have independently reported the aerobic oxidations of carbonyl compounds by different bases, such as Cs_2_CO_3_, KO^*t*^Bu, and an organic base, respectively. In these experiments, radical species cannot be observed through TEMPO trapping. In addition, their reaction mechanism relies on a carbanion intermediate via alkali metal assisting the triplet-to-singlet transition.^26^ Alternatively, for the 2-benzylpyridine substrates, Li group utilized 3 equiv of KO^*t*^Bu to achieve the hydroxylation reaction at 30 °C (**II**, see Scheme 1b).^27^ In this reaction, carbon radical species can be detected by EPR and control experiments. Interestingly, another Li group employed catalytic amounts of KO^*t*^Bu (0.1 equiv) to achieve the similar reaction (**I**, see Scheme 1b),^28^ but the control experiment did not detect the radical signal. With both of these reactions, dimethylsulfoxide (DMSO) was used as the solvent. Previously reported DMSO/KO^*t*^Bu system indicate the dimsyl anion, deprotonated from DMSO, acts as an electron donor to generate radical species.^29–33^ However, the mechanism for radical generation in aerobic C­(sp^3^)–H hydroxylations under the DMSO/KO^*t*^Bu system remains unclear. These different radical properties seem to be dependent on the amount of KO^*t*^Bu that prompted us to investigate the detailed reaction mechanism.

**Scheme 1:**
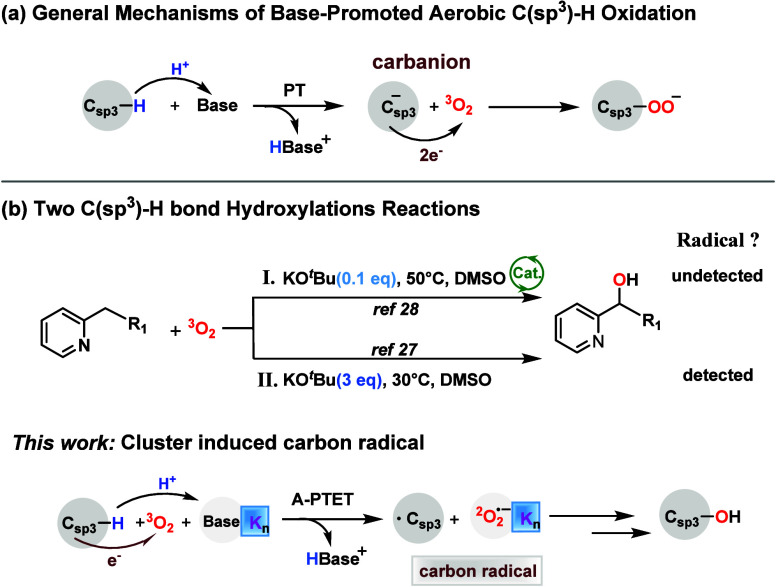
(a) General Mechanisms of Base-Promoted Aerobic C­(sp^3^)–H Oxidation. (b) Two C­(sp^3^)–H Hydroxylation Reactions^a^

a “This work” represents the mechanism of A-PTET (Alkali Metal Reagent Promoted Proton Transfer-Electron Transfer).

Reaction mechanisms of two aerobic C­(sp^3^)–H hydroxylations have been investigated by density functional theory (DFT) calculations (Scheme 1b). Key questions have to be addressed: (1) the generation mechanism of radical species and (2) the effect of alkali-metal reagents in different equivalents. Herein, we propose an “Alkali Metal Reagent Promoted Proton Transfer-Electron Transfer” (A-PTET) mechanism. This mechanism reveals that the cationic positive electric field of the alkali-metal cluster can induce carbon radical formation, which is distinct from the traditional carbanion mechanism.

## 2 Computational Details

The DFT calculations were performed using the Gaussian 16 software package.^34^ Geometry optimizations of intermediates and transition states (TS) were conducted in the gas phase at the CAM-B3LYP^35^ level of theory, including Grimme’s D3 dispersion corrections. A mixed basis set was employed, with 6–31G­(d,p)^37^ for H, C, O, N, S, and P atoms, and SDD^38^ for K. The B3LYP^39^-D3,^36^ ωB97XD,^40^ and PBE0-D3 ^36^ functional have been tested for benchmark calculations, and the similar results suggest that the CAM-B3LYP-D3 functional is reliable (Table S1). Triplet and singlet biradical species were optimized using unrestricted CAM-B3LYP (UCAM-B3LYP) in conjunction with broken-symmetry methodology, and intrinsic reaction coordinate (IRC)^42^ calculations were also performed. Vibrational frequency analyses at the same level confirmed the identity of each stationary point: minima were identified by having no imaginary frequencies and transition states by exactly one imaginary frequency. These calculations also provided zero-point energy (ZPE) and thermal corrections at 298 K. For key transition states and intermediates, structural optimizations in DMSO solvent under CPCM mode were further evaluated by the same functional ions with the basis set SDD for K, and 6–31G­(d,p) for other atoms (Table S2). Single-point energies of all calculated structures were further calculated using the UCAM-B3LYP-D3 and a mixed basis set comprising SDD for K, and 6–311++G­(d,p) for all other atoms under the SMD solvent model in implicit DMSO.^43^ More single-point energy calculations with CAM-B3LYP-D3/ANO-RCC-VDZP using Beijing Density Functional (BDF) software^44–47^ were performed for key transition states and intermediates in Figure 1 (see details in Table S1), which validates the rationality of computational methods employed in this work. In addition, conformational search was conducted by GFN0–xTB for key intermediates (**3-*n*K,*n*= 1–4**) to ensure the stable conformation (Figure S1). Minimal energy crossing points (MECP) between states of different spin multiplicity were located via the sobMECP program.^48,49^ Molecular illustrations were generated with CYLView,^50^ and Natural Bond Orbital (NBO) analysis was carried out using the NBO6 program.^51,52^ The activation barriers for the SET reactions (Δ*G*_SET_^⧧^) was estimated based on Marcus theory^53^ (the computational method is described in the Supporting Information (SI), Figure S2).

## 3 Results and Discussion

In Scheme 2, the possible reaction mechanisms are illustrated. The substrate **A** reacts with O_2_ via two distinct pathways to form peroxide **D**: (1) the carbanion pathway (direct oxygenation), where **A** is deprotonated by KO^*t*^Bu, and the resulting carbanion **B** undergoes direct oxygenation via intersystem crossing (ISC) to form **D**;^16–18^ and (2) the carbon radical pathway (PTET), where after the deprotonation (proton transfer PT), the formed carbanion immediately transfers a single electron to O_2_ (SET), generating carbon radical **C** and a superoxide radical (O_2_^• –^).^33^ Subsequently, radical coupling occurs to form **D**. The peroxide **D** then undergoes a redox reaction and protonation to yield the final hydroxylated product **F**. Based on the reaction **I** and **II** of Scheme 1b,^27,28^ DFT mechanistic studies of aerobic C­(sp^3^)–H hydroxylations were carried out using 2-benzylpyridine substrate to understand the KO^*t*^Bu cluster effect from K^+^ free to cluster models.

**Scheme 2:**
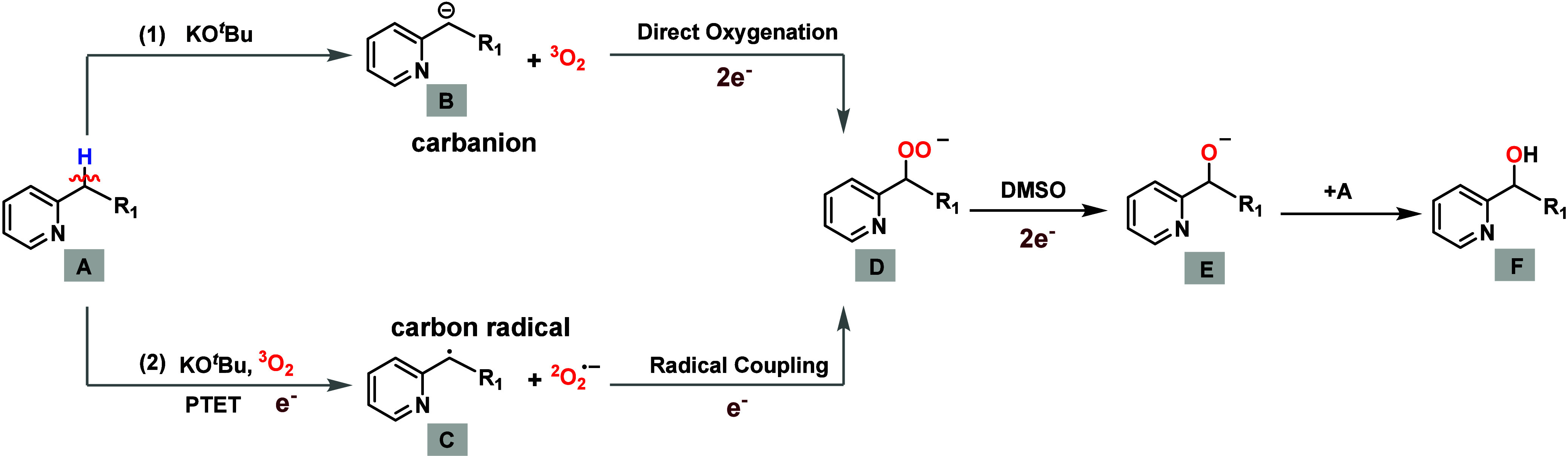
Possible Reaction Mechanisms of Hydroxylation Reactions

### (a) Carbanion Mechanism: K^+^ Free Model

In reaction **I**, KO^*t*^Bu was used at low loading (0.1 equiv). The solvent DMSO would coordinate with K^+^, effectively promoting the formation of separated ion pairs.^54–57^ Therefore, the computational model was built using anionic *tert*-butoxide. As shown in Figure 1, this hydroxylation proceeds primarily via a carbanion mechanism. 2-Benzylpyridine **1** undergoes the deprotonation via **TS1** (Δ*G* ^⧧^ = 11.4 kcal/mol) to form the carbanion intermediate **2**, which interacts with O_2_ to form intermediate ^3^ **3–0K** (1.85 spin population on the O_2_). This favorable potential energy curves (PECs) process through a key minimum energy crossing point (**MECP-0K**) between the triplet and singlet states, irreversibly yields peroxide **4** with an electronic energy barrier of 12.7 kcal/mol. For comparison, the carbon radical pathway (2) involves a SET process (Table S3), which has a higher barrier of 14.0 kcal/mol based on Marcus theory. The formed biradical intermediate (2^•^ + O_2_^• –^) is thermodynamically less stable than ^**3**^ **3–0K** by +3.8 kcal/mol (green dashed box). This relative energy was also evaluated by structural optimization in DMSO solvent calculation, showing a consistence of +5.7 kcal/mol (Table S2). Subsequently, the peroxide intermediate **4** undergoes O–O bond cleavage with the assistance of solvent DMSO via **TS2** (Δ*G* ^⧧^ = 24.4 kcal/mol), followed by a low-barrier protonation to afford the final hydroxylated product **6**. It is unfavorable for the DMSO-assisted O−O bond cleavage after protonation (see **TS5**: Δ*G* ^⧧^= 44.2 kcal/mol in Figure S3).

**Figure 1:**
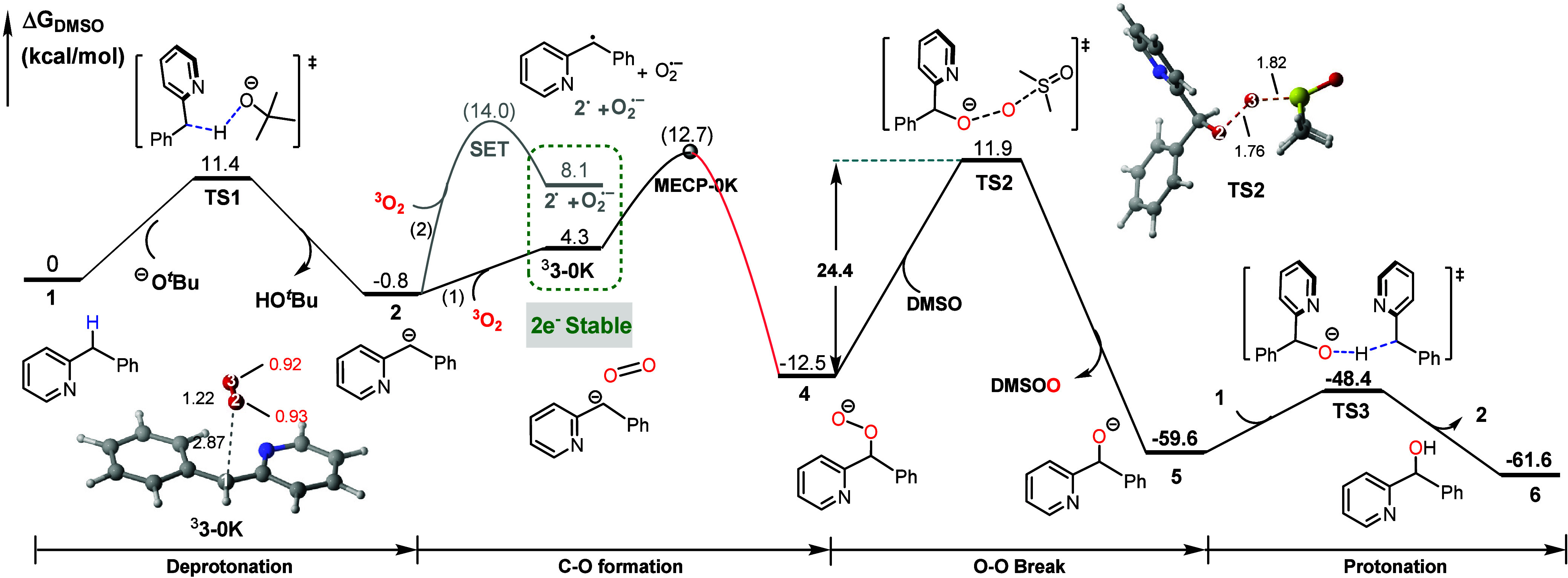
Free-energy profile of 2-benzylpyridine hydroxylation by the *t*-butoxide ion. The energies shown in brackets is electronic energies. In 3D structures, NPA spin population shown in the red, and bond length (Å) shown in black, the same as below.

### (b) Radical Mechanism: K^+^ Cluster Model

In contrast to no radical observation in reaction **I**, the other group employed 3 equiv of KO^*t*^Bu in reaction **II**, enabling the detection of radical. Considering that excess base may promote the aggregation of KO^*t*^Bu to form clusters, and given that KO^*t*^Bu typically exists as cubic tetramers,^58–61^ the high concentration of KO^*t*^Bu is not easy to induce the dissociation of the tetramer **[KO** ^***t***^ **Bu]**_**4**_ (Figure S4). Although reaction models for KO^*t*^Bu were tested from monomer to tetramer (Figures S5–S7), the most stable tetrameric model is used in the following discussions (Figure 2). the formation of biradical intermediate ^3^ **3–4K** (1.01 spin population on the O_2_) is thermodynamically favorable. This radical pathway is as follows: the deprotonation of substrate **1** over an energy barrier of 15.5 kcal/mol generates **2–4K**, which undergoes an SET with O_2_ to form stable biradical intermediate ^3^ **3–4K** (−6.1 kcal/mol relative to ^3^ **6–4K**). This relative energy was also evaluated by structural optimization in DMSO solvent calculation, showing a consistence of −7.7 kcal/mol (Table S2). This carbon radical intermediate should be experimentally detected. Conversely, the carbanion pathway is disfavored for a less-stable intermediate (^3^ **6–4K**) and must overcome a high energy barrier (**MECP′-4K**: 31.6 kcal/mol). The independent gradient model based on Hirshfeld partition (IGMH) analysis^62^ shows that the attractive interaction between K^+^ ions and the O_2_^• –^ radical makes the ^3^ **3–4K** more stable. Additionally, the rate-determining step for tetrameric KO^*t*^Bu involves an O–O bond cleavage with an energy barrier of 21.7 kcal/mol (**TS2–4K**), which is lower than that for anionic *tert*-butoxide (24.4 kcal/mol, **TS2** in Figure 1). It can rationalize the relatively efficient reaction **II** at the condition of 30 °C and 2 min, while it is 50 °C and 2 h in reaction **I**. This kind of radical pathway induced by the K^+^ cluster could be named as the Alkali Metal Reagent Promoted Proton Transfer-Electron Transfer (A-PTET) mechanism.

**Figure 2:**
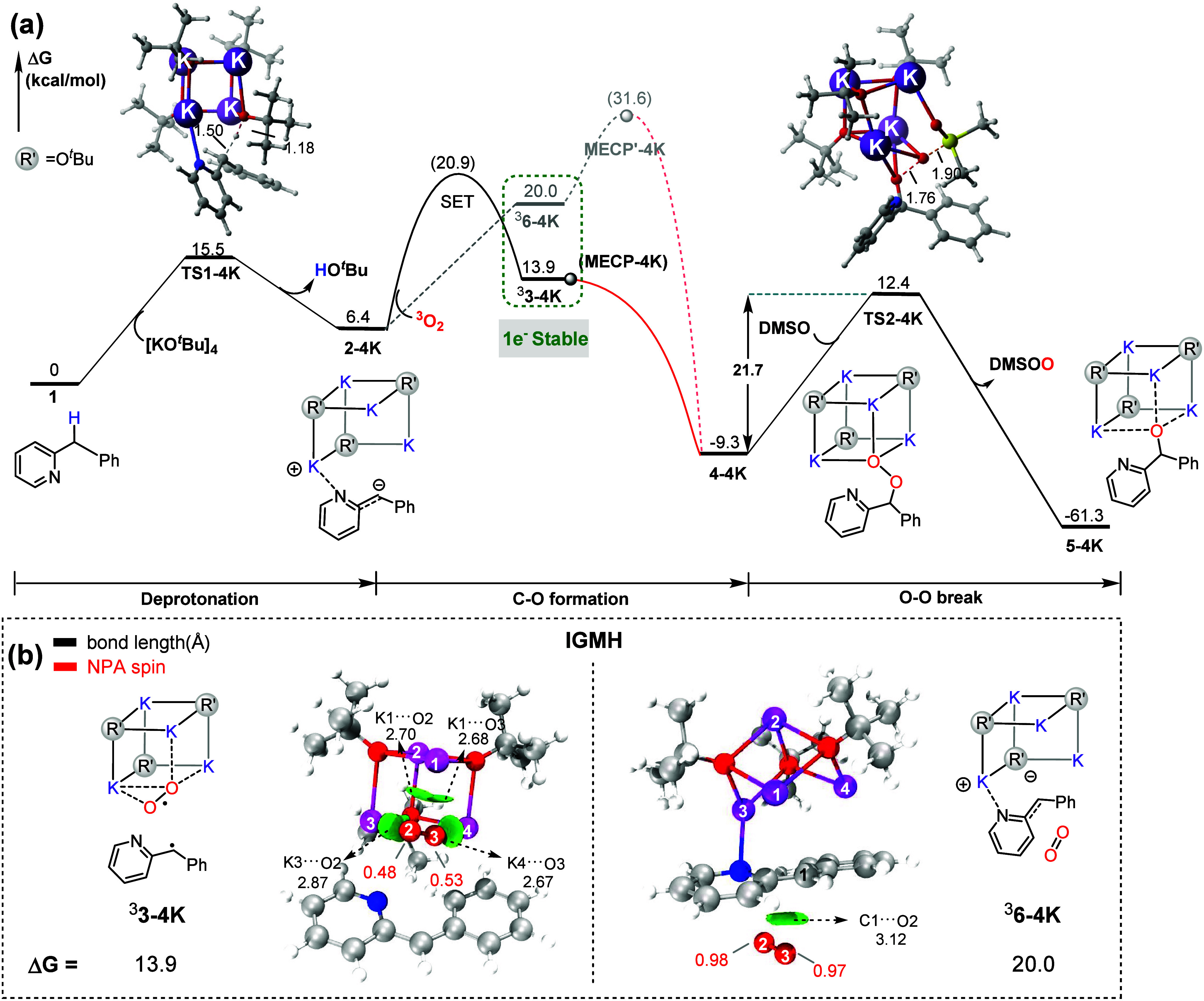
(a) Free-energy profile of 2-benzylpyridine hydroxylation by the **[KO** ^***t***^ **Bu]**_**4**_ cluster. (b) Structures of key transition states in the reaction. IGMH analysis was performed for ^3^ **3–4K** and ^3^ **6–4K**.

### (c) The Origin of A-PTET

Based on these results, the influence on A-PTET was evaluated by comparing the thermodynamic energies of radical generation via oxygen reaction across different KO^*t*^Bu cluster with implicit and explicit DMSO models (see Figure 3, as well as Figures S8 and S9). Since the explicit DMSO model has a minor effect on the calculated energies, the discussion in the main text was conducted using the more concise implicit model. As the cluster number of KO^*t*^Bu increases, the SET process becomes more favorable. In the monomeric anionic state, the carbon radical intermediate is less stable than the carbanion intermediate (Δ*G* = +3.8 kcal/mol). However, with the increasing stability of **[KO** ^**t**^ **Bu]**_***n***_clusters (*n* = 1–4), the carbon radical intermediates become more stable (Δ*G* = −6.1 kcal/mol in the tetramer intermediate). Although the formation energy of the carbon radical intermediates in the trimeric state is −7.2 kcal/mol, the thermodynamic energy of the trimeric KO^*t*^Bu is 36.2 kcal/mol higher than that of the tetrameric KO^*t*^Bu (Figure S4). Therefore, discussions should be based on the tetramer. Compared with DMSO induced radical species,^29–33^ the thermodynamic energies of the radical generation were calculated involving DMSO and O_2_ (Figure S9). The most stable one is +9.4 kcal/mol, which could indeed trigger radicals under certain conditions (Figure 3c). However, radical initiation involving anion substrate **2** and O_2_ is more favorable, especially with the assistance of KO^*t*^Bu clusters (Figure 3a and 3b). Therefore, the DMSO-initiated radical mechanism is unlikely to be operative here.

**Figure 3:**
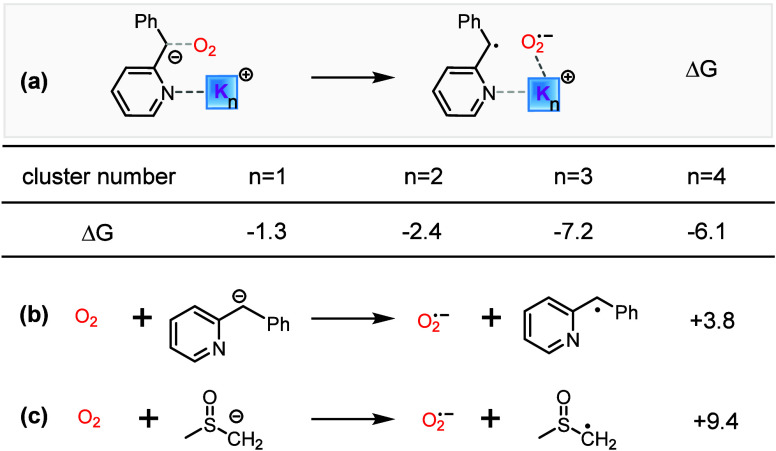
Calculated energetics of radical generations with O_2_ in the implicit DMSO model. (a) Carbanion in different **[KO** ^***t***^ **Bu]**_***n***_ clusters (*n* = 1, 2, 3, or 4); (b) Carbanion in the K^+^-free model; (c) Dimsyl anion. Energies are shown in kcal/mol.

Further analysis of the electronic structure (as shown in Figure 4) indicates that with the increasing number of the KO^*t*^Bu clusters, the spin population on the oxygen atoms (O2 and O3) decreases from 1.85 to 1.01, while that on the substrate increases from 0.15 to 0.95. Increasing the cluster number of KO^*t*^Bu enhances the biradical character of intermediate ^3^ **3-nK**, indicating that the O_2_^•–^ radical within this structure can be stabilized by the KO^*t*^Bu cluster. In contrast, the O_2_^•–^radical lacks cation stabilization in the anionic model, inhibiting the SET mechanism and thus favoring the carbanion pathway. Moreover, molecular electrostatic potential (ESP) analysis shows that the K^+^ cluster enhances the positive electrostatic field around oxygen atoms O2 and O3. This is evidenced by the average ESP on their van der Waals surfaces becoming less negative from −74.5 to −13.1 kcal/mol on O2. The above analysis indicates that increasing the amount of KO^*t*^Bu leads to a stronger positive electric field in the corresponding cluster, which enable to stabilize the O_2_^•–^radical thereby triggering radical generation as the single-electron transfer in the A-PTET mechanism.

**Figure 4:**
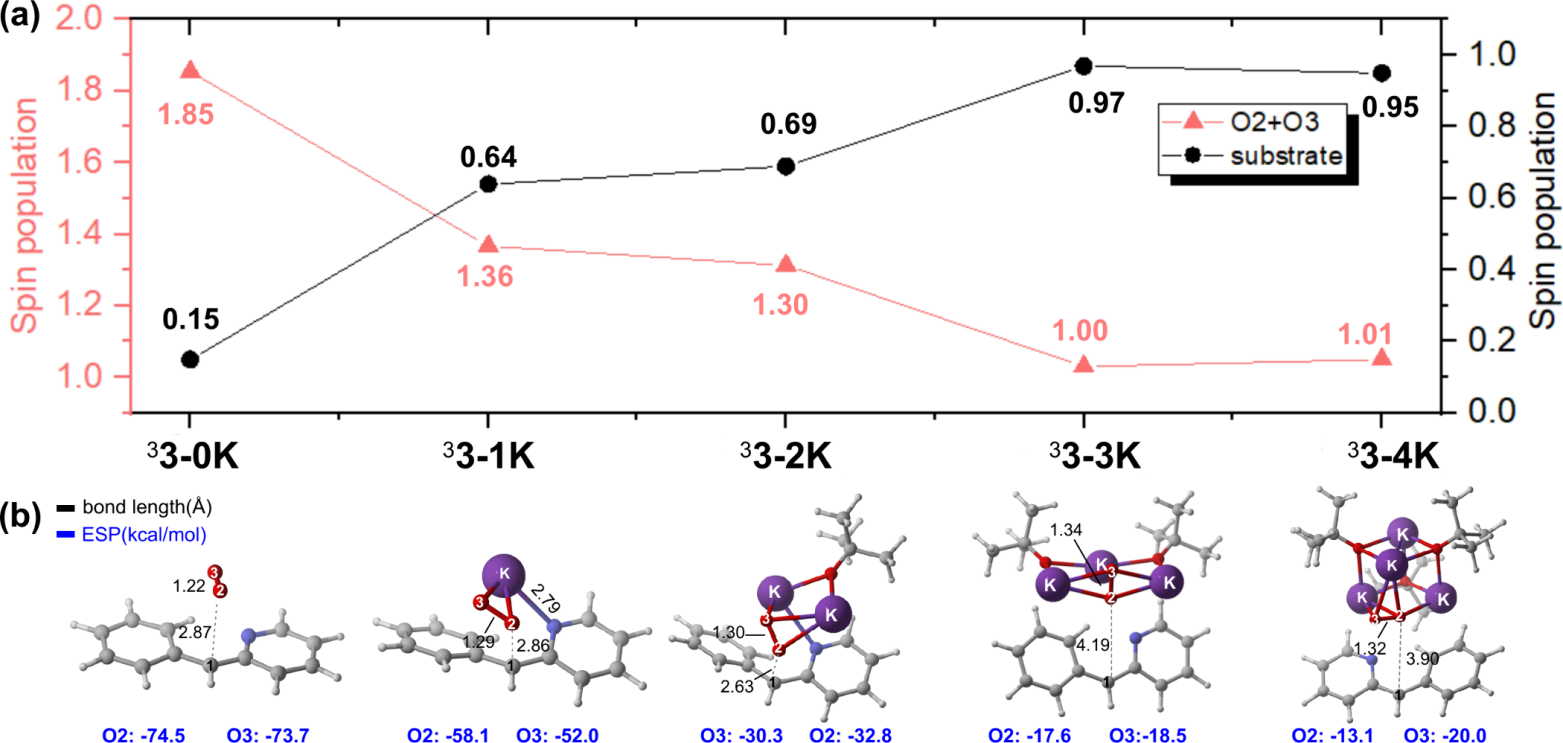
Structures and spin population of ^3^ **3-nK (** *n* = 0,1,2,3 and 4). Substrate: The sum spin population of the 2-pyridinyl benzylic anion. The electrostatic potential (ESP) is shown in blue.

Additionally, the reaction PECs from the triplet state to the singlet state also support the change from the carbanion to the carbon radical pathways (A-PTET). As shown in Figure 5, in the anionic reaction model, the **^3^3–0K** intermediate holds a spin population of only 0.15 on the substrate, which may be insufficient to generate detectable free radicals. Moreover, as the reaction proceeds to **MECP-0K**), the relative instability of the state may lead to a rapid ISC process, resulting in undetectable radical species experimentally. In contrast, under the **[KO** ^***t***^ **Bu]**_**4**_ cluster effect, ^3^ **3–4K** and ^1^ **3–4K** complexes exhibit quite consistent spin populations of the substrate (0.95 and 0.97, respectively). The apparent single electron transfer was able to locate at the C–O bond formation from −6.3 to −1.6 amu^1/2^bohr in Figure 5b (C–O distances from 3.90 to 2.16 Å). These stable intermediates in the relatively long reaction path increase the likelihood of detecting free radicals. Therefore, excessive alkali-metal reagents may alternate the carbanion mechanism to the A-PTET mechanism, thereby facilitating the appearance of radical characteristics consistent with experimental observations.

**Figure 5:**
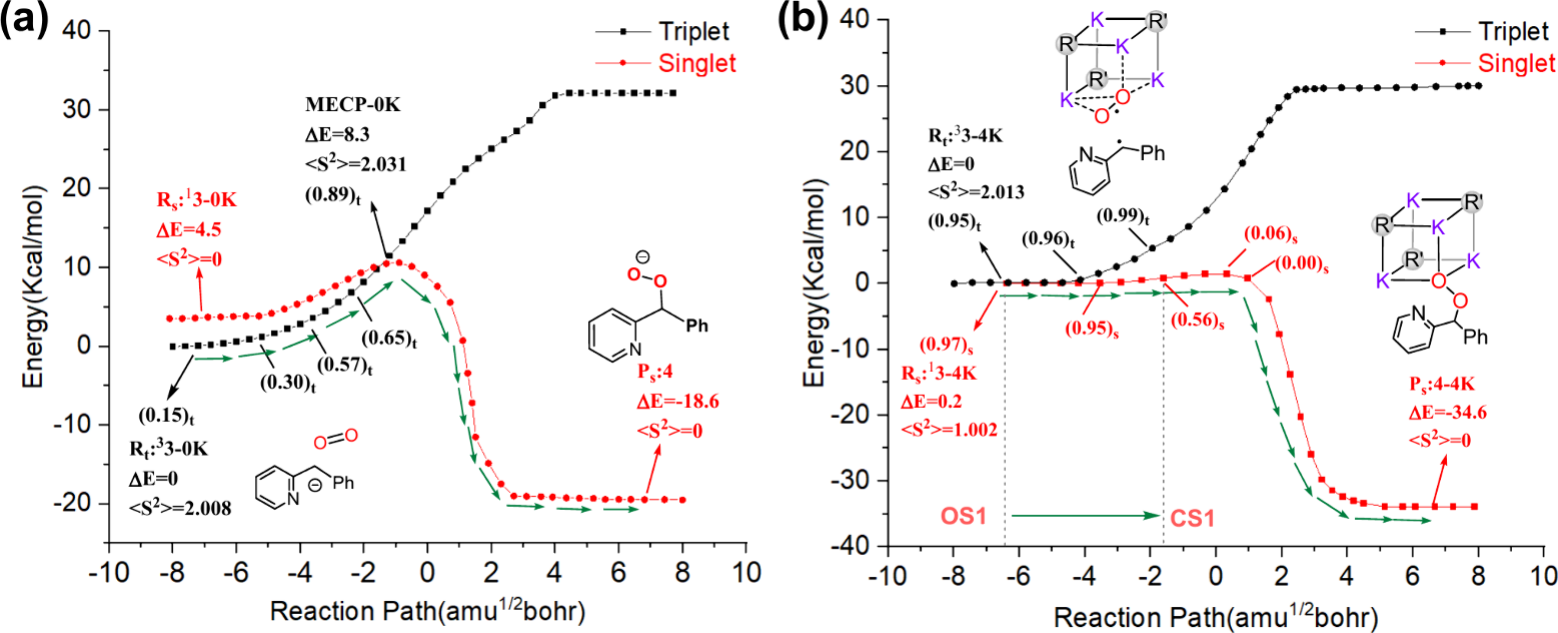
Singlet and triplet-PECs of the oxygenation for the evolution from open shell triplet to close shell singlet (a) ^3^ **3–0K** and (b) ^3^ **3–4K**. Spin populations of the substrate (2-pyridinyl benzylic anion) are shown in parentheses, with subscripts denoting spin states: t = triplet, s = singlet.

## 4 Conclusions

In summary, our DFT calculations investigated the mechanism of the aerobic hydroxylation of 2-benzylpyridine under different equiv of KO^*t*^Bu (Figure 6). A new alkali-metal-reagent-promoted proton transfer-electron transfer (A-PTET) mechanism was proposed under excess KO^*t*^Bu, leading to a carbon radical pathway in agreement with the experimentally detected radical signals. This is entirely different from the carbanion mechanism, with the catalytic amount of KO^*t*^Bu without radical signals. Furthermore, in the A-PTET mechanism, as the number of KO^*t*^Bu clusters increases, the characteristics of both the carbon and the O_2_^• –^ radicals become more pronounced, as supported by spin population, ESP, and PECs analysis. It is primarily attributed to the enhanced positive electric field generated by the increased number of KO^*t*^Bu clusters, which promotes single-electron transfer and stabilizes the O_2_^• –^ radical via the A-PTET mechanism. This study clarifies the essential regulatory role of alkali-metal clusters in single electron transfer processes and establishes a theoretical foundation for developing more aerobic reactions.

**Figure 6:**
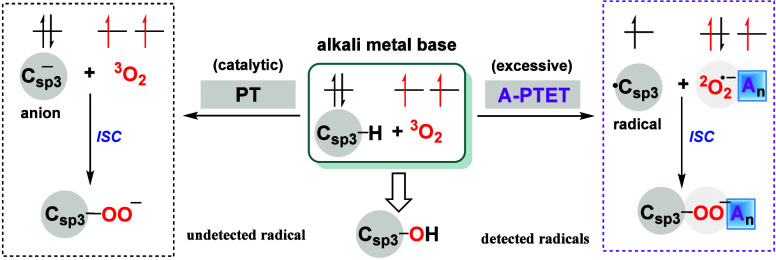
Comparison between the carbanion pathway and the carbon radical pathway (A-PTET mechanism).

## Supplementary Material



## Notes

Original data of figures in main text; the Gibbs free energy changes of the SET process; the total energies and Cartesian coordinates of calculated structures (PDF)
