# Supplementary material for: Potassium tert-Butoxide Promoted Proton Transfer-Electron Transfer in Aerobic C(sp3)–H Hydroxylations
Source: Inorg Chem. 2026 Apr 28;65(18):10179–86. doi: 10.1021/acs.inorgchem.6c00906 (PMC13169361; doi:10.1021/acs.inorgchem.6c00906)
Supplement: Supplementary file 1 [file ic6c00906_si_001.pdf]

Supporting Information

Potassium *tert*-Butoxide Promoted Proton Transfer-Electron Transfer in Aerobic C(sp^3)–H Hydroxylations

Changming Li[a], Hongdan Zhu[a] and Qian Peng*[a,b]

[a] State Key Laboratory of Elemento-Organic Chemistry, Tianjin Key Laboratory of Biosensing and Molecular Recognition, College of Chemistry, Frontiers Science Center for New Organic Matter, Nankai University, Tianjin 300071, China.

[b] Haihe Laboratory of Sustainable Chemical Transformations, Tianjin 300192, China

* Corresponding authors

E-mail: qpeng@nankai.edu.cn

Contents

1. Computational Details of Conformational Search....S3
2. Benchmark of Density Functionals S6
3. The Gibbs free energy changes ($\Delta G^{\neq}_{SET}$) for the SET reaction. S11
4. Additional Tables and Figures S13
5. Computed Energies of Optimized Structure....S19
6. References....S21
7. Cartesian Coordinates of the Optimized Structures....S22

List of Figures

Figure S1.the conformational search of **^{3}3-nK (n =1-4)** S5
Figure S2. Schematic plot of reorganization energy S12
Figure S3. Free energy profile for the conversion of peroxide intermediate **4** to product **6**. S13
Figure S4. Dissociation of potassium tert-butoxide.... ....S14
Figure S5. Free energy profile of 2-benzylpyridine hydroxylation by KO^{t}Bu.....................................S14
Figure S6. Free energy profile of 2-benzylpyridine hydroxylation by dimer KO^{t}Bu... ....S15
Figure S7. Free energy profile of 2-benzylpyridine hydroxylation by tetramer KO^{t}Bu. S15
Figure S8. Calculated energetics of radical generations with O_2. in the explicit DMSO model. S16
Figure S9. DFT-calculated energies of other radical initiation mechanisms. S17

List of Tables

Table S1. The Benchmark of Density Functionals...S6
Table S2. Comparison of gibbs free energy between gas-phase structure optimization and solvated structure optimization. S7
Table S3. The Gibbs free energy changes ($\Delta G^{\neq}_{SET}$) of the SET process between **2-nK** and $^3O_2$ in DMSO solvent S13

1. Computational Details of Conformational Search

The detailed conformational search protocol was carried out as follows: conformational search was implemented for molecular dynamics structural sampling with the xtb program at the GFN0–xTB level.[1] Based on the periodic annealing method, the simulation temperature was set to 500 K to ensure rapid conformational changes of the molecules during the simulation and sufficient sampling within a limited time for the acquisition of locally optimal structures. The simulation time was set to 100 ps, with trajectory frames written to the trajectory file every 50 fs and a time step of 1 fs, thus generating a dynamic trajectory containing 2000 structures. On the basis of these structures, the CREST program[2] was used to perform normal geometric optimization for structural convergence at the GFN0–xTB level with the default convergence criteria of xtb. Subsequently, structural screening and deduplication were conducted via the isostat program, with the top 20 structures with the lowest energies retained. Thereafter, the Molclus program[3] was employed to call the xtb program for batch optimization and frequency analysis at the GFN2–xTB level combined with the implicit solvation model. A further round of structural screening and deduplication was performed using the isostat program, yielding 5 distinct structures with the lowest energies. Finally, the Gaussian 16 program was adopted to calculate the accurate solvation free energies of these 5 structures with the method of SMD(DMSO)-CAM-B3LYP-D3/6-311++G(d,p)/SDD(K)//CAM-B3LYP-D3/6-31G(d,p)-SDD(K). The Boltzmann distribution of the conformations was obtained from the energy comparison of conformational search results for different numbers of KO^{t}Bu clusters, as presented below.

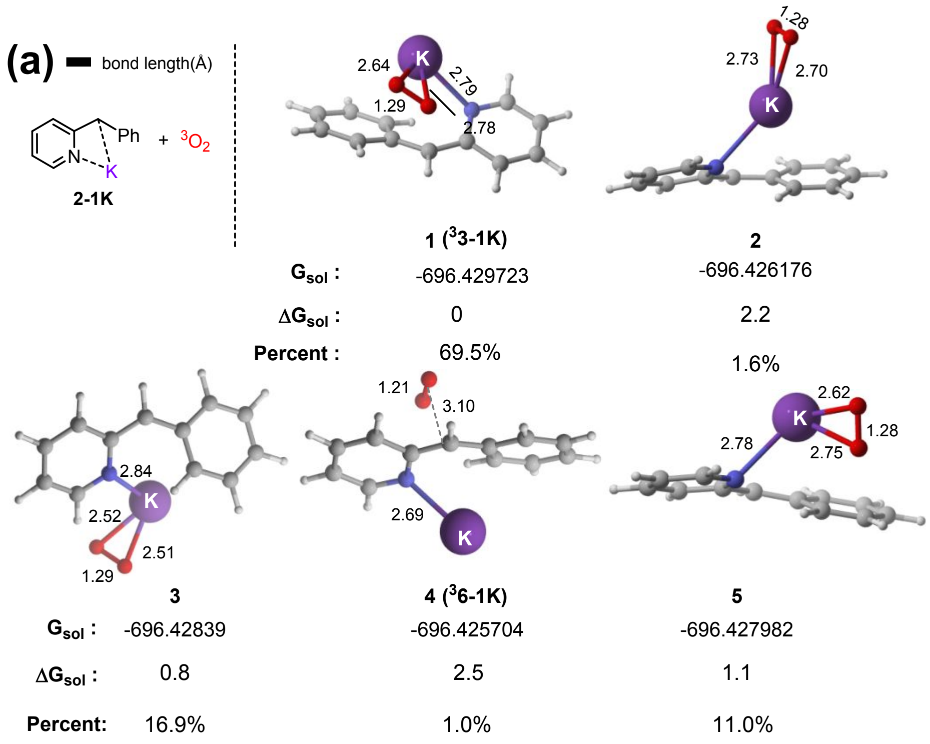

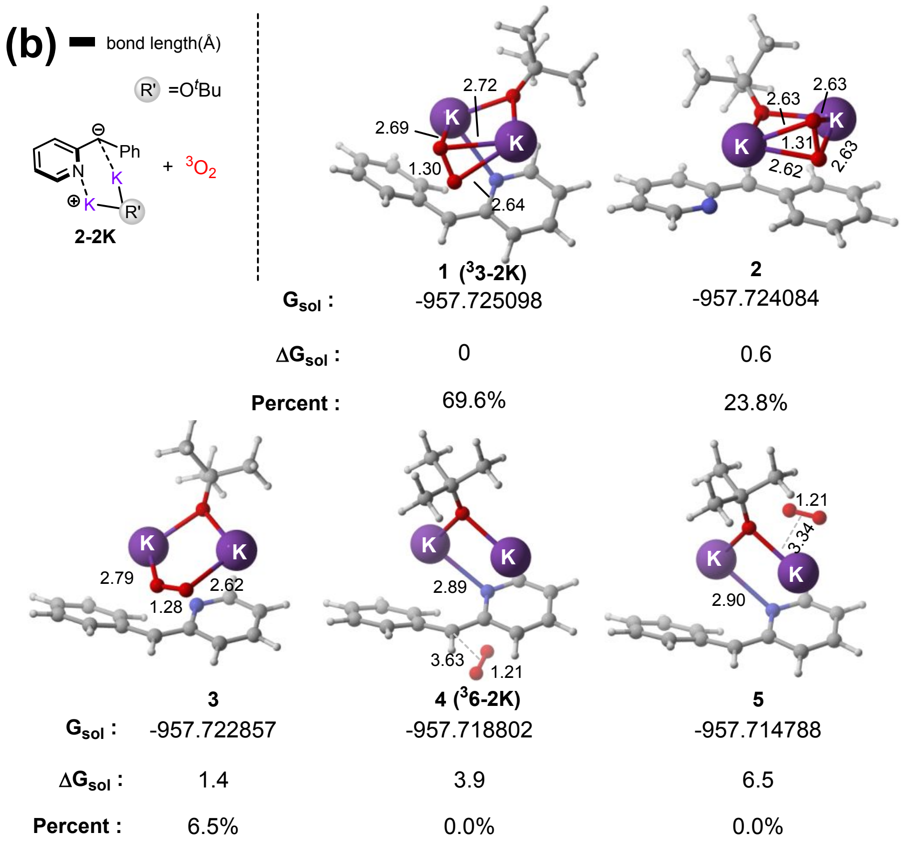
(b)
bond length(Å)
R' =O^{t}Bu
Ph
K
K
R'
2-2K
+ $^3O_2$
2.72
2.69
1.30
2.64
1 (^{3}3-2K)
2.63
2.63
1.31
2.62
2.63
2
G_{sol} : -957.725098 -957.724084
ΔG_{sol} : 0 0.6
Percent : 69.6% 23.8%
2.79
1.28
2.62
3
2.89
3.63
1.21
4 (^{3}6-2K)
1.21
3.34
2.90
5
G_{sol} : -957.722857 -957.718802 -957.714788
ΔG_{sol} : 1.4 3.9 6.5
Percent : 6.5% 0.0% 0.0%

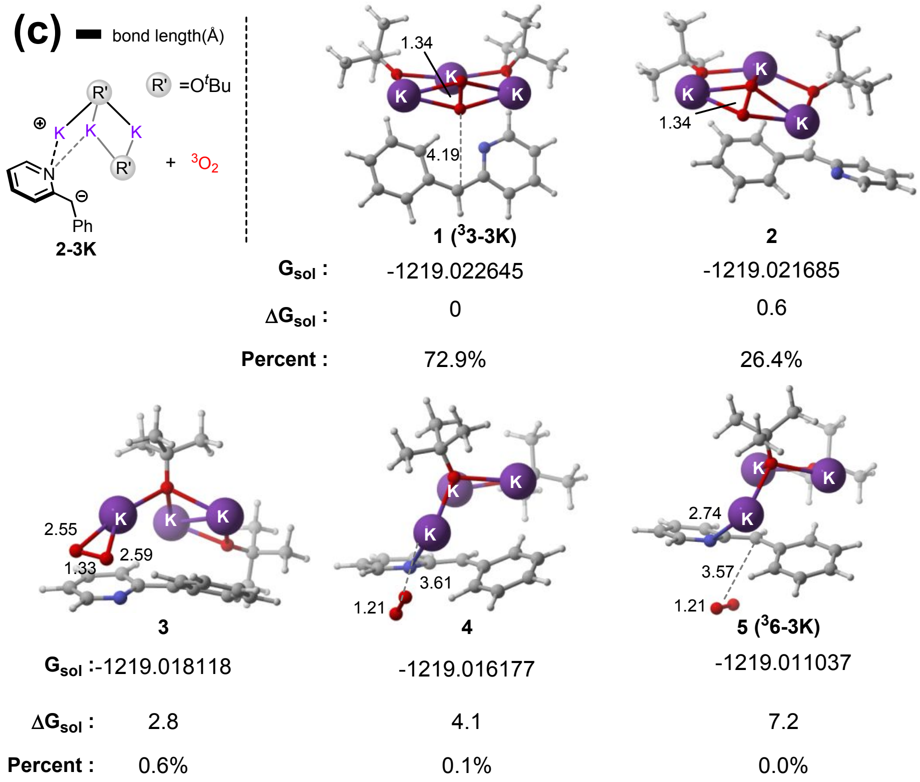
(c)
bond length(Å)
R' =O^{t}Bu
R'
K
K
K
R'
Ph
2-3K
+ $^3O_2$
1.34
4.19
1 (^{3}3-3K)
1.34
2
G_{sol} : -1219.022645 -1219.021685
ΔG_{sol} : 0 0.6
Percent : 72.9% 26.4%
2.55
1.33
2.59
3
1.21
3.61
4
2.74
3.57
1.21
5 (^{3}6-3K)
G_{sol} :-1219.018118 -1219.016177 -1219.011037
ΔG_{sol} : 2.8 4.1 7.2
Percent : 0.6% 0.1% 0.0%

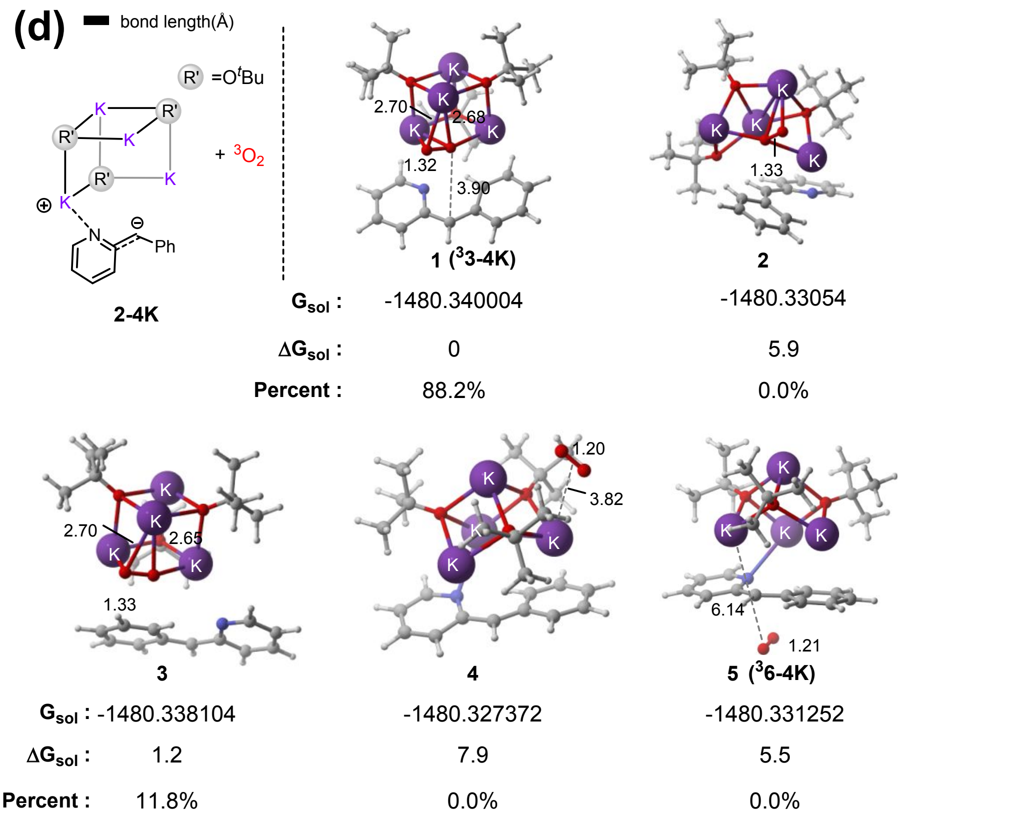

Figure S1. The five lowest-energy intermediate conformations obtained from the conformational search during the binding of O_2 to the substrate in the presence of (a) monomer KO^{t}Bu. (b) dimer KO^{t}Bu. (c) trimer KO^{t}Bu. (d) tetramer KO^{t}Bu.

2. Benchmark of Density Functionals

Four density functionals, namely, CAM-B3LYP-D3, B3LYP[4]-D3, ωB97XD,[5] and PBE0[6]-D3 were adopted in computing the Gibbs free energy barriers for transition states (**TS2** and **TS2-3K**) and intermediate ($\mathbf{2}^{\cdot} + \mathbf{O_2^{\cdot-}}$, ^{3}**3-0K**, **6-3K** and ^{3}**3-3K**). Different density functionals provided similar results, suggesting that the results with CAM-B3LYP-D3 functional are reliable.

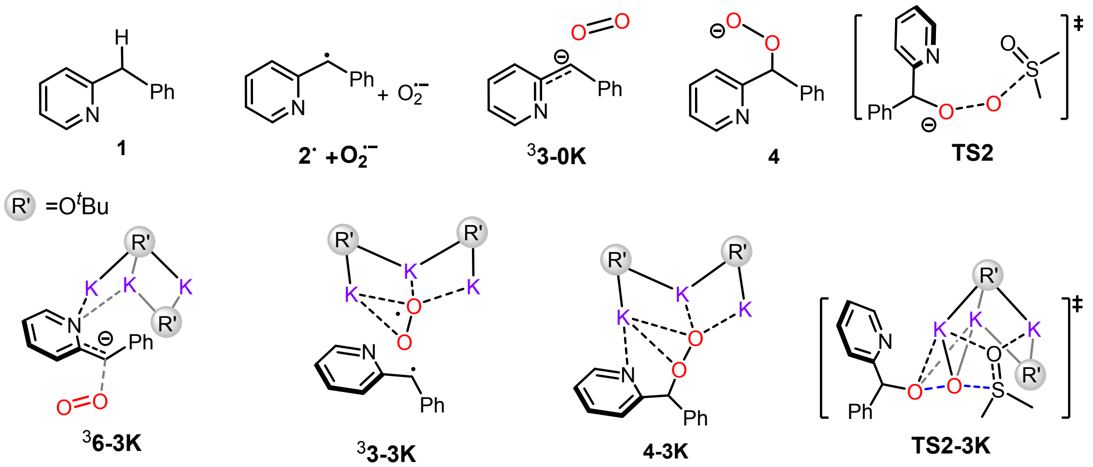

Table S1. Benchmark of Density Functionals[a].

DF	CAM-B3LYP-D3[b]	B3LYP-D3[b]	wB97XD[b]	PBE0-D3[b]	CAM-B3LYP-D3[c]
ΔG (**1**)	**0**	**0**	**0**	**0**	**0**
ΔG (**4**)	**-12.1**	**-9.4**	**-11.3**	**-10.5**	**-10.8**
ΔG (**TS2**)	**11.9**	**12.6**	**12.5**	**13.6**	**10.5**
ΔG (**4-3K**)	**-16.5**	**-15.3**	**-14.9**	**-14.2**	**-14.6**
ΔG (**TS2-3K**)	**3.5**	**5.6**	**6.2**	**6.0**	**4.7**
ΔG ($\mathbf{2}^{\cdot} + \mathbf{O_2^{\cdot-}}$)	**8.1**	**7.2**	**7.6**	**8.7**	**10.6**
ΔG (^{3}**3-0K**)	**4.3**	**3.7**	**3.4**	**4.0**	**5.1**
ΔG (^{3}**6-3K**)	**15.5**	**16.0**	**14.8**	**15.8**	**15.9**
ΔG (^{3}**3-3K**)	**8.2**	**9.3**	**8.0**	**7.9**	**7.7**

[a]Energy shown in kcal/mol at the with the DMSO solvent, (energies in kcal/mol, the same below).
[b]DF/SDD-6-311++G(d,p)//CAM-B3LYP-D3/SDD-6-31G(d,p) level was used for calculation in Gaussian 16.
[c]CAM-B3LYP-D3/ANO-RCC-VDZP//CAM-B3LYP-D3/SDD-6-31G(d,p) level was used for calculation in Beijing Density Functional (BDF) software.[7]

A total of 21 key structures, including the transition states and intermediates involved in the reaction, were selected. Geometric optimizations were performed with the CPCM solvation model, [8] followed by single-point energy calculations using the SMD solvation model. The results demonstrate that the computational approach adopted in this work, which combines geometric optimization in the gas phase with energy calculation based on the SMD model, is reliable.

Table S2. Comparison of gibbs free energy between gas-phase structure optimization and solvated structure optimization.

DF	SMD(DMSO)-CAM-B3LYP-D3/ 6-311++G(d,p)-SDD(K)//CAM-B3LYP-D3/6-31G(d,p)-SDD(K)	SMD(DMSO)-CAM-B3LYP-D3/ 6-311++G(d,p)-SDD(K)// CPCM(DMSO)-CAM-B3LYP-D3/6-31G(d,p)-SDD(K)
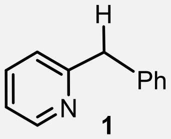 **1**	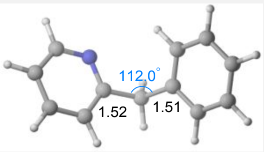	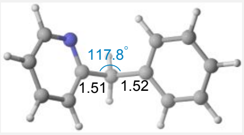
G_{solv}	-518.349333	-518.349793
ΔG_{solv}	**0**	**0**
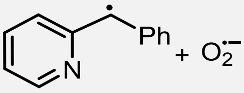 **2• +O2•–**	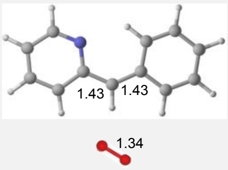	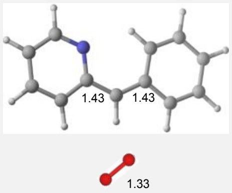
G_{solv}	-668.177564	-668.176818
$\Delta G^{\ddagger}_{solv}$	**8.1**	**9.2**
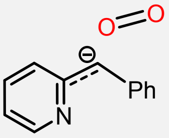 **³3-0K**	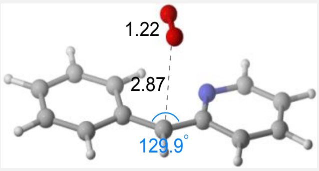	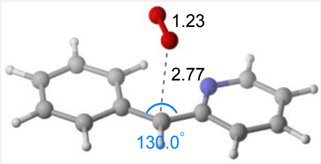
G_{solv}	-668.183604	-668.185857
ΔG_{solv}	**4.3**	**3.5**
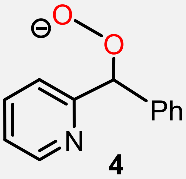 **4**	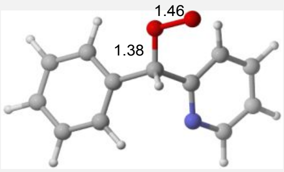	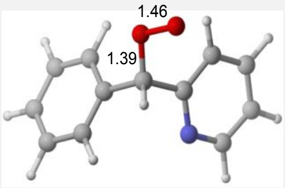
G_{solv}	-668.210523	-668.20867
ΔG_{solv}	**-12.1**	**-10.8**

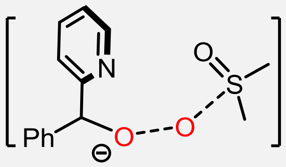 **TS2**	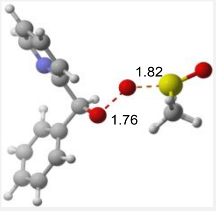	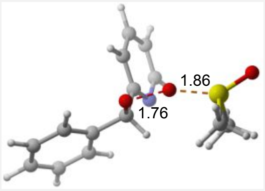
G_{solv}	-1221.317538	-1221.295548
$\Delta G^{\ddagger}_{solv}$	**24.4**	**26.1**
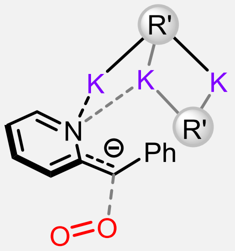 **^{3}6-3K**	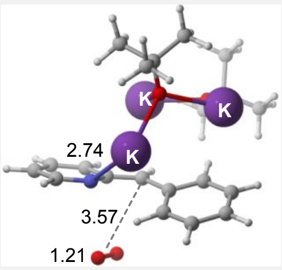	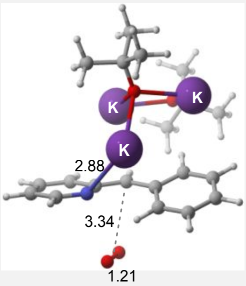
G_{solv}	-1219.00801	-1219.012897
ΔG_{solv}	**15.4**	**11.9**
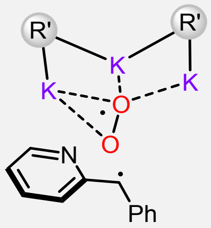 **^{3}3-3K**	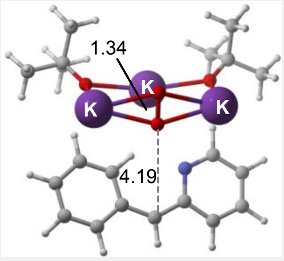	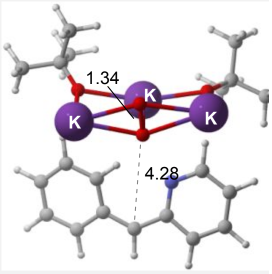
G_{solv}	-1219.019633	-1219.023028
ΔG_{solv}	**8.2**	**5.6**
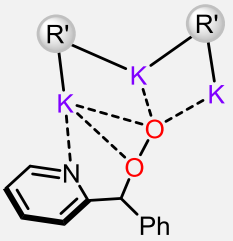 **4-3K**	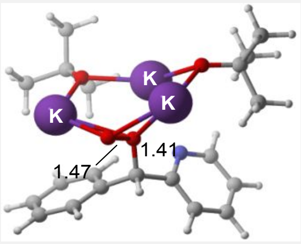	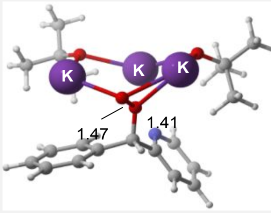
G_{solv}	-1219.059004	-1219.063136
ΔG_{solv}	**-16.5**	**-19.5**
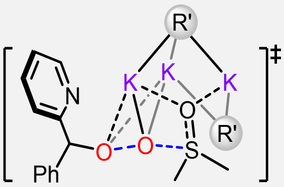 **TS2-3K**	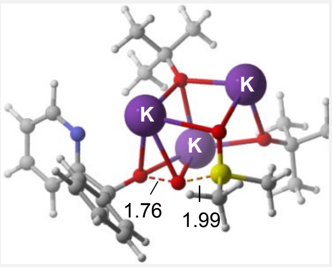	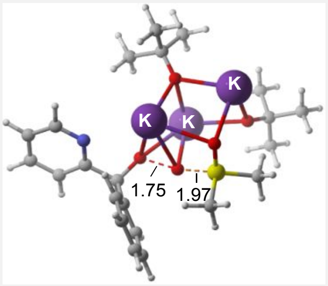
G_{solv}	-1772.17299	-1772.180716

$\Delta G^{\ddagger}_{solv}$	**20.1**	**18.2**
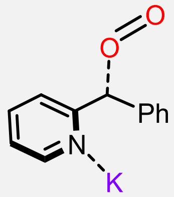<br>**^{3}6-1K**	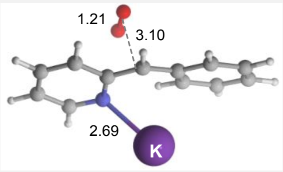	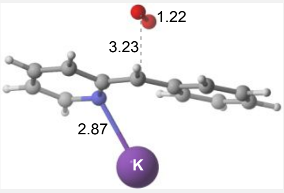
G_{solv}	-696.422692	-696.427372
ΔG_{solv}	**11.2**	**8.6**
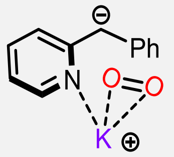<br>**^{3}3-1K**	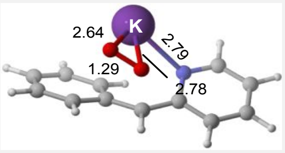	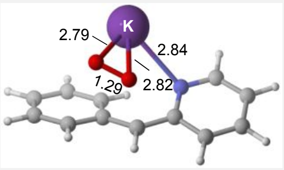
G_{solv}	-696.426711	-696.433177
$\Delta G^{\ddagger}_{solv}$	**8.7**	**4.9**
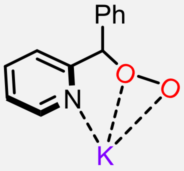<br>**4-1K**	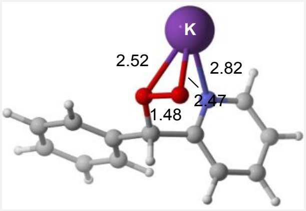	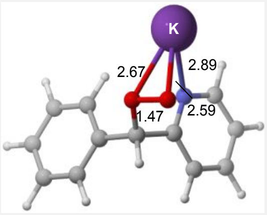
G_{solv}	-696.457755	-696.463902
ΔG_{solv}	**-10.7**	**-14.3**
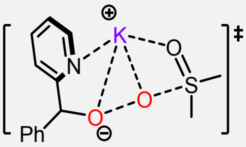<br>**TS2-1K**	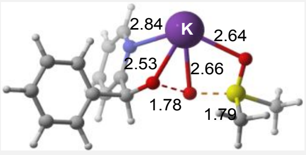	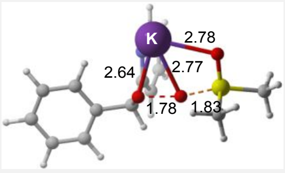
G_{solv}	-1249.573231	-1249.580855
$\Delta G^{\ddagger}_{solv}$	**19.1**	**18.6**
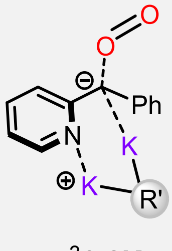<br>**^{3}6-2K**	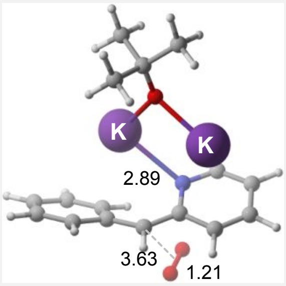	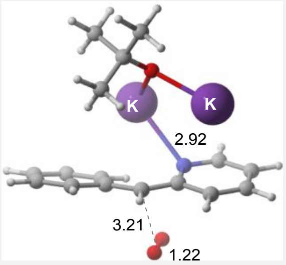
G_{solv}	-957.721351	-957.716292
ΔG_{solv}	**14.1**	**15.2**

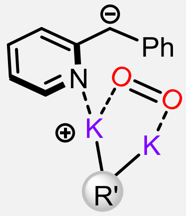

^{3}**3-2K**

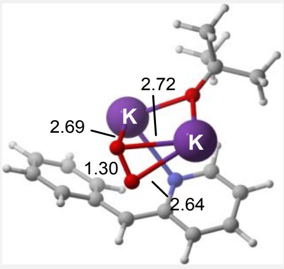

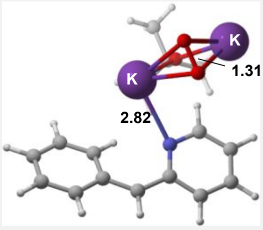

G_{solv}	-957.727647	-957.732426
ΔG_{solv}	**10.1**	**8.6**

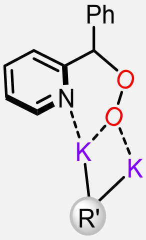

4-2K

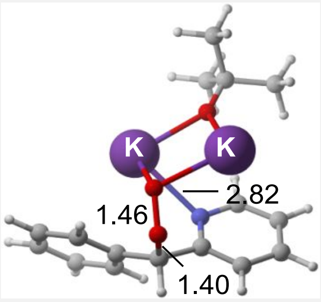

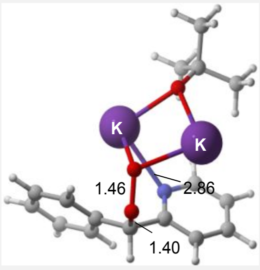

G_{solv}	-957.76392	-957.764663
ΔG_{solv}	**-12.6**	**-15.1**

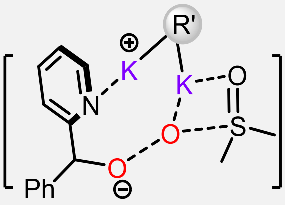

TS2-2K

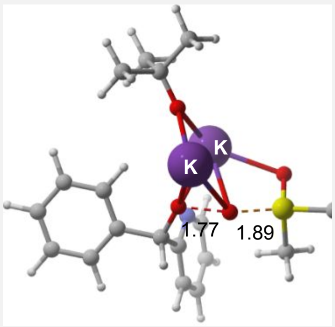

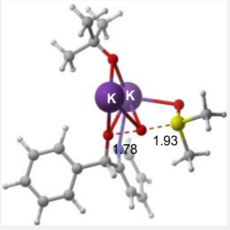

G_{solv}	-1510.875011	-1510.879958
$\Delta G^{\ddagger}_{solv}$	**21.8**	**19.6**

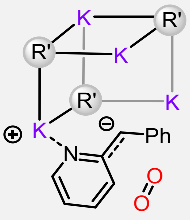

^{3}**6-4K**

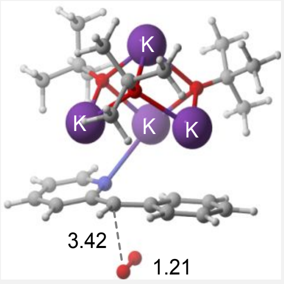

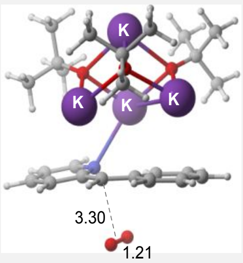

G_{solv}	-1480.326659	-1480.33269
ΔG_{solv}	**20.0**	**19.7**

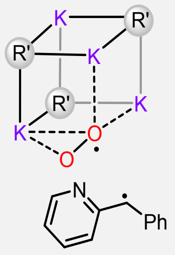

^{3}**3-4K**

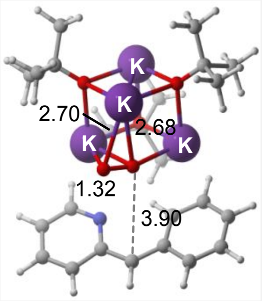

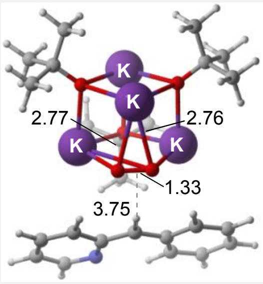

G_{solv}	-1480.336355	-1480.344981
ΔG_{solv}	**13.9**	**12.0**

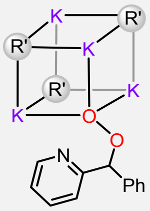

4-4K

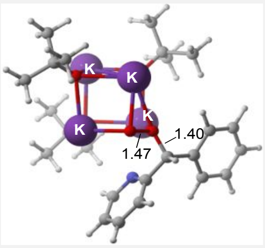

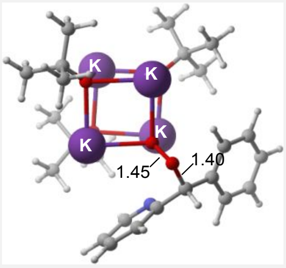

G_{solv}	-1480.373398	-1480.381083
ΔG_{solv}	**-9.3**	**-10.7**

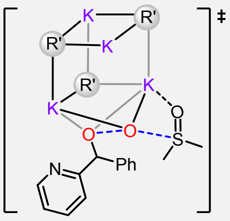

TS2-4K

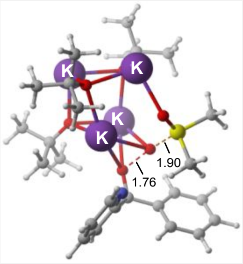

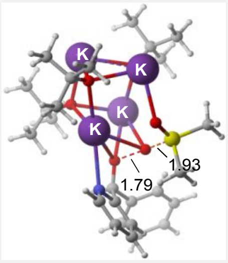

G_{solv}	-2033.48485	-2033.492195
$\Delta G^{\ddagger}_{solv}$	**21.7**	**23.0**

3. The Gibbs free energy changes ($\Delta G^{\neq}_{SET}$) for the SET reaction

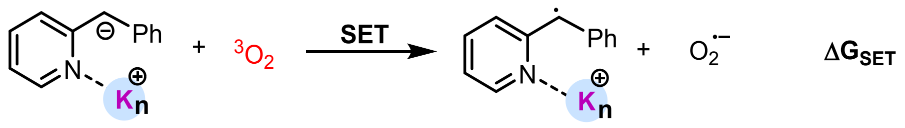

$$\Delta G^{\neq}_{SET} = \frac{(\lambda + \Delta G_{SET})^2}{4\lambda} (1)$$

λ includes the internal reorganization energy (λ_i) and the external reorganization energy (λ_e). The λ_i is divided into the donor reorganization energy (λ_D) and accepter reorganization energy (λ_A), λ_D is estimated by $E^{+*} - E^{+} + E^{*} - E$, λ_A is estimated by $E^{*} - E + E^{-*} - E^{-}$, As shown in Figure S2, E and E^{+}(E^{-}) are the total energies of stationary points of neural and ionic species, respectively. E* is the energy of the corresponding ionic form based on the neutral geometry, and E^{+*}(E^{-*}) is the energy of the corresponding neutral form based on the ionic geometry. The four energies are computed for both donor and acceptor.

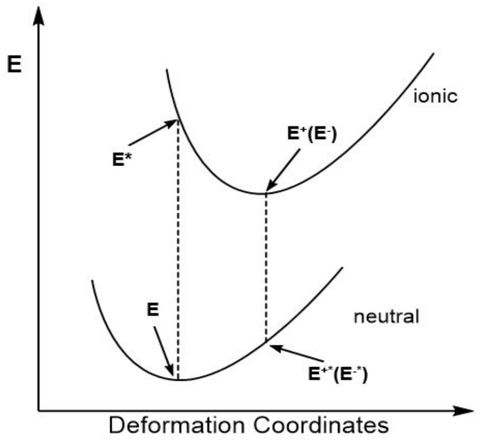

Figure S2 The calculation of $\Delta G^{\neq}_{SET}$ and schematic plot of reorganization energy.

$$\lambda_i = \lambda_D + \lambda_A (2)$$

$$\lambda_D = E^{+*} - E^{+} + E^{*} - E (3)$$

$$\lambda_A = E^{*} - E + E^{-*} - E^{-} (4)$$

The calculation of λ_e is performed based on the Marcus's two sphere model as shown in formula (5)[9]:

$$\lambda_e = (\Delta q)^2 \left(\frac{1}{2d_D} + \frac{1}{2d_A} - \frac{1}{d_{DA}}\right)\left(\frac{1}{\epsilon_{op}} - \frac{1}{\epsilon_0}\right) (5)$$

Where d_D and d_A represent the radii of the donor and acceptor, respectively, and d_{DA} denotes the mass-center distance between them. $\epsilon_{op}(2.2)$ and $\epsilon_0(47.2)$ are the optical and static dielectric constants of the solvent, respectively, Δq corresponds to the amount of charge transferred.

Table S3. The Gibbs free energy changes ($\Delta G^{\neq}_{SET}$) of the SET process between **2-nK** (n= 0,1,2,3 and 4) and O_2 in DMSO solvent.

2-nK	2-0K	2-1K	2-2K	2-3K	2-4K
**λ(kcal/mol)**	39.5	40.3	42.9	44.7	46.1
**ΔG_{SET}(kcal/mol)**	8.8	8.0	7.2	5.9	5.4
**$\Delta G^{\neq}_{SET}$(kcal/mol)**	14.8	14.5	14.7	14.3	14.4

4. Additional Tables and Figures

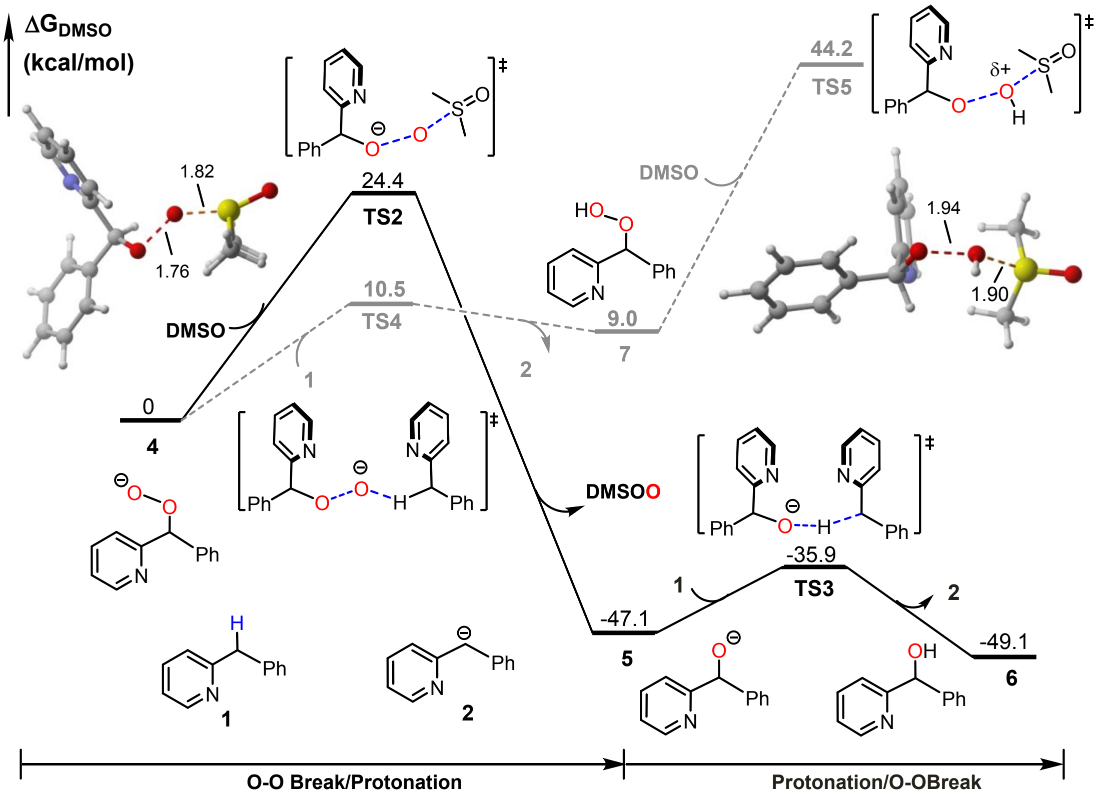

Figure S3. Free energy profile for the conversion of peroxide intermediate **4** to product **6**. The black values: bond length (in Å)

Figure S4 structures:

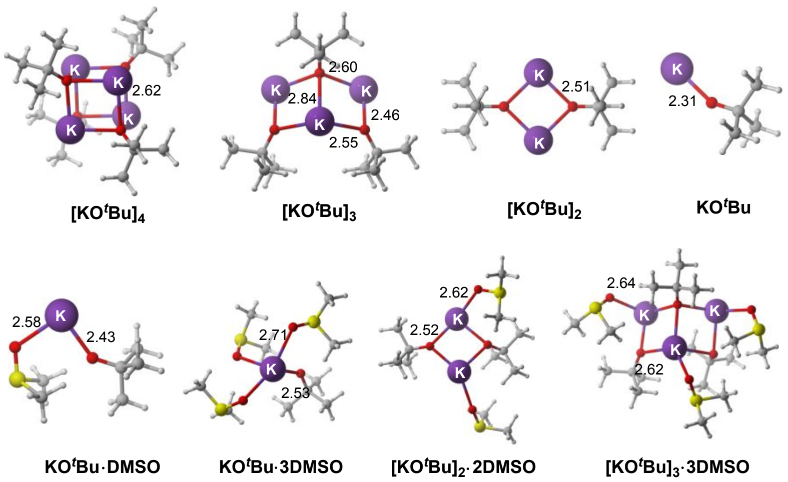

$[KO^tBu]_4$	$\longrightarrow$ $[KO^tBu]_3$ + KO^tBu	ΔG = +36.2
$[KO^tBu]_4$ + DMSO	$\longrightarrow$ $[KO^tBu]_3$ + $KO^tBu \cdot DMSO$	ΔG = +32.6
$[KO^tBu]_4$ + 3 DMSO	$\longrightarrow$ $[KO^tBu]_3$ + $KO^tBu \cdot 3DMSO$	ΔG = +31.8
$[KO^tBu]_4$ + 3 DMSO	$\longrightarrow$ $[KO^tBu]_3 \cdot 3DMSO$ + KO^tBu	ΔG = +37.8
$[KO^tBu]_4$	$\longrightarrow$ 2 $[KO^tBu]_2$	ΔG = +27.1
$[KO^tBu]_4$ + 4 DMSO	$\longrightarrow$ 2 ($[KO^tBu]_2 \cdot 2DMSO$)	ΔG = +26.3

Figure S4. Dissociation of tetrameric potassium tert-butoxide into trimer, dimer, and monomer in DMSO. Energies shown in kcal/mol. In 3D structures, bond length (Å) shown in black.

In the presence of coordinating DMSO solvent molecules, the tetrameric KO^tBu remains the most stable structure in concentrated KO^tBu solution. Furthermore, explicit DMSO solvent was found to stabilize the monomer to a greater extent (from +36.2 kcal/mol to +31.8 kcal/mol), whereas its stabilizing effect on the dimer was limited (from +27.1 kcal/mol to +26.3 kcal/mol).

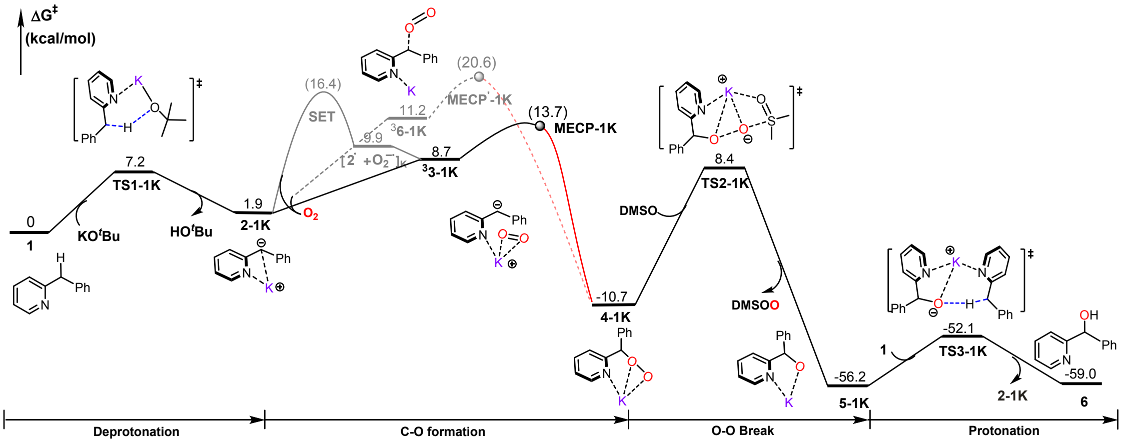

Figure S5. Free energy profile of 2-benzylpyridine hydroxylation by KO^tBu. the energy in parentheses is electron energy.

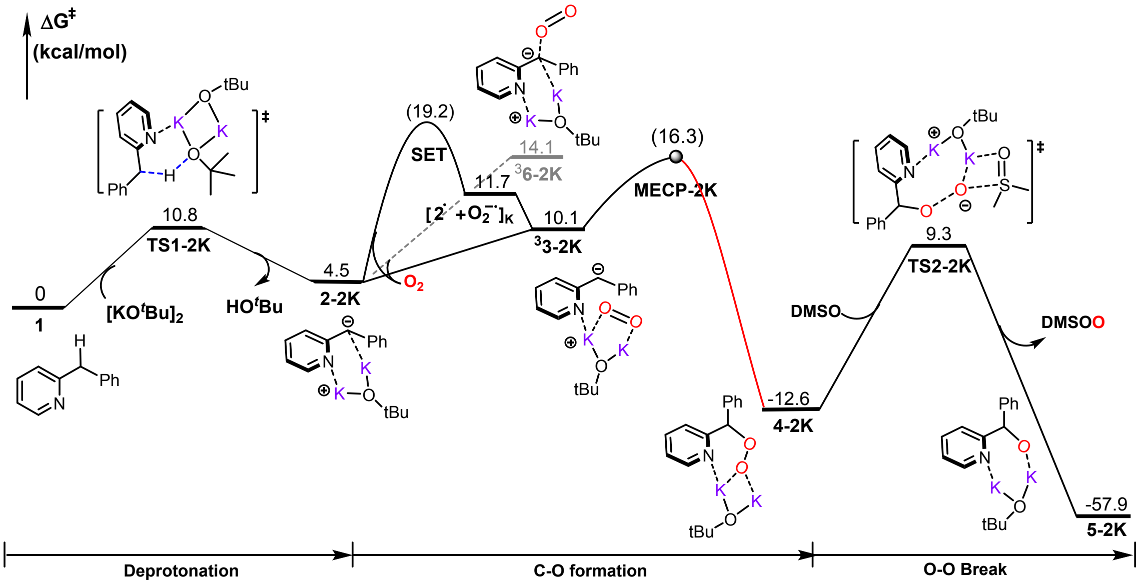

Figure S6. Free energy profile of 2-benzylpyridine hydroxylation by $[KO^tBu]_2$. the energy in parentheses is electron energy.

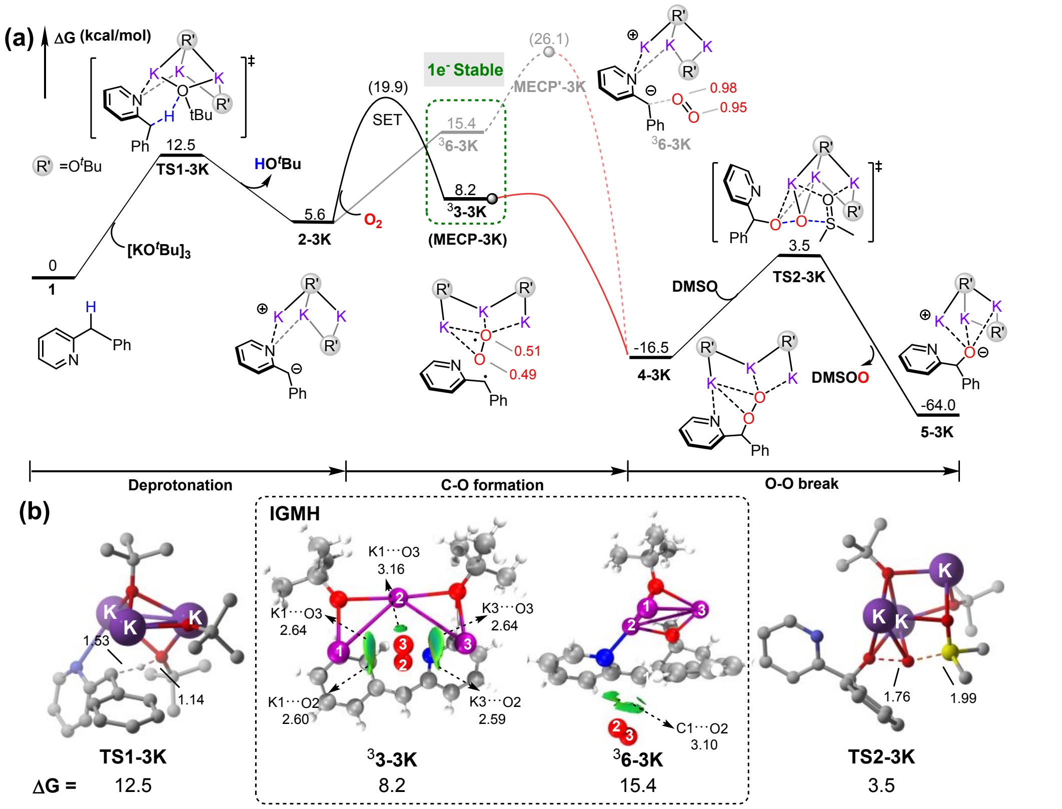

Figure S7. Free energy profile of 2-benzylpyridine hydroxylation by $\mathbf{[KO^tBu]_3}$.

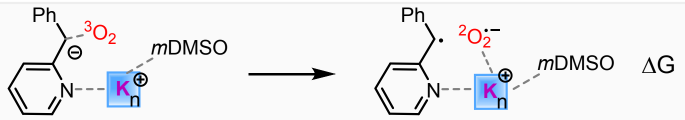

without explicit DMSO coordination (m=0):

cluster number	n=1	n=2	n=3	n=4
ΔG	-1.3	-2.4	-7.2	-6.1

(a) The monomer (n=1):

DMSO number	m=0	m=1	m=2	m=3
ΔG	-1.3	-2.5	-1.2	-0.2

(b) The dimer (n=2):

DMSO number	m=0	m=2	m=4
ΔG	-2.4	-1.6	-1.1

(c) The trimer (n=3):

DMSO number	m=0	m=3
ΔG	-7.2	-5.2

(d)

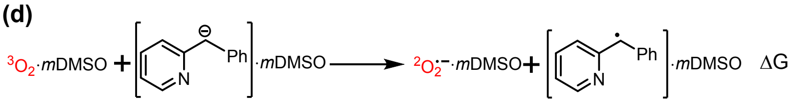

DMSO number	m=0	m=1	m=2	m=3	average ΔG
ΔG	+3.8	+2.7	+2.0	+1.7	+2.5

(e)

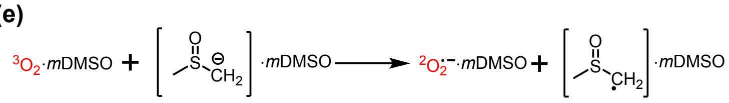

DMSO number	m=0	m=1	m=2	m=3	average ΔG
ΔG	+9.4	+10.5	+8.1	+8.6	+9.2

Figure S8. Calculated energetics of radical generations with O_2. in the explicit DMSO solvent model. (a) the monomer; (b) the dimer; (c) the trimer. (d) Carbanion in the K^+-free model; (e) Dimsyl anion. Energies are given in kcal/mol.

The calculation results show that as the coordination number of DMSO increases, the energetics of the radical generation process change little (within 2.1 kcal/mol), and the trend in radical generation remains consistent with that obtained from the implicit solvent model.

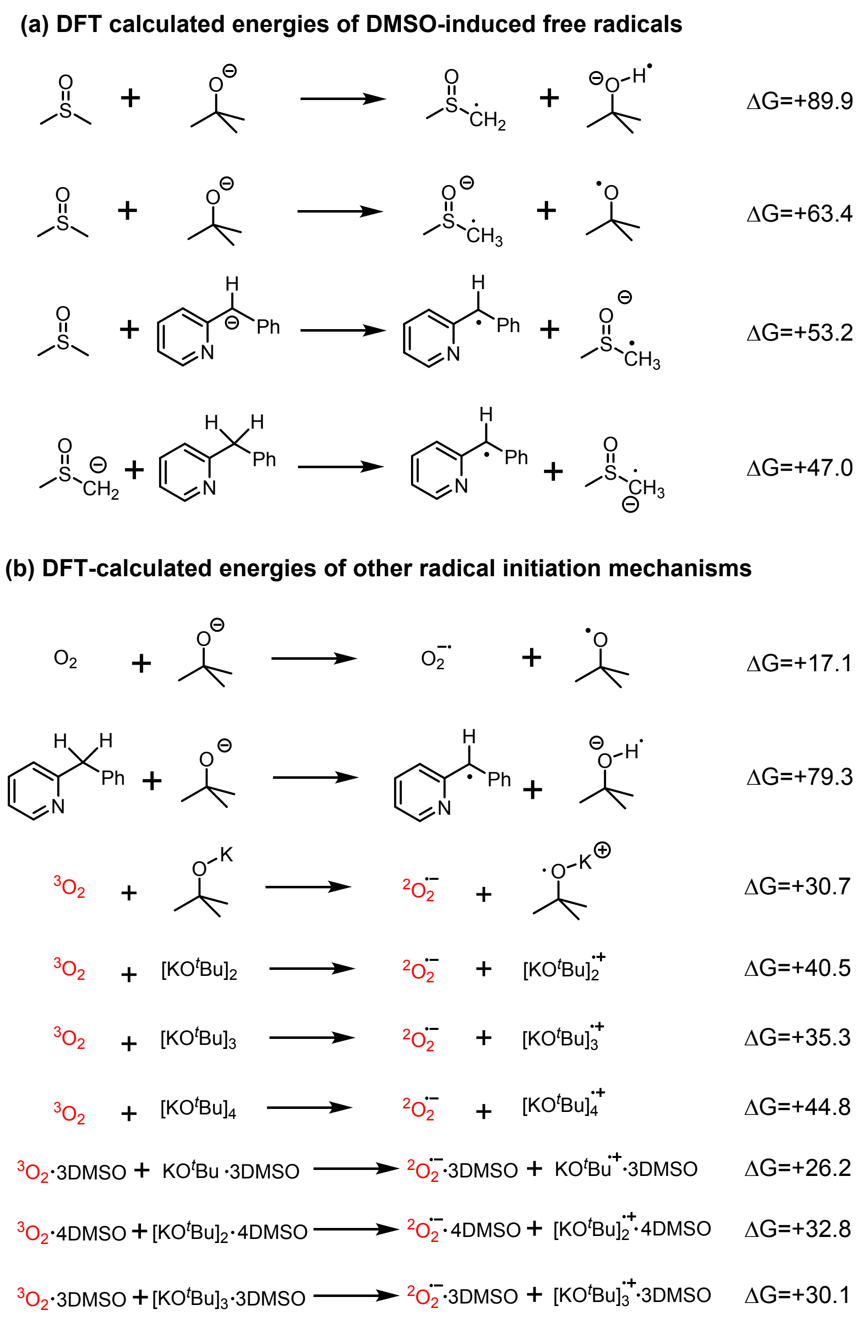

Figure S9. Possible radical initiation: (a) DFT-calculated energetics of DMSO-induced free radicals. (b) DFT-calculated energetics of other radical initiation mechanisms. Gibbs free energies including DMSO solvation are shown in kcal/mol.

It was observed that as the KO^{t}Bu aggregates increase in size, the thermodynamic energy for single electron transfer with O_2 increases significantly (from +30.7 to +44.8 kcal/mol), with the coordination of explicit DMSO solvent providing only limited facilitation, and the process remains unfavorable. In addition, single electron transfer between the $^{-}O^t$Bu anion and O_2 was found to be possible (+17.1 kcal/mol); however, this thermodynamic energy is still substantially higher than that of the substrate-

involved radical generation pathway (-6.1 kcal/mol) discussed in this work, further confirming that the radical initiation process in the reaction is primarily dominated by the reaction of the substrate–KO^{t}Bu cluster with O_2.

5. Computed Energies of Optimized Structures (energies in Hartree)

Structure	E	H_corr	G_corr	E_solv
**1**	-518.382335	0.211845	0.163265	-518.512598
**$^{-}$O^{t}Bu**	-232.928928	0.127937	0.092924	-233.111069
**TS1**	-751.349143	0.338191	0.268727	-751.617927
**HO^{t}Bu**	-233.5681	0.145025	0.108676	-233.638731
**2**	-517.781787	0.196596	0.157772	-517.99644
**O_2**	-150.27428	0.007242	-0.016028	-150.337035
**$O_2^{\cdot-}$**	-150.252936	0.006234	-0.016863	-150.45664
**3**	-668.064988	0.205195	0.149990	-668.333594
**4**	-668.080391	0.207457	0.156661	-668.367184
**DMSO**	-553.11053	0.087035	0.052288	-553.198258
**TS2**	-1221.177043	0.295426	0.220975	-1221.538513
**DMSOO**	-628.307386	0.093401	0.057072	-628.420452
**5**	-592.972217	0.201775	0.155481	-593.223471
**TS3**	-1111.379549	0.410505	0.334036	-1111.733621
**6**	-593.581331	0.217604	0.159564	-593.741368
**$2^{\cdot}$**	-517.745598	0.198264	0.169718	-517.704061
**TS4**	-1186.455925	0.415716	0.332465	-1186.875533
**7**	-668.684709	0.222169	0.166103	-668.872818
**TS5**	-1221.757747	0.308312	0.230823	-1222.027433
**TS1-1K**	-779.664737	0.342494	0.271938	-779.878097
**KO^{t}Bu**	-261.25478	0.132875	0.091702	-261.359959
**2-1K**	-696.363428	0.209048	0.150911	-696.577623
**^{3}[3-1K]**	-696.363428	0.209048	0.1479	-696.577623
**4-1K**	-696.391085	0.211717	0.157348	-696.615103
**5-1K**	-621.283191	0.206354	0.162768	-621.475514
**TS2-1K**	-1249.515365	0.299129	0.229305	-1249.802536
**TS3-1K**	-1139.680727	0.41473	0.336348	-1139.991828
**^{2}2-1K**	-545.902535	0.201385	0.149243	-546.103508
**^{3}6-1K**	-696.348381	0.209269	0.147786	-696.570478
**[KO^{t}Bu]$_2$**	-522.605642	0.269751	0.198454	-522.769927
**4-2K**	-957.746024	0.347588	0.26607	-958.02999
**^{3}3-2K**	-957.702187	0.344689	0.256270	-957.983918
**^{3}6-2K**	-957.683394	0.344988	0.250819	-957.972171
**TS2-2K**	-1510.812315	0.435118	0.342401	-1511.217412
**[KO^{t}Bu]$_3$**	-783.937648	0.404836	0.313243	-784.173553
**2-3K**	-1068.743044	0.472905	0.371285	-1069.041907
**TS1-3K-**	-1302.337929	0.61496	0.499484	-1302.689249
**^{3}3-3K**	-1219.044587	0.481371	0.371186	-1219.390819
**TS2-3K**	-1772.213637	0.570997	0.457757	-1772.630747
**^{3}6-3K**	-1219.020562	0.481703	0.371440	-1219.37945
**4-3K**	-1219.083948	0.483718	0.381080	-1219.440084

**5-3K**	-1143.976015	0.479079	0.382045	-1144.299298
**^{2}2-3K**	-1068.575309	0.474012	0.366412	-1068.910252
**[KO^{t}Bu]$_4$**	-1045.324399	0.541642	0.425896	-1045.612148
**2-4K**	-1330.117956	0.608458	0.481478	-1330.476586
**TS1-4K**	-1563.704213	0.751232	0.607395	-1564.118337
**^{3}3-4K**	-1480.41439	0.617023	0.488828	-1480.825183
**^{3}6-4K**	-1480.395808	0.617206	0.487225	-1480.813884
**TS2-4K**	-2033.562656	0.707012	0.561776	-2034.046626
**4-4K**	-1480.463049	0.619754	0.498771	-1480.872169
**^{2}2-4K**	-1329.940977	0.609546	0.4786576	-1330.342679

6. References

(1) Grimme, S.; Bannwarth, C.; Shushkov, P. A robust and accurate tight-binding quantum chemical method for structures, vibrational frequencies, and noncovalent interactions of large molecular systems parametrized for all spd-block elements (Z= 1–86). *J. Chem. Theory Comput.* **2017**, *13*, 1989-2009.

(2) Pracht, P.; Bohle, F.; Grimme, S. Automated exploration of the low-energy chemical space with fast quantum chemical methods. *Phys. Chem. Chem. Phys.* **2020**, *22*, 7169-7192.

(3) Lu, T. Molclus program, Version 1.13, http://www.keinsci.com/research/molclus.html

(4) (a) Lee, C.; Yang, W.; Parr, R. G. Development of the Colle-Salvetti correlation-energy formula into a functional of the electron density. *Physical Review B* **1988**, *37*, 785-789. (b) Becke, A. D. Density-functional thermochemistry. I. The effect of the exchange-only gradient correction. *J. Chem. Phys* **1992**, *96*, 2155-2160.

(5) Zhao, Y.; Truhlar, D. G. The M06 suite of density functionals for main group thermochemistry, thermochemical kinetics, noncovalent interactions, excited states, and transition elements: two new functionals and systematic testing of four M06-class functionals and 12 other functionals. *Theor. Chem. Acc.* **2008**, *120*, 215-241.

(6) (a) Adamo, C.; Barone, V. Toward reliable density functional methods without adjustable parameters: The PBE0 model. *J. Chem. Phys* **1999**, *110*, 6158-6170. (b) Ernzerhof, M.; Scuseria, G. E. Assessment of the Perdew–Burke–Ernzerhof exchange-correlation functional. *J. Chem. Phys* **1999**, *110*, 5029-5036.

(7) (a) Liu, W.; Hong, G.; Dai, D.; Li, L.; Dolg, M. The Beijing four-component density functional program package (BDF) and its application to EuO, EuS, YbO and YbS. *Theor. Chem. Acc.* **1997**, *96*, 75-83. (b) Liu, W.; Wang, F.; Li, L. The Beijing density functional (BDF) program package: methodologies and applications. *J. Theor. Comput. Chem.* **2003**, *2*, 257-272. (c) Liu, W.; Wang, F.; Li, L. Relativistic density functional theory: The BDF program package. *Recent advances in relativistic molecular theory* **2004**, 257-282. (d) Zhang, Y.; Suo, B.; Wang, Z.; Zhang, N.; Li, Z.; Lei, Y.; Zou, W.; Gao, J.; Peng, D.; Pu, Z. BDF: A relativistic electronic structure program package. *J. Chem. Phys.* **2020**, *152*. 064113.

(8) Barone, V.; Cossi, M. Quantum calculation of molecular energies and energy gradients in solution by a conductor solvent model. *J. Phys. Chem. A* **1998**, *102*, 1995-2001.

(9) Marcus, R. A. Chemical and electrochemical electron-transfer theory. *Annu. Rev. Phys. Chem.* **1964**, *15*, 155-196.

7. Cartesian Coordinates of the Optimized Structure

1

C	-2.61171	1.461267	-0.38238
C	-1.47456	1.078503	0.315367
C	-1.23642	-0.26753	0.599412
C	-2.15726	-1.2182	0.167433
C	-3.29832	-0.83677	-0.53182
C	-3.52866	0.50442	-0.80849
H	-2.78465	2.511549	-0.59529
H	-0.74942	1.818337	0.639373
H	-1.98287	-2.26889	0.382661
H	-4.00699	-1.59074	-0.85984
H	-4.41802	0.804745	-1.35309
C	0.013625	-0.67548	1.353441
H	-0.01802	-1.74856	1.561971
H	0.053451	-0.15104	2.311837
C	1.272306	-0.33694	0.589474
C	1.85655	-1.25465	-0.28372
C	2.988355	-0.88417	-0.9934
H	1.421231	-2.24139	-0.40068
C	2.85106	1.239174	0.075457
C	3.502332	0.393708	-0.81261
H	3.462614	-1.58118	-1.67701
H	3.221875	2.247402	0.245762
H	4.385773	0.729699	-1.34359
N	1.76447	0.892874	0.765566

$^{-}$OtBu

O	0.000091	0.000027	1.47755
C	-0.00001	-8E-06	0.154454
C	1.185534	-0.83979	-0.43479
C	-1.32009	-0.60682	-0.43469
C	0.134494	1.446596	-0.43477
H	2.124533	-0.42297	-0.05216
H	1.103148	-1.86416	-0.05304
H	1.238605	-0.87673	-1.53549
H	-2.16608	-0.02357	-0.05261
H	-1.37876	-0.63397	-1.53538
H	-1.42834	-1.62857	-0.05237
H	0.140465	1.510973	-1.53548
H	-0.69637	2.051124	-0.05259
H	1.062482	1.887751	-0.05257

TS1

C	4.080302	0.441938	-0.0792
C	2.866942	-0.20578	-0.2839
C	1.659505	0.500381	-0.27955
C	1.710832	1.881645	-0.04414
C	2.923993	2.529536	0.152615
C	4.118832	1.815939	0.133341
H	5.003953	-0.13237	-0.08818
H	2.848103	-1.27924	-0.43391
H	0.76364	2.412354	0.015919
H	2.935671	3.601408	0.335806
H	5.06758	2.32203	0.293091
C	0.320993	-0.11113	-0.56851
C	0.069195	-1.52288	-0.27909
C	-0.95271	-2.20745	-0.98064
C	-1.2818	-3.50197	-0.64302
H	-1.47666	-1.69109	-1.77884
C	0.406134	-3.3991	1.019669
C	-0.587	-4.13338	0.390333
H	-2.071	-4.02345	-1.1791
H	0.980505	-3.85086	1.829733
H	-0.80602	-5.15201	0.692643
N	0.736288	-2.14602	0.714074
O	-1.27682	1.62256	0.607099
C	-2.57401	1.682348	0.16543
C	-2.64988	1.781152	-1.37587
C	-3.3675	0.438169	0.620564
C	-3.23505	2.938049	0.766075
H	-2.05276	2.635829	-1.70907
H	-2.22538	0.881092	-1.83113
H	-3.676	1.900523	-1.74863
H	-3.30862	0.359118	1.710241
H	-4.42513	0.466413	0.324153
H	-2.91318	-0.46458	0.200096
H	-4.28617	3.059113	0.470024
H	-3.1787	2.882353	1.857396
H	-2.67484	3.82341	0.449317
H	-0.53	0.61121	0.052447
H	0.032882	0.115615	-1.60205

HO^{t}Bu

O	-0.02193	-0.00021	1.443239

C	0.005147	0.000007	0.015068
C	-0.68244	1.259635	-0.51058
C	1.48388	-0.00168	-0.34867
C	-0.68543	-1.2578	-0.51104
H	-1.7392	1.271795	-0.22161
H	-0.20387	2.14871	-0.09322
H	-0.63486	1.31489	-1.60179
H	1.970436	-0.88745	0.066449
H	1.618846	-0.00156	-1.43332
H	1.972573	0.882699	0.066897
H	-0.63798	-1.31276	-1.60227
H	-0.20895	-2.14815	-0.09401
H	-1.74222	-1.26756	-0.22209
H	-0.94628	0.000094	1.720387

2

C	-2.8921	1.411459	-0.24706
C	-1.58626	0.945774	-0.26373
C	-1.26553	-0.42185	-0.02646
C	-2.39844	-1.26385	0.184516
C	-3.69435	-0.78955	0.197937
C	-3.97179	0.565437	-0.0067
H	-3.07045	2.468169	-0.44122
H	-0.79633	1.640135	-0.51842
H	-2.21704	-2.32332	0.350784
H	-4.51118	-1.48771	0.374631
H	-4.9905	0.941123	0.010569
C	0.031534	-1.00671	-0.03287
H	0.055478	-2.09266	-0.06832
C	1.311063	-0.42831	-7.1E-05
C	1.604781	0.952662	0.263615
C	2.901977	1.406083	0.260755
H	0.802601	1.630091	0.522697
C	3.603737	-0.80934	-0.18123
C	3.959288	0.523558	0.010685
H	3.101217	2.456055	0.4712
H	4.393436	-1.54991	-0.34127
H	4.995894	0.842776	-0.00808
N	2.373338	-1.29099	-0.18698

O2

O	0	0	0.598003
O	0	0	-0.598

$O_2^{\cdot -}$

O	0	0	0.668067
O	0	0	-0.66807

3

C	2.831664	1.082063	0.957572
C	1.536255	0.636494	0.741858
C	1.282601	-0.62632	0.144627
C	2.431077	-1.38291	-0.20991
C	3.717064	-0.92633	0.010877
C	3.94141	0.318615	0.601148
H	2.978319	2.058002	1.417379
H	0.683583	1.237348	1.02548
H	2.283312	-2.35591	-0.67445
H	4.561202	-1.54873	-0.28125
H	4.950936	0.6801	0.776225
C	-0.01337	-1.16055	-0.12767
C	-1.28185	-0.63507	0.16263
C	-2.45734	-1.37643	-0.2066
C	-3.70741	-0.88263	0.047858
H	-2.33122	-2.33729	-0.69827
C	-2.67257	1.022284	1.010894
C	-3.84763	0.366641	0.679731
H	-4.58715	-1.45578	-0.24003
H	-2.72785	1.995438	1.50543
H	-4.81744	0.799147	0.9011
N	-1.44175	0.576501	0.785194
H	-0.01856	-2.1331	-0.61605
O	-0.17359	0.749169	-2.26351
O	-0.00671	1.802092	-1.67275

MECP-0K

C	2.829682	-1.44457	0.19647
C	1.545386	-0.91464	0.263762
C	1.269125	0.344676	-0.29815
C	2.333096	1.043382	-0.89349
C	3.608916	0.507264	-0.96165
C	3.866231	-0.74989	-0.41623
H	3.023066	-2.41994	0.636978
H	0.738634	-1.45117	0.736527
H	2.14149	2.028897	-1.31247
H	4.407338	1.06931	-1.44056
H	4.863928	-1.17838	-0.46837
C	-0.03267	0.980572	-0.17959

C	-1.30453	0.320116	-0.32324
C	-2.46263	1.092426	-0.60008
C	-3.69623	0.491551	-0.65978
H	-2.35656	2.161792	-0.75482
C	-2.61488	-1.56954	-0.17724
C	-3.79307	-0.88942	-0.43886
H	-4.58457	1.081358	-0.87344
H	-2.64242	-2.64623	-0.00225
H	-4.74364	-1.41142	-0.47304
N	-1.40712	-1.01321	-0.12294
H	-0.04748	2.011948	-0.52755
O	-0.15818	1.313445	1.655833
O	0.12812	0.258397	2.377669
**4**			
C	-3.27513	0.273889	-1.12353
C	-2.05405	0.824773	-0.74894
C	-1.29476	0.248011	0.268444
C	-1.78688	-0.89286	0.904017
C	-3.00671	-1.4447	0.530479
C	-3.75829	-0.86379	-0.48643
H	-3.85424	0.738469	-1.9177
H	-1.66636	1.718663	-1.22184
H	-1.18858	-1.35561	1.68004
H	-3.37317	-2.33397	1.037089
H	-4.71323	-1.29369	-0.77737
C	0.031221	0.877412	0.669651
C	1.212251	0.000025	0.245404
C	2.236616	0.580251	-0.51599
C	3.281338	-0.22927	-0.92381
H	2.18443	1.663068	-0.62978
C	2.237979	-2.04585	0.210706
C	3.284866	-1.5787	-0.56378
H	4.098901	0.182357	-1.51096
H	2.209107	-3.08841	0.527423
H	4.086694	-2.2468	-0.8651
N	1.21872	-1.28009	0.617601
H	0.076893	0.949328	1.771742
O	0.107994	2.141919	0.115814
O	1.261723	2.799598	0.732422
**DMSO**			
S	-7.8E-05	0.237051	-0.43898
O	-0.00079	1.494131	0.383689

C	1.348178	-0.81024	0.180037
H	1.314708	-1.78924	-0.30321
H	2.285681	-0.30947	-0.06415
H	1.259758	-0.90702	1.264352
C	-1.34739	-0.81144	0.179996
H	-2.28537	-0.31184	-0.06476
H	-1.31274	-1.79068	-0.30267
H	-1.25923	-0.90752	1.264399
**TS2**			
C	3.039441	2.735408	1.075402
C	1.919968	1.921073	0.939284
C	1.822845	1.011956	-0.11168
C	2.871064	0.938543	-1.0314
C	3.98994	1.752108	-0.89699
C	4.080618	2.655478	0.157842
H	3.097318	3.439405	1.901802
H	1.086578	1.971386	1.630191
H	2.805961	0.220884	-1.84148
H	4.796609	1.681905	-1.62229
H	4.954612	3.293159	0.260434
C	0.561049	0.146786	-0.2371
C	0.908579	-1.33837	-0.07366
C	0.361009	-2.0444	0.998935
C	0.679983	-3.38177	1.148391
H	-0.32467	-1.50989	1.642671
C	2.00874	-3.20265	-0.81803
C	1.530233	-3.98255	0.222964
H	0.265056	-3.95928	1.97041
H	2.665179	-3.63729	-1.57149
H	1.80543	-5.02993	0.297662
N	1.71016	-1.91178	-0.9752
O	-0.38031	0.55446	0.670663
O	-1.86356	-0.21276	0.130553
S	-3.57416	0.247826	-0.26667
O	-5.01426	0.057369	-0.73019
C	-3.0015	1.662322	-1.25186
H	-2.09002	2.062792	-0.81074
H	-2.79059	1.284218	-2.25362
H	-3.8052	2.399932	-1.3079
C	-3.76383	1.119876	1.316874
H	-4.05854	0.372967	2.055689
H	-2.81552	1.568712	1.60611
H	-4.56339	1.856824	1.212731

H	0.202864	0.246002	-1.27917

DMSOO

S	0	0.192037	0.000001
O	-2E-06	0.913651	-1.27006
C	-1.40437	-0.91735	-2E-06
H	-1.38521	-1.53012	0.901197
H	-2.29009	-0.2812	0.000014
H	-1.38521	-1.53009	-0.90122
C	1.404376	-0.91734	-2E-06
H	2.290093	-0.2812	0.000014
H	1.385209	-1.53011	0.901196
H	1.385218	-1.53009	-0.90122
O	-2E-06	0.913645	1.270068

5

C	2.698777	-1.63793	-0.0671
C	1.536094	-1.08554	-0.59587
C	1.23478	0.26273	-0.3966
C	2.123256	1.043787	0.334916
C	3.286421	0.497254	0.866086
C	3.58105	-0.84899	0.667695
H	2.919972	-2.69121	-0.22817
H	0.838153	-1.69946	-1.15688
H	1.834726	2.087201	0.43596
H	3.97227	1.121709	1.435807
H	4.491045	-1.28072	1.078231
C	-0.0241	0.992373	-0.96518
C	-1.27031	0.270464	-0.38603
C	-2.06455	1.010212	0.494863
C	-3.19682	0.43173	1.035338
H	-1.71886	2.023918	0.672543
C	-2.68249	-1.5256	-0.22069
C	-3.52263	-0.87504	0.671077
H	-3.82976	0.983041	1.727926
H	-2.90924	-2.54511	-0.53567
H	-4.40543	-1.37402	1.060063
N	-1.58185	-0.98171	-0.7457
O	-0.03476	2.294744	-0.73129
H	-0.03868	0.675951	-2.05071

TS3

C	1.565886	2.744239	1.522776
C	1.223575	1.435391	1.204481
C	1.799115	0.797486	0.110592
C	2.728162	1.491933	-0.66391
C	3.07159	2.801921	-0.34844
C	2.491101	3.434684	0.747245
H	1.093623	3.234622	2.369687
H	0.481261	0.875554	1.762291
H	3.183074	0.990361	-1.51184
H	3.793522	3.334149	-0.9632
H	2.752279	4.461856	0.988072
C	1.333819	-0.63531	-0.22579
C	2.517854	-1.59762	-0.10199
C	2.508233	-2.53984	0.928502
C	3.577009	-3.40972	1.053428
H	1.639487	-2.54622	1.575164
C	4.539831	-2.36253	-0.8554
C	4.625708	-3.32403	0.141188
H	3.596249	-4.15156	1.847701
H	5.335464	-2.26921	-1.59415
H	5.483643	-3.98681	0.19429
N	3.517984	-1.51582	-0.98678
O	0.287815	-1.04738	0.541566
H	1.098417	-0.61475	-1.31477
C	-4.82907	-1.62409	1.156624
C	-3.77239	-0.79568	0.799855
C	-3.02065	-1.03657	-0.36234
C	-3.38166	-2.15786	-1.1275
C	-4.43148	-2.99007	-0.76542
C	-5.17176	-2.72829	0.382901
H	-5.3916	-1.40359	2.060964
H	-3.51719	0.064039	1.404145
H	-2.81451	-2.37703	-2.02886
H	-4.67585	-3.8472	-1.38842
H	-5.99691	-3.37396	0.670252
C	-1.84094	-0.23833	-0.77399
H	-1.55565	-0.4927	-1.79955
H	-0.83066	-0.6643	-0.08713
C	-1.76812	1.220766	-0.59167
C	-0.94709	1.976974	-1.45854
C	-0.7628	3.323831	-1.24394
H	-0.43915	1.473165	-2.27415
C	-2.20356	3.128508	0.628481
C	-1.41094	3.933943	-0.16893
H	-0.10879	3.896754	-1.89483
H	-2.7277	3.557383	1.483067

H	-1.29112	4.989753	0.048692
N	-2.38356	1.82013	0.442586

TS4

C	2.128841	2.775984	1.850487
C	1.571408	1.623871	1.306471
C	2.279072	0.867833	0.372286
C	3.549368	1.297933	-0.01577
C	4.103587	2.450168	0.528673
C	3.397402	3.194099	1.467813
H	1.561295	3.352803	2.575336
H	0.57616	1.302111	1.585193
H	4.099166	0.713432	-0.74468
H	5.094204	2.768727	0.215176
H	3.830855	4.095209	1.892895
C	1.636473	-0.37582	-0.23731
C	2.551512	-1.59623	-0.20037
C	2.254129	-2.65704	0.659771
C	3.106385	-3.74523	0.689108
H	1.340015	-2.6054	1.234969
C	4.425791	-2.66372	-0.9698
C	4.226296	-3.75379	-0.14001
H	2.898735	-4.58778	1.3429
H	5.280772	-2.63438	-1.64391
H	4.919583	-4.58872	-0.15154
N	3.610479	-1.60649	-1.01172
O	0.450447	-0.62656	0.449552
O	-0.24919	-1.6552	-0.28881
H	1.414429	-0.17574	-1.2968
C	-4.98866	-0.71774	1.77208
C	-3.60234	-0.68869	-0.22835
C	-4.50002	-1.61105	-0.78884
C	-5.60845	-2.07765	-0.09498
C	-5.86667	-1.63079	1.195958
H	-5.16974	-0.35903	2.7826
H	-4.31441	-1.96882	-1.79873
H	-6.27725	-2.79185	-0.5695
H	-6.73395	-1.98864	1.743814
C	-2.39504	-0.29409	-0.99539
H	-2.4738	-0.65572	-2.02607
C	-1.90481	1.094794	-1.01066
C	-1.05973	1.501109	-2.06981
C	-0.53218	2.773175	-2.08772
H	-0.82796	0.7914	-2.85732
C	-1.68397	3.182614	-0.05709
C	-0.85006	3.656225	-1.05452
H	0.123534	3.082643	-2.89737
H	-1.9593	3.833442	0.773531
H	-0.45656	4.666195	-1.02069
H	-1.38286	-0.97575	-0.58609
C	-3.87573	-0.25769	1.08064
H	-3.20709	0.459373	1.53651
N	-2.19928	1.953745	-0.01859

7

C	3.233973	0.102609	1.144
C	2.046105	0.728234	0.779761
C	1.291713	0.228858	-0.27869
C	1.729607	-0.90633	-0.95966
C	2.91236	-1.5312	-0.58947
C	3.670292	-1.02637	0.462491
H	3.818922	0.50129	1.966737
H	1.70799	1.609058	1.312323
H	1.123983	-1.3046	-1.767
H	3.244698	-2.41438	-1.12552
H	4.597099	-1.51257	0.749429
C	-0.02178	0.864599	-0.69161
C	-1.20188	0.036203	-0.2162
C	-1.85541	0.323772	0.977684
C	-2.8947	-0.50373	1.373457
H	-1.553	1.185955	1.558965
C	-2.52188	-1.78359	-0.60324
C	-3.23745	-1.58481	0.569842
H	-3.4318	-0.30861	2.296182
H	-2.76237	-2.61514	-1.26146
H	-4.04331	-2.2576	0.840674
N	-1.52517	-0.99174	-0.99936
H	-0.07548	0.920868	-1.7852
O	-0.03749	2.177007	-0.1477
O	-1.21609	2.825867	-0.64021
H	-0.82777	3.42534	-1.29661

TS5

C	4.508833	-1.21594	-0.77209
C	3.119712	-1.25728	-0.84586
C	2.349069	-0.64574	0.13634
C	2.980788	0.008464	1.194139
C	4.366411	0.049694	1.267491

C	5.13587	-0.56329	0.282148
H	5.103466	-1.69787	-1.54233
H	2.610291	-1.76658	-1.6556
H	2.375431	0.49087	1.956702
H	4.848962	0.56082	2.095014
H	6.219453	-0.53232	0.339031
C	0.822756	-0.68123	0.056656
C	0.29829	0.745451	-0.06424
C	0.31257	1.37267	-1.31263
C	-0.15401	2.66926	-1.41613
H	0.676042	0.809493	-2.16353
C	-0.62549	2.599709	0.919884
C	-0.63149	3.307388	-0.27027
H	-0.15118	3.183673	-2.37218
H	-1.0039	3.053439	1.833285
H	-1.00428	4.32507	-0.30239
N	-0.18108	1.339694	1.03069
O	0.397651	-1.454	-1.00065
O	-1.51278	-1.3095	-0.68648
H	0.459152	-1.04936	1.035438
S	-3.2363	-1.05014	0.080868
O	-4.53433	-1.76756	0.030167
C	-3.40661	0.588349	-0.61718
H	-2.47198	1.130994	-0.48322
H	-3.61966	0.457794	-1.67796
H	-4.24057	1.085156	-0.12013
C	-2.78391	-0.68783	1.780104
H	-2.6068	-1.64264	2.276733
H	-1.87671	-0.07527	1.779276
H	-3.62449	-0.17651	2.252429
H	-1.20597	-2.22193	-0.53372

6

C	2.89059	0.182439	1.402369
C	1.727876	0.751381	0.90167
C	1.254385	0.389628	-0.35733
C	1.967869	-0.53571	-1.11309
C	3.131109	-1.11124	-0.61115
C	3.59321	-0.75454	0.649402
H	3.254212	0.472518	2.383116
H	1.18001	1.497096	1.466614
H	1.617934	-0.80084	-2.1076
H	3.681193	-1.82952	-1.21073
H	4.503333	-1.19701	1.041361
C	-0.04445	0.978978	-0.86956
C	-1.25403	0.193617	-0.35368
C	-1.33063	-1.19715	-0.39879
C	-2.4827	-1.81188	0.062545
H	-0.49413	-1.77525	-0.77322
C	-3.35079	0.3492	0.565515
C	-3.51949	-1.02583	0.557317
H	-2.57207	-2.89324	0.043268
H	-4.13027	1.004503	0.944973
H	-4.43572	-1.4682	0.930907
N	-2.24474	0.946291	0.117098
O	-0.15778	2.325481	-0.50007
H	-0.05282	0.884265	-1.96869
H	-1.07397	2.424371	-0.18049

2·

C	2.817544	1.451875	-0.00041
C	1.521406	0.965762	-0.00018
C	1.277942	-0.42789	-7.4E-05
C	2.401202	-1.28917	0.000298
C	3.69102	-0.79274	0.000292
C	3.908623	0.584446	-0.00021
H	2.982421	2.524821	-0.00082
H	0.672874	1.635368	0.000003
H	2.238273	-2.36325	0.000685
H	4.533115	-1.47739	0.00055
H	4.920132	0.977308	-0.00023
C	-0.01828	-1.02885	0.000039
H	-0.02126	-2.11596	0.000338
C	-1.32067	-0.43456	0.00001
C	-2.45324	-1.28128	-0.00038
C	-3.71986	-0.73214	-0.00033
H	-2.31509	-2.35726	-0.00057
C	-2.68733	1.418458	0.000371
C	-3.85169	0.653768	0.00006
H	-4.59615	-1.37243	-0.00058
H	-2.75135	2.504764	0.000819
H	-4.82392	1.132768	0.000126
N	-1.46272	0.912167	0.00038

TS1-1K

C	-3.78566	0.926897	0.084694
C	-2.46584	1.177971	-0.28167
C	-1.65832	0.171217	-0.83398

C	-2.24422	-1.09406	-1.01875
C	-3.56469	-1.34104	-0.66581
C	-4.34494	-0.33363	-0.10148
H	-4.38552	1.729932	0.504348
H	-2.04761	2.166842	-0.12965
H	-1.62172	-1.89491	-1.40501
H	-3.98584	-2.32997	-0.82321
H	-5.37601	-0.52594	0.178057
C	-0.21329	0.337619	-1.16923
C	0.53005	1.481019	-0.64103
C	1.466608	2.17792	-1.43143
C	2.246377	3.170319	-0.87435
H	1.560259	1.921539	-2.48121
C	1.147853	2.764333	1.188993
C	2.086281	3.487779	0.474915
H	2.96833	3.705245	-1.48445
H	0.990742	2.976493	2.246103
H	2.668866	4.265914	0.953916
N	0.397386	1.785426	0.674282
K	-0.66146	-0.52165	1.908248
O	0.653187	-1.75108	0.151295
C	1.95392	-2.19871	-0.089
C	2.124907	-2.535	-1.57788
C	2.975087	-1.12142	0.313378
C	2.185524	-3.46056	0.749216
H	1.379088	-3.27862	-1.87258
H	1.966813	-1.63939	-2.18587
H	3.12163	-2.92996	-1.80184
H	2.861465	-0.87814	1.376201
H	4.008265	-1.44637	0.150223
H	2.808857	-0.20485	-0.25962
H	3.188673	-3.87628	0.605044
H	2.061081	-3.22847	1.812963
H	1.448284	-4.22197	0.479765
H	0.344644	-0.74488	-0.57205
H	-0.03776	0.1917	-2.23786

KO^{t}Bu

K	-2.5252	-0.00018	-0.03258
O	-0.24353	-0.00155	-0.41593
C	1.058197	-6E-06	-0.02103
C	1.769094	1.285328	-0.49928
C	1.158823	-0.06378	1.522051
C	1.804212	-1.21996	-0.6051

H	1.709781	1.343532	-1.5905
H	1.249389	2.15804	-0.08884
H	2.824241	1.337349	-0.20306
H	0.660536	-0.97389	1.876766
H	2.188082	-0.06604	1.901201
H	0.637225	0.799927	1.951814
H	2.860837	-1.26663	-0.31325
H	1.309928	-2.13836	-0.27021
H	1.74498	-1.18768	-1.69738

2-1K

C	2.768895	0.704587	1.293591
C	1.473978	0.282142	1.00681
C	1.219046	-0.75853	0.069476
C	2.373564	-1.34904	-0.51537
C	3.653631	-0.91864	-0.22123
C	3.874763	0.128089	0.678509
H	2.910867	1.4935	2.02893
H	0.630635	0.727775	1.522217
H	2.232841	-2.16682	-1.21814
H	4.499287	-1.40673	-0.69914
H	4.880741	0.463208	0.907444
C	-0.08371	-1.21036	-0.33323
C	-1.35328	-0.69234	-0.00166
C	-2.53076	-1.4781	-0.24013
C	-3.77158	-0.9851	0.050047
H	-2.4108	-2.48093	-0.63793
C	-2.74085	1.025577	0.774062
C	-3.90347	0.311093	0.589507
H	-4.65267	-1.59757	-0.12279
H	-2.78813	2.037836	1.178376
H	-4.86761	0.730268	0.850935
N	-1.50676	0.58675	0.482891
K	0.22773	1.573622	-1.32821
H	-0.0961	-2.16292	-0.85643

^{3}3-1K

C	2.49881	-2.04171	0.239038
C	1.235478	-1.5995	-0.12889
C	1.063345	-0.3955	-0.85807
C	2.236908	0.317464	-1.19855
C	3.494188	-0.13732	-0.83099
C	3.639752	-1.3148	-0.10144
H	2.596124	-2.97905	0.780358

H	0.360534	-2.1853	0.126028
H	2.135561	1.249836	-1.74238
H	4.370965	0.439748	-1.1096
H	4.62471	-1.66882	0.184921
C	-0.21345	0.14561	-1.23702
C	-1.49477	-0.17111	-0.70593
C	-2.66647	0.217358	-1.41106
C	-3.9087	-0.07473	-0.904
H	-2.55853	0.733424	-2.35886
C	-2.83302	-1.0717	0.967207
C	-4.01174	-0.75492	0.317217
H	-4.80228	0.213242	-1.44984
H	-2.86624	-1.58038	1.93053
H	-4.97094	-1.0169	0.747629
N	-1.61048	-0.78773	0.507479
K	0.414862	0.59932	1.828516
O	0.089673	2.501334	-0.17017
O	1.218962	2.671309	0.394168
H	-0.20991	0.825152	-2.08005

4-1K

C	1.890531	-2.03474	1.170988
C	0.78577	-1.36685	0.640429
C	0.899556	-0.6103	-0.52766
C	2.144138	-0.5549	-1.16993
C	3.243908	-1.21826	-0.64005
C	3.123715	-1.95639	0.535706
H	1.777326	-2.62867	2.073291
H	-0.16739	-1.43739	1.150777
H	2.228025	0.055195	-2.05965
H	4.201844	-1.1609	-1.14831
H	3.983311	-2.47983	0.943803
C	-0.23264	0.237569	-1.11141
C	-1.58559	-0.0699	-0.50945
C	-2.57553	-0.72369	-1.23773
C	-3.80774	-0.96358	-0.6457
H	-2.37531	-1.02995	-2.25856
C	-2.96989	0.105981	1.310278
C	-4.0155	-0.53752	0.659151
H	-4.59472	-1.46932	-1.1963
H	-3.09578	0.450664	2.334799
H	-4.96156	-0.69632	1.164631
N	-1.78121	0.337611	0.754617
K	1.288768	1.94139	1.193553
O	-0.05352	1.61929	-0.88824
O	1.28346	2.038054	-1.31381
H	-0.25781	0.04347	-2.1957

5-1K

C	-2.26377	-2.07459	-0.85309
C	-1.12664	-1.62314	-0.19065
C	-1.16379	-0.46129	0.581904
C	-2.37343	0.22561	0.687926
C	-3.51589	-0.2236	0.031771
C	-3.46292	-1.37457	-0.7485
H	-2.21442	-2.9762	-1.45716
H	-0.19643	-2.1772	-0.28292
H	-2.37222	1.112054	1.317189
H	-4.45251	0.317855	0.137572
H	-4.34965	-1.72924	-1.26518
C	0.053305	0.124584	1.348235
C	1.339461	-0.19883	0.563577
C	2.319506	-1.04297	1.086035
C	3.485834	-1.2764	0.372899
H	2.154323	-1.50395	2.05401
C	2.631267	0.168352	-1.30971
C	3.651498	-0.65841	-0.86032
H	4.255313	-1.93087	0.771724
H	2.723104	0.668943	-2.27273
H	4.543124	-0.80734	-1.45891
N	1.507166	0.398935	-0.63031
K	-0.40407	2.405022	-0.6564
O	-0.06644	1.441943	1.601182
H	0.121445	-0.5061	2.270157

TS2-1K

C	-4.07079	-1.26561	1.273492
C	-2.85465	-0.68401	0.942856
C	-2.24906	-0.94939	-0.28647
C	-2.88593	-1.8102	-1.17543
C	-4.10605	-2.39534	-0.8488
C	-4.70183	-2.12476	0.376877
H	-4.53318	-1.04651	2.231506
H	-2.36665	-0.00288	1.633137
H	-2.41717	-2.02915	-2.13082
H	-4.59025	-3.06484	-1.55337
H	-5.65376	-2.57893	0.633903
C	-0.88897	-0.34433	-0.61888

C	-0.98283	1.181754	-0.52112
C	-1.48971	1.908302	-1.60131
C	-1.63132	3.280468	-1.50138
H	-1.76722	1.381791	-2.50857
C	-0.77028	3.110604	0.707697
C	-1.26348	3.905274	-0.31332
H	-2.02058	3.858383	-2.33392
H	-0.47308	3.56396	1.652161
H	-1.35335	4.977663	-0.18183
N	-0.62627	1.782844	0.620814
O	0.052582	-0.90867	0.206196
O	1.586533	-0.35677	-0.50272
H	-0.66927	-0.56804	-1.67688
K	1.300882	0.275014	2.064129
S	3.369483	-0.53911	-0.41378
O	3.659301	-0.36187	1.068231
C	5.052188	-0.2146	-1.12532
H	5.030765	-0.39773	-2.20255
H	5.270632	0.836991	-0.93426
H	5.806766	-0.83866	-0.63993
C	3.358457	-2.30352	-0.75085
H	2.450606	-2.6883	-0.28803
H	3.307792	-2.45851	-1.83035
H	4.251475	-2.77402	-0.33584

TS3-1K

C	2.189072	2.595598	0.45885
C	1.890625	1.253439	0.271126
C	1.826584	0.709749	-1.01235
C	2.051398	1.544398	-2.1035
C	2.343366	2.893127	-1.92154
C	2.417903	3.420459	-0.6386
H	2.227818	3.003499	1.463311
H	1.71204	0.615252	1.130042
H	1.994349	1.132545	-3.10759
H	2.515194	3.530269	-2.78382
H	2.647948	4.471275	-0.49236
C	1.480105	-0.76526	-1.2295
C	2.38179	-1.61959	-0.33622
C	3.685577	-1.90871	-0.74732
C	4.517743	-2.64729	0.07442
H	4.029545	-1.54397	-1.7096
C	2.724816	-2.75388	1.631921
C	4.027973	-3.08515	1.301384
H	5.53204	-2.88177	-0.23323
H	2.308073	-3.07764	2.584277
H	4.637108	-3.6684	1.982576
N	1.910019	-2.04187	0.843998
O	0.149199	-1.0518	-1.02031
H	1.78425	-1.00188	-2.2671
K	-0.90245	-1.55963	1.253008
C	-4.60606	-1.45019	0.44887
C	-3.71004	-0.39576	0.285268
C	-2.82834	-0.34423	-0.8094
C	-2.9172	-1.39478	-1.74312
C	-3.81728	-2.43798	-1.58609
C	-4.66516	-2.48358	-0.47995
H	-5.27803	-1.44943	1.303414
H	-3.68825	0.40292	1.018068
H	-2.23192	-1.39268	-2.58494
H	-3.85207	-3.23058	-2.32833
H	-5.36765	-3.30143	-0.35466
C	-1.78326	0.691541	-1.00643
H	-1.84884	1.140393	-1.99967
H	-0.62202	-0.13687	-1.08587
C	-1.50683	1.676907	0.030566
C	-1.2931	3.036037	-0.28553
C	-0.9587	3.936768	0.70074
H	-1.39863	3.355622	-1.31633
C	-1.05096	2.148724	2.256645
C	-0.83896	3.494312	2.020165
H	-0.79625	4.981819	0.45323
H	-0.9595	1.756065	3.269333
H	-0.58859	4.168288	2.831335
N	-1.36289	1.254162	1.313252

MECP'-1K

C	-2.62407	0.051623	1.318876
C	-1.33642	0.376515	0.887989
C	-1.06123	0.554462	-0.48965
C	-2.15342	0.484445	-1.38423
C	-3.42626	0.153867	-0.948
C	-3.66979	-0.08324	0.4112
H	-2.81195	-0.07488	2.382
H	-0.52862	0.484756	1.598301
H	-1.97667	0.678385	-2.43922
H	-4.24096	0.088514	-1.66353

H	-4.66934	-0.33212	0.753707
C	0.262661	0.892951	-0.92638
C	1.471831	0.245269	-0.47336
C	2.720793	0.836671	-0.78298
C	3.887471	0.254379	-0.35366
H	2.72561	1.771703	-1.33038
C	2.5792	-1.45155	0.655065
C	3.828021	-0.92608	0.399961
H	4.844637	0.714702	-0.57983
H	2.489108	-2.37078	1.234616
H	4.72144	-1.41282	0.773096
N	1.424827	-0.91603	0.226961
K	-0.89878	-2.34686	-0.09878
H	0.340639	1.391517	-1.88723
O	-0.14564	2.626429	1.25605
O	0.562698	2.589535	0.188646

MECP-1K

C	2.534456	-1.92916	-0.30553
C	1.265216	-1.41623	-0.54862
C	1.078446	-0.0639	-0.9164
C	2.239677	0.727645	-1.04937
C	3.50287	0.207951	-0.81102
C	3.664572	-1.12224	-0.42618
H	2.642833	-2.97818	-0.04265
H	0.397718	-2.06122	-0.46944
H	2.122646	1.771901	-1.31733
H	4.372535	0.849537	-0.91943
H	4.654254	-1.52731	-0.24156
C	-0.21892	0.540338	-1.14897
C	-1.47306	0.029797	-0.71054
C	-2.66834	0.43103	-1.35997
C	-3.87086	-0.0245	-0.92378
H	-2.60178	1.094832	-2.21482
C	-2.72483	-1.20984	0.779758
C	-3.91891	-0.88802	0.175321
H	-4.78366	0.274823	-1.42945
H	-2.72072	-1.86273	1.651202
H	-4.85441	-1.28517	0.550322
N	-1.53924	-0.7736	0.390797
K	0.573412	0.00586	2.030284
O	-0.09866	2.347123	0.017403
O	0.678623	2.413583	1.083946
H	-0.26144	1.2607	-1.95653

[2]2-1K

C	2.59928	-0.24241	-1.45246
C	1.317047	0.172384	-1.11831
C	1.090417	0.970745	0.028852
C	2.213681	1.363098	0.789413
C	3.489517	0.931917	0.457328
C	3.689776	0.120195	-0.65901
H	2.756356	-0.82667	-2.35401
H	0.478928	-0.08441	-1.75606
H	2.068039	2.006715	1.651988
H	4.335893	1.241568	1.061335
H	4.690543	-0.2001	-0.9281
C	-0.2207	1.416063	0.422579
C	-1.4699	0.767864	0.143162
C	-2.66848	1.504421	0.224179
C	-3.87235	0.890379	-0.06657
H	-2.62963	2.554124	0.493354
C	-2.65354	-1.12648	-0.44574
C	-3.87133	-0.45523	-0.42294
H	-4.79994	1.450834	-0.02176
H	-2.62874	-2.18464	-0.69837
H	-4.78796	-0.97927	-0.66573
N	-1.48032	-0.55919	-0.1644
K	0.699539	-2.06749	0.84079
H	-0.27307	2.340743	0.990242

[3]6-1K

C	-2.8179	0.336889	1.303413
C	-1.53038	0.286204	0.77632
C	-1.30243	0.208904	-0.62649
C	-2.47082	0.219251	-1.4372
C	-3.74293	0.267293	-0.89942
C	-3.94049	0.312235	0.483755
H	-2.93854	0.413229	2.381751
H	-0.67187	0.342692	1.43529
H	-2.34772	0.188805	-2.51715
H	-4.60058	0.272198	-1.56729
H	-4.94034	0.350669	0.902738
C	-0.01388	0.099137	-1.25295
C	1.258666	-0.05892	-0.66419
C	2.440704	0.190509	-1.43781
C	3.681327	0.04569	-0.88386
H	2.325986	0.522003	-2.46498

C	2.637413	-0.61001	1.143642
C	3.80625	-0.35719	0.461714
H	4.567921	0.255062	-1.47651
H	2.678294	-0.93681	2.183573
H	4.770942	-0.46336	0.943042
N	1.402469	-0.50013	0.629832
K	-0.46536	-2.40593	0.298074
H	-0.00637	0.299906	-2.32091
O	0.852234	2.48024	1.237146
O	0.66697	2.810901	0.091183

[3]3-2K

C	3.448193	-1.69117	-1.60696
C	2.866896	-0.43686	-1.46577
C	2.931675	0.255953	-0.23999
C	3.609982	-0.35899	0.828579
C	4.186766	-1.61285	0.682921
C	4.10689	-2.28985	-0.53262
H	3.403062	-2.19779	-2.56678
H	2.359508	0.024836	-2.30429
H	3.631078	0.14934	1.785951
H	4.696008	-2.06975	1.525927
H	4.565427	-3.26692	-0.64747
C	2.323223	1.5532	-0.03613
C	1.076599	1.983164	-0.57157
C	0.627502	3.310437	-0.36124
C	-0.63042	3.682658	-0.78972
H	1.280337	4.017566	0.139093
C	-0.94147	1.450486	-1.58188
C	-1.44702	2.737489	-1.41771
H	-0.97944	4.700466	-0.64038
H	-1.56031	0.668759	-2.00997
H	-2.44303	2.984548	-1.76707
N	0.271382	1.074542	-1.18402
K	0.283883	-1.57099	0.021353
O	0.987168	0.99972	2.096728
O	0.735926	-0.26711	2.327137
C	-3.10722	-2.19326	-1.60312
C	-3.33059	-1.42607	-0.28041
H	-2.67537	-1.51908	-2.35064
H	-2.39771	-3.01313	-1.43556
H	-4.02515	-2.62521	-2.0178
C	-3.92159	-2.40591	0.754268
C	-4.36934	-0.31248	-0.54624
H	-4.09834	-1.87786	1.698314
H	-4.86511	-2.86363	0.43407
H	-3.19871	-3.20435	0.95334
H	-5.3202	-0.6874	-0.94182
H	-4.58457	0.220758	0.388879
H	-3.9575	0.409481	-1.25893
O	-2.15473	-0.89107	0.173108
K	-1.63523	0.949338	1.793635
H	2.818716	2.231002	0.648309

[3]6-2K

C	2.391516	-2.93966	-0.96831
C	2.279484	-1.62775	-0.51992
C	1.839637	-1.32946	0.792728
C	1.570877	-2.43799	1.627584
C	1.682617	-3.74363	1.174199
C	2.080133	-4.01285	-0.13534
H	2.750001	-3.12511	-1.97807
H	2.539451	-0.80719	-1.17735
H	1.256523	-2.25152	2.651189
H	1.456884	-4.56369	1.850175
H	2.172339	-5.03472	-0.48801
C	1.682778	0.015531	1.303075
C	1.427205	1.175295	0.583496
C	1.49797	2.469887	1.231162
C	1.207815	3.6157	0.543739
H	1.827846	2.510615	2.265057
C	0.78633	2.282474	-1.38279
C	0.815468	3.546247	-0.81552
H	1.296277	4.580308	1.038224
H	0.512195	2.179463	-2.43392
H	0.615808	4.432563	-1.40488
N	1.041541	1.134338	-0.74941
K	-0.61647	-1.21078	-1.10042
C	-4.33561	-1.36778	-1.265
C	-3.92154	-0.46814	-0.07974
H	-4.24081	-0.80365	-2.19834
H	-3.66865	-2.23798	-1.32047
H	-5.36184	-1.74398	-1.18765
C	-4.02337	-1.29336	1.219513
C	-4.93065	0.69911	0.001685
H	-3.73777	-0.66932	2.074223
H	-5.02702	-1.69225	1.406126
H	-3.32179	-2.13394	1.177598

H	-5.97058	0.370071	0.105766
H	-4.69794	1.331191	0.869537
H	-4.84846	1.312787	-0.9012
O	-2.6451	0.004584	-0.25194
K	-1.56494	1.992958	0.768743
H	1.808051	0.12937	2.376584
O	4.072236	1.266602	-1.12781
O	4.38431	0.968002	-0.00028

[KOtBu]$_3$

O	-5.6E-05	2.089919	-0.27076
O	2.334321	-0.85983	0.160761
O	-2.33453	-0.85962	0.160763
K	-2.30467	1.236531	-1.12738
K	2.304856	1.2364	-1.12723
K	-8.5E-05	-0.41462	1.088871
C	0.000082	3.272741	0.43774
C	2.973274	-2.06506	0.029948
C	-2.97341	-2.06489	0.029963
C	-1.24624	4.127771	0.108644
H	-2.15866	3.59884	0.413679
H	-1.25325	5.09485	0.622003
H	-1.28839	4.31731	-0.97055
C	1.243386	4.130259	0.103824
H	1.250164	5.097654	0.616596
H	2.158035	3.603516	0.40598
H	1.281386	4.319104	-0.97562
C	0.003233	3.004359	1.957812
H	0.893594	2.424392	2.227459
H	0.003806	3.919986	2.559084
H	-0.88534	2.423277	2.230961
C	-4.48228	-1.8575	-0.22621
H	-4.62259	-1.29003	-1.15563
H	-5.04181	-2.79548	-0.31983
H	-4.91183	-1.27545	0.595232
C	-2.38235	-2.86545	-1.151
H	-1.31138	-3.02718	-0.98646
H	-2.85926	-3.84142	-1.29923
H	-2.49295	-2.2877	-2.07694
C	-2.81851	-2.90989	1.313646
H	-1.75642	-3.11429	1.49686
H	-3.21015	-2.34656	2.166676
H	-3.33854	-3.87346	1.263717
C	2.383264	-2.865	-1.15196
H	2.860111	-3.84101	-1.30008
H	1.312083	-3.02651	-0.98858
H	2.494956	-2.28691	-2.07756
C	4.482387	-1.85762	-0.2247
H	4.911218	-1.2761	0.597487
H	5.041926	-2.79558	-0.31841
H	4.623596	-1.28958	-1.15364
C	2.817147	-2.91072	1.31306
H	3.20799	-2.34781	2.166734
H	1.754887	-3.11523	1.495178
H	3.337226	-3.87426	1.263143

2-3K

O	-2.522	-0.12529	0.792472
O	0.135452	1.916131	-0.95774
K	2.31379	0.58517	-0.97529
K	-0.62545	1.332289	1.620166
K	-1.7121	0.122255	-1.54844
C	-3.62827	-0.61978	1.436356
C	0.065478	3.208024	-1.43349
C	1.421912	3.657643	-2.0225
H	1.707536	2.9895	-2.84521
H	1.406872	4.67909	-2.41649
H	2.195666	3.616479	-1.24494
C	-0.99642	3.330733	-2.54769
H	-1.98299	3.064266	-2.14951
H	-1.07102	4.340505	-2.96485
H	-0.75009	2.644922	-3.3674
C	-0.30982	4.191298	-0.30455
H	-1.28697	3.919039	0.111442
H	0.439947	4.136993	0.493984
H	-0.36883	5.232224	-0.63981
C	-4.48348	-1.47451	0.474242
H	-5.3793	-1.89446	0.945771
H	-4.80711	-0.85753	-0.3737
H	-3.88198	-2.30438	0.087115
C	-3.20908	-1.50713	2.625913
H	-2.61761	-0.91625	3.335315
H	-4.0602	-1.93527	3.168223
H	-2.57493	-2.32366	2.267739
C	-4.51013	0.531318	1.965313
H	-3.93733	1.135513	2.679725
H	-4.79891	1.181331	1.132451
H	-5.42045	0.185964	2.469364

C	2.859716	0.564214	2.870236
C	4.174575	0.15956	2.674915
C	4.366316	-0.94654	1.838446
C	3.289997	-1.54479	1.220287
C	1.960706	-1.03482	1.410679
N	1.794448	0.033425	2.277746
H	5.36181	-1.36142	1.699605
H	2.652575	1.390384	3.551998
H	4.994154	0.648288	3.186993
H	3.430851	-2.46151	0.662595
C	0.815873	-1.49043	0.736007
H	-0.13942	-1.19847	1.161288
C	0.696286	-2.23996	-0.47904
C	1.726477	-2.54877	-1.40993
C	-0.61741	-2.62542	-0.8841
C	1.453522	-3.15112	-2.63626
H	2.763409	-2.34393	-1.16943
C	-0.87212	-3.23195	-2.09945
H	-1.43434	-2.40241	-0.20077
C	0.160968	-3.4955	-3.00725
H	2.281548	-3.36808	-3.30703
H	-1.89272	-3.52069	-2.34285
H	-0.03811	-3.98421	-3.95468

TS1-3K

O	0.684446	1.997651	1.202644
O	-1.25083	0.776217	-1.56501
O	3.182061	-0.10243	-0.39308
K	1.771965	-0.43104	1.644693
K	-1.83366	2.252458	0.723717
K	1.344893	1.332131	-1.32888
C	1.468925	2.988618	1.758092
C	-2.14596	0.607076	-2.64917
C	4.293573	-0.70767	-0.916
C	1.905554	2.612059	3.192212
H	2.595056	1.758365	3.173927
H	2.425075	3.425533	3.709921
H	1.022898	2.333274	3.777493
C	0.689218	4.319684	1.845647
H	1.291091	5.151527	2.227074
H	0.332252	4.595808	0.845269
H	-0.1773	4.205086	2.510226
C	2.743088	3.228564	0.915635
H	2.474254	3.694857	-0.04296
H	3.460275	3.897194	1.404276
H	3.235155	2.272342	0.701566
C	4.684627	-1.94969	-0.08234
H	3.844238	-2.65214	-0.05856
H	5.564505	-2.47625	-0.47021
H	4.904742	-1.63922	0.946624
C	4.026848	-1.17328	-2.36574
H	3.799755	-0.30296	-2.99463
H	4.87297	-1.70265	-2.81918
H	3.154911	-1.83543	-2.37652
C	5.487186	0.267404	-0.92667
H	5.227784	1.151478	-1.51972
H	5.689023	0.602829	0.095665
H	6.404174	-0.17034	-1.33919
C	-2.27451	-0.87481	-3.01547
H	-2.94454	-1.01349	-3.86936
H	-1.29601	-1.28666	-3.278
H	-2.67134	-1.44636	-2.17273
C	-3.52637	1.164909	-2.27882
H	-3.45103	2.235838	-2.05247
H	-4.24278	1.05075	-3.09789
H	-3.92404	0.646818	-1.40219
C	-1.57338	1.387973	-3.83263
H	-1.43824	2.439932	-3.55937
H	-0.60075	0.974983	-4.12072
H	-2.23113	1.342847	-4.70575
C	-4.91656	0.070732	1.366121
C	-5.69793	-1.01622	1.01658
C	-5.03723	-2.13422	0.501528
C	-3.66565	-2.11548	0.369958
C	-2.93073	-0.96651	0.749842
N	-3.58664	0.120527	1.24199
H	-5.59838	-3.01759	0.209875
H	-5.38568	0.965955	1.773213
H	-6.7737	-0.99206	1.142995
H	-3.1326	-2.97568	-0.01873
C	-1.50443	-0.83823	0.530514
H	-1.07256	-0.14811	1.26419
H	-1.40076	0.004836	-0.73655
C	-0.66779	-2.08288	0.449088
C	-0.6313	-3.01497	1.500415
C	0.18096	-2.30975	-0.63961
C	0.229127	-4.10706	1.472548
H	-1.29667	-2.86892	2.347548

C	1.046633	-3.4008	-0.67455
H	0.162056	-1.60653	-1.46459
C	1.078135	-4.30044	0.383783
H	0.237217	-4.81144	2.299467
H	1.705987	-3.53788	-1.52524
H	1.755683	-5.14806	0.36164

MECP'-3K

O	2.189543	-1.57714	-0.47161
O	1.256803	1.996201	0.193031
K	-1.10838	2.211764	1.196393
K	0.66656	0.084068	-1.63746
K	2.562044	0.043478	1.366491
C	2.6847	-2.7841	-0.90019
C	1.93128	3.131745	-0.21118
C	1.525119	4.351206	0.645232
H	1.67882	4.119772	1.705308
H	2.094691	5.255344	0.406718
H	0.464269	4.5898	0.489485
C	3.458243	2.947138	-0.07469
H	3.7759	2.069367	-0.64914
H	4.029827	3.809321	-0.43327
H	3.725222	2.794228	0.979322
C	1.621532	3.460148	-1.68695
H	1.991156	2.657898	-2.33755
H	0.537806	3.544637	-1.82639
H	2.08324	4.393269	-2.02656
C	3.329945	-3.55016	0.274601
H	3.731348	-4.52862	-0.01209
H	4.155019	-2.9571	0.689275
H	2.584704	-3.70229	1.062941
C	1.554137	-3.65934	-1.48079
H	1.089449	-3.14773	-2.333
H	1.901036	-4.639	-1.82852
H	0.78176	-3.81512	-0.72123
C	3.754917	-2.57374	-1.99058
H	3.311065	-2.04926	-2.84529
H	4.56027	-1.94531	-1.59534
H	4.192503	-3.50997	-2.35595
C	-3.10369	1.692603	-1.70678
C	-3.27388	1.328112	-3.02918
C	-2.92565	0.007838	-3.37288
C	-2.44241	-0.83756	-2.40954
C	-2.27338	-0.38635	-1.05562
N	-2.61475	0.908776	-0.73861
H	-3.05989	-0.34821	-4.39075
H	-3.37488	2.704005	-1.39771
H	-3.67867	2.024068	-3.75351
H	-2.20232	-1.87049	-2.64341
C	-1.71757	-1.26839	-0.1092
H	-1.19127	-2.12479	-0.51938
C	-1.43205	-0.99028	1.278698
C	-2.33134	-0.30465	2.126193
C	-0.24086	-1.4802	1.86371
C	-2.02949	-0.08386	3.467846
H	-3.2819	0.014975	1.71632
C	0.045094	-1.25993	3.204169
H	0.476773	-1.9851	1.216911
C	-0.83586	-0.54428	4.019579
H	-2.75425	0.424956	4.09933
H	0.958364	-1.67102	3.632749
H	-0.61515	-0.38786	5.070601
O	-4.30555	-2.06889	0.771327
O	-3.35812	-2.56316	0.14132

^{3}3-3K

C	2.882779	1.406895	-0.73309
C	1.592175	1.914054	-0.71695
C	1.337964	3.277255	-0.45199
C	2.455904	4.110959	-0.2082
C	3.744359	3.606305	-0.21958
C	3.965761	2.251803	-0.4786
H	3.012458	0.332441	-0.88807
H	0.740606	1.279715	-0.90671
H	2.291879	5.166161	-0.00731
H	4.583462	4.26948	-0.03222
H	4.978412	1.858596	-0.49682
C	0.034861	3.862112	-0.41986
C	-1.2629	3.266104	-0.55083
C	-2.39043	4.120689	-0.53026
C	-3.66087	3.589983	-0.64314
H	-2.24459	5.190955	-0.42776
C	-2.64646	1.4247	-0.78343
C	-3.80028	2.210504	-0.76947
H	-4.53028	4.240387	-0.63392
H	-2.71982	0.330092	-0.81916
H	-4.77329	1.73982	-0.86127

N	-1.41428	1.923525	-0.67595
K	-0.04989	-1.35036	-0.43575
O	0.113417	0.39056	1.997208
O	0.104541	-0.77239	2.667512
C	-3.1437	-2.88304	-1.94981
C	-3.518	-2.42539	-0.52479
H	-3.057	-2.00743	-2.60173
H	-2.17399	-3.39498	-1.93184
H	-3.87512	-3.57055	-2.38924
C	-3.59275	-3.66813	0.383979
C	-4.91465	-1.77066	-0.58162
H	-3.89171	-3.37014	1.396213
H	-4.30369	-4.4225	0.027856
H	-2.60206	-4.12938	0.452344
H	-5.69455	-2.44718	-0.94903
H	-5.20546	-1.43459	0.421611
H	-4.88548	-0.89374	-1.2371
O	-2.58762	-1.534	-0.0428
K	-2.40747	-0.20924	2.094779
C	3.063523	-2.74843	-2.28956
C	3.293231	-2.63051	-0.76859
H	2.014325	-2.99984	-2.48708
H	3.275445	-1.78623	-2.76603
H	3.685837	-3.51671	-2.76259
C	4.788234	-2.32331	-0.5322
C	2.965678	-3.98843	-0.11452
H	4.987373	-2.26385	0.545748
H	5.459034	-3.08082	-0.9532
H	5.037742	-1.3553	-0.97932
H	3.576937	-4.81322	-0.49879
H	3.118651	-3.92128	0.968519
H	1.912044	-4.23755	-0.28515
O	2.508287	-1.63563	-0.23632
K	2.609419	-0.35486	1.936326
H	0.017945	4.93865	-0.26767

TS2-3K

K	-2.92448	0.286982	-1.74083
C	-2.19398	3.891965	-1.64553
C	-0.84307	3.227588	-1.30454
H	-2.57079	3.49597	-2.59707
H	-2.92166	3.654016	-0.86175
H	-2.13181	4.982263	-1.73788
C	-0.30073	3.871313	-0.00874
C	0.152569	3.545963	-2.44312
H	0.638027	3.383917	0.275484
H	-0.11678	4.947403	-0.10629
H	-1.02373	3.733463	0.805112
H	0.257909	4.61913	-2.63941
H	1.145874	3.155296	-2.18801
H	-0.18284	3.057344	-3.36469
O	-0.99215	1.869807	-1.15228
K	0.984948	0.200694	-1.42389
C	-4.61714	-0.95587	2.695579
C	-4.71165	-0.15701	1.379338
H	-4.43392	-2.01217	2.475492
H	-3.7713	-0.58885	3.288255
H	-5.52191	-0.88273	3.310038
C	-4.99467	1.320727	1.721181
C	-5.90545	-0.70237	0.566243
H	-5.03324	1.911078	0.799029
H	-5.93526	1.466656	2.264739
H	-4.1837	1.723809	2.340158
H	-6.85693	-0.65561	1.108255
H	-6.01739	-0.12299	-0.35884
H	-5.71536	-1.74523	0.292405
O	-3.54541	-0.27177	0.658862
K	-1.2127	0.72983	1.319604
C	4.469188	-2.3826	-1.29441
C	4.028961	-1.30815	-0.51946
C	3.152496	-1.50607	0.547308
C	2.74381	-2.81248	0.833181
C	3.189537	-3.88413	0.073915
C	4.050142	-3.67292	-1.00271
H	5.157128	-2.20518	-2.11585
H	4.383191	-0.31063	-0.7526
H	2.047316	-2.96862	1.648389
H	2.866251	-4.89148	0.320113
H	4.401443	-4.51118	-1.59648
C	2.598826	-0.38169	1.430635
C	3.332011	0.940927	1.246612
C	4.147813	1.466296	2.244538
C	4.738349	2.709762	2.058445
H	4.304894	0.904607	3.159148
C	3.675365	2.793427	-0.0702
C	4.49617	3.395711	0.876451
H	5.372003	3.138962	2.828359
H	3.459102	3.298037	-1.00956

H	4.925772	4.372951	0.687441
N	3.106501	1.600865	0.098894
O	1.252908	-0.10107	1.22292
O	0.254648	-1.42863	0.656145
S	-0.93954	-2.56866	-0.45327
O	-1.11717	-1.77786	-1.74918
C	-2.59699	-3.07571	0.021574
H	-2.52375	-3.74262	0.883995
H	-3.1128	-2.13412	0.304868
H	-3.08695	-3.57772	-0.81813
C	-0.2601	-4.15436	-0.97113
H	0.77326	-3.98144	-1.27335
H	-0.27828	-4.84181	-0.12312
H	-0.84695	-4.54633	-1.80468
H	2.781181	-0.71205	2.470909

^{3}6-3K

O	2.10347	-1.56777	-0.78354
O	1.440878	1.856109	0.674978
K	-1.08086	2.117649	0.994666
K	1.377458	0.639522	-1.79063
K	1.9408	-0.60017	1.504087
C	2.586262	-2.70959	-1.37283
C	2.316932	2.891735	0.921102
C	1.588289	4.096288	1.559041
H	1.112581	3.784486	2.497528
H	2.253914	4.935239	1.786868
H	0.815076	4.468643	0.874282
C	3.43683	2.451639	1.888754
H	3.997755	1.617966	1.44941
H	4.152307	3.249548	2.113666
H	2.995746	2.119845	2.836765
C	2.978151	3.3765	-0.38655
H	3.545543	2.555163	-0.84027
H	2.202755	3.699361	-1.09146
H	3.668334	4.213634	-0.23741
C	2.873685	-3.7931	-0.3095
H	3.261193	-4.72616	-0.73419
H	3.615018	-3.41564	0.406293
H	1.953138	-4.02515	0.237382
C	1.558859	-3.27646	-2.37341
H	1.354397	-2.53255	-3.15252
H	1.894921	-4.19731	-2.86414
H	0.616416	-3.47904	-1.85578
C	3.901427	-2.41855	-2.12649
H	3.718594	-1.67574	-2.91285
H	4.6346	-1.99664	-1.43075
H	4.339008	-3.30686	-2.59703
C	-1.983	2.019448	-2.82899
C	-3.23873	2.537212	-2.53531
C	-3.95217	1.896839	-1.51661
C	-3.3881	0.843348	-0.82913
C	-2.05963	0.398031	-1.1395
N	-1.39212	1.02125	-2.17999
H	-4.96909	2.203661	-1.28408
H	-1.40579	2.452715	-3.64699
H	-3.65247	3.36131	-3.10326
H	-3.9851	0.281086	-0.12409
C	-1.34677	-0.58589	-0.42966
H	-0.47201	-0.99259	-0.92714
C	-1.54178	-1.09877	0.892062
C	-2.44905	-0.62703	1.879612
C	-0.66493	-2.13946	1.32409
C	-2.44456	-1.12782	3.180581
H	-3.19348	0.119753	1.630491
C	-0.68055	-2.63497	2.613486
H	0.045369	-2.52512	0.595315
C	-1.56519	-2.12638	3.573281
H	-3.16525	-0.73429	3.893447
H	-0.00295	-3.44454	2.877734
H	-1.58565	-2.52535	4.581526
O	-4.38617	-2.244	-0.04212
O	-3.66648	-2.47627	-0.98399

4-3K

C	-3.64217	-1.35753	-1.00338
C	-2.26408	-1.38236	-0.81736
C	-1.7069	-2.08382	0.254564
C	-2.55241	-2.75441	1.138216
C	-3.93289	-2.72879	0.955465
C	-4.48061	-2.02883	-0.11625
H	-4.0606	-0.80052	-1.83537
H	-1.61708	-0.83367	-1.49165
H	-2.12242	-3.27864	1.985674
H	-4.58052	-3.25674	1.64888
H	-5.5561	-2.00839	-0.26204
C	-0.21892	-2.00321	0.552596
C	0.654149	-2.2032	-0.66955

C	1.201248	-3.45784	-0.93404
C	1.998251	-3.62778	-2.05693
H	1.001402	-4.2854	-0.26175
C	1.643509	-1.32503	-2.5446
C	2.229362	-2.53676	-2.88415
H	2.436787	-4.59532	-2.27917
H	1.805512	-0.44609	-3.16402
H	2.85158	-2.61548	-3.76813
N	0.87246	-1.15193	-1.46887
K	0.649638	1.554917	-0.38084
O	0.101866	-0.75828	1.132762
O	-0.32318	-0.79755	2.541932
C	4.056938	1.829438	-1.32483
C	4.146955	1.255691	0.110294
H	3.291224	1.286463	-1.89415
H	3.768375	2.884611	-1.27323
H	4.997675	1.755659	-1.88288
C	5.298984	1.977256	0.840049
C	4.509673	-0.24621	-0.01413
H	5.387956	1.580562	1.858385
H	6.269526	1.864663	0.341561
H	5.0662	3.043075	0.919476
H	5.41402	-0.43441	-0.60433
H	4.679479	-0.67392	0.984286
H	3.67399	-0.78159	-0.47765
O	2.965887	1.418824	0.780607
K	2.172739	-0.08206	2.625341
C	-2.4283	2.341766	-2.36229
C	-2.6342	2.863184	-0.92001
H	-1.39873	2.54205	-2.68471
H	-2.58169	1.257679	-2.37949
H	-3.10186	2.80094	-3.09533
C	-4.1188	2.627158	-0.54688
C	-2.37247	4.383326	-0.90821
H	-4.30026	2.998764	0.469366
H	-4.8248	3.128596	-1.21895
H	-4.33472	1.551874	-0.57043
H	-3.01996	4.94258	-1.5945
H	-2.52175	4.768379	0.105413
H	-1.32981	4.5744	-1.18557
O	-1.8021	2.217374	-0.04794
K	-2.36402	0.631314	1.800515
H	0.02674	-2.78137	1.286688

**5-3K**			
C	-3.49622	1.972301	0.657948
C	-2.48667	1.013872	0.646765
C	-2.75241	-0.28847	1.078372
C	-4.03628	-0.6011	1.52303
C	-5.04451	0.357903	1.537112
C	-4.77616	1.651112	1.101845
H	-3.27772	2.979444	0.315229
H	-1.49301	1.293696	0.295684
H	-4.24808	-1.61178	1.865788
H	-6.03702	0.096347	1.892352
H	-5.55762	2.404847	1.112064
C	-1.65866	-1.35621	1.035623
C	-1.70127	-2.13128	-0.3041
C	-2.56802	-1.82463	-1.35639
C	-2.45336	-2.49987	-2.56515
H	-3.31674	-1.05447	-1.22065
C	-0.67398	-3.74472	-1.59968
C	-1.4853	-3.48705	-2.69798
H	-3.1159	-2.25964	-3.39132
H	0.089946	-4.51852	-1.65821
H	-1.36249	-4.04812	-3.61754
N	-0.75968	-3.08715	-0.44148
K	0.745375	-0.14886	-1.19857
O	-0.40135	-0.87125	1.228045
C	4.324486	-0.70739	-1.20431
C	3.99421	-0.32602	0.255783
H	4.107671	-1.77073	-1.35862
H	3.701563	-0.1251	-1.89461
H	5.370883	-0.52567	-1.47355
C	4.2123	1.195188	0.41332
C	4.983246	-1.06379	1.181068
H	4.109557	1.476245	1.470161
H	5.202979	1.526602	0.082802
H	3.451713	1.735546	-0.16094
H	6.029768	-0.78818	1.008448
H	4.733994	-0.83896	2.223902
H	4.890462	-2.14728	1.034217
O	2.696957	-0.66557	0.56461
K	1.414081	-2.65614	1.429843
C	-0.88259	3.324052	-2.12565
C	0.218902	3.457048	-1.05116
H	-0.53918	2.644661	-2.91598
H	-1.78279	2.891345	-1.67835

H	-1.14981	4.27834	-2.59479
C	-0.27403	4.446068	0.027639
C	1.481289	4.044704	-1.71285
H	0.511432	4.581148	0.78113
H	-0.53418	5.433675	-0.371
H	-1.15484	4.029752	0.527244
H	1.313807	5.025412	-2.17434
H	2.273068	4.143097	-0.96288
H	1.840359	3.355871	-2.48581
O	0.494115	2.236918	-0.49108
K	1.069662	1.190922	1.725869
H	-1.96104	-2.12118	1.79423

^{2}2-3K

O	-0.74866	-1.89048	0.948664
O	-2.0385	1.047584	-0.66269
K	0.158842	2.181562	-1.29161
K	-1.40266	0.418399	1.884946
K	-2.01614	-1.54583	-1.19559
C	-0.56274	-2.99418	1.769095
C	-3.20972	1.746854	-0.91952
C	-2.93266	3.258063	-1.07356
H	-2.3154	3.448742	-1.96268
H	-3.84326	3.852203	-1.19221
H	-2.40566	3.629879	-0.18607
C	-3.87192	1.252106	-2.22058
H	-4.19908	0.209777	-2.11309
H	-4.75406	1.835012	-2.50188
H	-3.15153	1.303657	-3.0445
C	-4.21792	1.569095	0.233375
H	-4.40057	0.502452	0.408246
H	-3.82216	2.009384	1.157686
H	-5.18281	2.045872	0.036921
C	-0.84381	-4.29478	0.991923
H	-0.7032	-5.19494	1.598437
H	-1.88008	-4.29824	0.63048
H	-0.17397	-4.36558	0.127556
C	0.882152	-3.03836	2.295597
H	1.10386	-2.11596	2.844701
H	1.063371	-3.88505	2.965327
H	1.58659	-3.10535	1.460921
C	-1.52455	-2.93893	2.972781
H	-1.30109	-2.06211	3.59571
H	-2.55815	-2.85771	2.617718
H	-1.45434	-3.8187	3.620001
C	1.657732	2.778598	2.296437
C	2.985091	3.181493	2.383988
C	3.941468	2.429795	1.70779
C	3.537787	1.328314	0.97156
C	2.174844	0.984548	0.923801
N	1.245659	1.718779	1.597419
H	4.992662	2.69217	1.767116
H	0.884736	3.33855	2.818513
H	3.256847	4.047882	2.975179
H	4.263978	0.700352	0.470343
C	1.660106	-0.16005	0.204163
H	0.801318	-0.69156	0.638793
C	2.124663	-0.69324	-1.03501
C	3.044779	-0.05937	-1.90789
C	1.547081	-1.9194	-1.46101
C	3.372392	-0.63159	-3.12737
H	3.501449	0.884199	-1.62636
C	1.889977	-2.48341	-2.67584
H	0.837591	-2.38939	-0.78267
C	2.804051	-1.84435	-3.5182
H	4.082651	-0.13397	-3.77994
H	1.459199	-3.43548	-2.97138
H	3.075242	-2.2901	-4.46905

$[KOtBu]_4$

O	1.550123	-1.19305	1.229717
O	-1.19805	-1.44849	-1.34246
O	1.091188	1.549	-1.32067
O	-1.44471	1.092096	1.434024
K	-1.05532	-1.49819	1.276929
K	1.395621	-1.05592	-1.38683
K	1.157696	1.399922	1.298779
K	-1.49917	1.154341	-1.1879
C	2.475527	-1.90253	1.958677
C	-1.91236	-2.31019	-2.14133
C	1.742919	2.471356	-2.10533
C	-2.30576	1.741077	2.287562
C	1.934336	-2.21984	3.369195
H	1.045676	-2.8592	3.294134
H	2.661038	-2.73536	4.006851
H	1.639312	-1.2883	3.864586
C	3.782507	-1.09605	2.115083
H	4.570874	-1.64481	2.64213

H	4.158801	-0.81615	1.124964
H	3.587651	-0.17353	2.676571
C	2.814165	-3.23754	1.261522
H	1.892049	-3.80447	1.092604
H	3.275699	-3.04348	0.285073
H	3.505133	-3.86416	1.836285
C	-1.10582	-3.59777	-2.41463
H	-0.81563	-4.05487	-1.46221
H	-1.65903	-4.34041	-3.00022
H	-0.18919	-3.35497	-2.96692
C	-2.24206	-1.6517	-3.49806
H	-2.76053	-2.32298	-4.19164
H	-2.88328	-0.77485	-3.34261
H	-1.31486	-1.3125	-3.97289
C	-3.24124	-2.70915	-1.46451
H	-3.80759	-1.80546	-1.21384
H	-3.87187	-3.35015	-2.09046
H	-3.03815	-3.25175	-0.53248
C	-2.36241	1.026557	3.654774
H	-1.35	0.945007	4.065352
H	-2.99246	1.542093	4.388042
H	-2.76022	0.011467	3.530221
C	-1.84836	3.196509	2.523539
H	-1.75063	3.708445	1.559896
H	-2.53732	3.771528	3.15217
H	-0.86614	3.205522	3.012798
C	-3.73412	1.774543	1.702848
H	-3.74176	2.347015	0.766599
H	-4.0596	0.752907	1.477982
H	-4.46806	2.230897	2.376262
C	1.77255	3.852226	-1.41587
H	2.231309	4.634604	-2.03057
H	0.749658	4.159356	-1.17184
H	2.340179	3.790431	-0.47876
C	3.199785	2.033772	-2.36861
H	3.211671	1.090695	-2.92968
H	3.7764	2.767832	-2.94227
H	3.707943	1.865405	-1.41276
C	1.034017	2.628552	-3.46757
H	0.955209	1.649276	-3.95236
H	0.017872	3.014976	-3.31822
H	1.551668	3.311724	-4.15006

2-4K

O	-1.88177	-1.10302	-1.40327
O	1.530783	-1.82828	-0.08004
O	-1.09233	-0.51524	2.181653
K	0.558684	-0.33307	-2.00831
K	-0.88862	-2.56057	0.537202
K	-2.41652	0.780863	0.319234
K	1.432147	0.083813	1.689329
C	-2.78646	-1.51539	-2.35814
C	2.620503	-2.65698	-0.23469
C	-1.55931	-0.54282	3.478187
C	-2.75458	-0.57977	-3.58485
H	-1.77032	-0.62541	-4.06857
H	-3.50463	-0.83512	-4.34141
H	-2.92292	0.452716	-3.26238
C	-4.22218	-1.51087	-1.79001
H	-4.96733	-1.89369	-2.49568
H	-4.25966	-2.12622	-0.88393
H	-4.51929	-0.48928	-1.521
C	-2.46427	-2.94794	-2.83244
H	-1.4341	-2.99086	-3.2032
H	-2.54854	-3.64988	-1.99338
H	-3.13019	-3.29958	-3.62804
C	2.278947	-4.09408	0.214328
H	1.459854	-4.48844	-0.4004
H	3.12295	-4.78734	0.129788
H	1.954843	-4.08139	1.262027
C	3.817723	-2.16101	0.604934
H	4.707582	-2.79128	0.498005
H	4.084927	-1.14284	0.301846
H	3.544729	-2.15583	1.668524
C	3.067761	-2.71007	-1.71194
H	3.370862	-1.70934	-2.04005
H	3.911999	-3.38691	-1.88301
H	2.231156	-3.05068	-2.33456
C	-1.10921	0.713973	4.252634
H	-1.51348	0.761266	5.269728
H	-0.01498	0.738898	4.332181
H	-1.42457	1.611957	3.711107
C	-3.10289	-0.5937	3.506607
H	-3.45312	-1.447	2.914783
H	-3.51374	-0.6862	4.517647
H	-3.52102	0.321388	3.067693
C	-1.03018	-1.78672	4.223289
H	-1.39164	-2.69982	3.734457

H	0.06518	-1.80093	4.189767	H	-0.21663	0.071105	4.97897
H	-1.33961	-1.82592	5.273391	H	-1.32445	0.723916	3.795786
N	2.393048	1.738493	-1.26689	H	-0.80031	-0.98903	3.688655
C	2.356866	2.413867	-0.07223	C	0.981297	1.92947	3.204218
C	3.569843	1.266456	-1.70005	H	1.821814	2.233366	2.5765
C	3.576008	2.542697	0.679838	H	0.100799	2.504093	2.894801
C	4.771412	1.355505	-1.02872	H	1.212897	2.192885	4.241328
H	3.542558	0.761048	-2.66738	C	0.512669	0.653048	-4.3218
C	4.751461	2.018268	0.211397	H	1.287552	1.262424	-3.84473
H	3.551414	3.092007	1.616999	H	0.812313	0.485911	-5.36244
H	5.679163	0.937222	-1.44545	H	-0.41808	1.232782	-4.32159
H	5.665324	2.132469	0.788265	C	-0.72894	-1.51159	-4.30321
C	1.159739	2.921765	0.474584	H	-0.37792	-1.81515	-5.29525
H	1.268242	3.393103	1.44979	H	-0.96019	-2.41683	-3.73052
C	-0.11302	3.119719	-0.14066	H	-1.66133	-0.95009	-4.45512
C	-1.17217	3.688732	0.626531	C	1.633586	-1.44036	-3.52347
C	-0.43529	2.823885	-1.49466	H	1.496512	-2.40403	-3.01904
C	-2.42631	3.934226	0.093649	H	2.014436	-1.641	-4.5311
H	-0.96973	3.964232	1.658617	H	2.397361	-0.88694	-2.97121
C	-1.70132	3.065385	-2.01624	C	-2.92808	-3.85776	1.370465
H	0.360575	2.471339	-2.13775	H	-2.7557	-3.28813	2.290595
C	-2.72292	3.6066	-1.23579	H	-3.58242	-4.70381	1.6075
H	-3.18604	4.39843	0.718272	H	-1.96395	-4.26508	1.042541
H	-1.88968	2.837486	-3.06233	C	-4.90177	-2.45473	0.750801
H	-3.70058	3.813288	-1.65795	H	-5.31856	-1.75768	0.015188
				H	-5.6134	-3.27644	0.886548
**TS1-4K**				H	-4.81957	-1.92501	1.708359
O	0.410226	0.15414	1.679347	C	-3.68317	-3.74323	-1.01108
O	-0.12855	-0.38989	-2.24909	H	-4.15323	-3.12378	-1.78466
O	-2.44468	1.925893	-0.43357	H	-2.7026	-4.05676	-1.38538
O	-2.67546	-1.84879	0.073178	H	-4.30233	-4.6371	-0.87752
K	-0.04426	-2.07103	-0.18307	C	-4.53943	2.972655	0.114292
K	0.162287	1.757724	-0.72753	H	-5.14953	3.877557	0.020355
K	-2.44933	0.255634	1.514609	H	-5.07644	2.144871	-0.36213
K	-2.74005	-0.19382	-1.97251	H	-4.44683	2.745152	1.18396
C	0.71285	0.429892	3.039546	C	-2.38753	4.240144	0.218492
C	0.305005	-0.65665	-3.53133	H	-1.41518	4.420776	-0.2564
C	-3.13948	3.112838	-0.51973	H	-2.93285	5.190212	0.225712
C	-3.50763	-2.92597	0.285512	H	-2.2043	3.940192	1.25636
C	1.923131	-0.38848	3.504248	C	-3.31716	3.533445	-1.99324
H	2.827768	-0.10221	2.970026	H	-2.33719	3.595628	-2.47887
H	2.10371	-0.24896	4.574063	H	-3.91029	2.781894	-2.52884
H	1.753334	-1.45112	3.310828	H	-3.82086	4.499326	-2.10915
C	-0.48177	0.033191	3.919151	N	2.612278	-2.478	0.651011

C	3.292448	-1.30306	0.649398
C	3.191199	-3.56562	1.163012
C	4.602303	-1.25823	1.16823
C	4.461608	-3.58942	1.714495
H	2.597059	-4.47833	1.130273
C	5.177803	-2.3947	1.702324
H	5.162348	-0.33286	1.125938
H	4.874387	-4.50442	2.12271
H	6.188655	-2.35479	2.098299
C	2.565192	-0.1455	0.120472
H	2.029791	-0.43939	-0.787
H	1.406244	0.047323	1.063045
C	3.24486	1.15209	-0.09084
C	3.875065	1.876571	0.938514
C	3.186287	1.783606	-1.34838
C	4.392583	3.148422	0.728013
H	3.950352	1.438093	1.927723
C	3.688344	3.064114	-1.5584
H	2.75083	1.237791	-2.18087
C	4.29457	3.761302	-0.51826
H	4.87173	3.670814	1.551181
H	3.625511	3.507506	-2.54863
H	4.697274	4.755887	-0.67861

TS2-4K

K	-2.54853	-1.32905	-1.94774
K	-0.25095	1.099615	-0.46737
C	-2.59419	-4.21043	0.331254
C	-3.29256	-3.00839	1.007935
H	-2.68759	-4.13957	-0.7605
H	-1.52622	-4.19448	0.573919
H	-3.0061	-5.17961	0.634566
C	-3.23407	-3.22672	2.537081
C	-4.78122	-3.01847	0.592775
H	-3.66278	-2.35701	3.046545
H	-3.77243	-4.12126	2.868506
H	-2.19249	-3.33502	2.865646
H	-5.29489	-3.95509	0.836061
H	-5.31254	-2.2014	1.094389
H	-4.87125	-2.85837	-0.48891
O	-2.68999	-1.82601	0.650841
K	-0.46685	-1.11968	2.079302
C	6.011189	-1.42307	-0.41624
C	5.142914	-0.37842	-0.105
C	4.035807	-0.58996	0.711902
C	3.828052	-1.86654	1.23592
C	4.689602	-2.90986	0.929789
C	5.784896	-2.69431	0.094039
H	6.862725	-1.23914	-1.06433
H	5.328803	0.605822	-0.52248
H	2.955095	-2.0301	1.853989
H	4.506981	-3.89883	1.340451
H	6.457185	-3.51102	-0.15016
C	3.064304	0.527729	1.090266
C	3.164011	1.716457	0.136569
C	3.792648	2.89483	0.537825
C	3.861607	3.968969	-0.33875
H	4.216003	2.963416	1.534296
C	2.690149	2.630792	-1.91984
C	3.299903	3.838287	-1.60224
H	4.344149	4.894063	-0.03928
H	2.235198	2.485344	-2.898
H	3.327608	4.646643	-2.32419
N	2.615754	1.595503	-1.08053
O	1.756414	0.111375	1.277721
O	1.317801	-1.1581	0.140823
S	1.046378	-2.10974	-1.4834
O	0.018233	-1.38652	-2.34558
C	0.932391	-3.84841	-2.04847
H	1.737214	-4.42248	-1.58386
H	-0.03244	-4.23262	-1.71212
H	1.00105	-3.89735	-3.13829
C	2.643644	-1.72968	-2.18768
H	2.783343	-0.66121	-2.02104
H	3.412547	-2.28056	-1.64306
H	2.646173	-1.97574	-3.25126
H	3.41533	0.90596	2.068316
K	-3.56576	0.676588	0.954428
C	-1.97351	2.690998	-3.17145
C	-3.1516	2.209613	-2.29615
H	-1.17497	3.09446	-2.53618
H	-1.56616	1.843884	-3.73395
H	-2.257	3.475701	-3.882
C	-4.29846	1.772176	-3.2322
C	-3.64312	3.406927	-1.45473
H	-5.12639	1.381938	-2.62985
H	-4.68099	2.583957	-3.86116
H	-3.9515	0.969883	-3.89557

H	-3.91429	4.278755	-2.06083
H	-4.52881	3.117263	-0.87494
H	-2.85951	3.70501	-0.74987
O	-2.7654	1.167783	-1.48553
C	-0.12876	3.391226	1.990821
C	-0.89599	2.329218	2.812662
H	0.754922	2.931393	1.53401
H	-0.77942	3.778499	1.19736
H	0.211946	4.240035	2.59523
C	-2.06745	3.030516	3.531498
C	0.074931	1.7728	3.878679
H	-2.63097	2.289167	4.109599
H	-1.7465	3.826679	4.213477
H	-2.74154	3.472103	2.787458
H	0.492022	2.548971	4.53114
H	-0.45458	1.050812	4.513889
H	0.898843	1.26105	3.369831
O	-1.36073	1.322441	2.001731

^{3}3-4K

O	2.481555	1.781378	-0.44859
O	0.396663	-0.11766	2.049255
O	2.249747	-1.97243	-0.61589
K	0.010344	1.939553	0.470664
K	2.873854	-0.2167	1.208158
K	1.854979	0.008943	-2.29875
K	-0.2663	-1.83648	0.169633
C	3.297209	2.872525	-0.63814
C	0.00114	-0.23316	3.364287
C	2.900624	-3.14996	-0.89835
C	3.176489	3.85876	0.544075
H	3.405582	3.338745	1.480937
H	3.844524	4.722972	0.457975
H	2.149414	4.239178	0.612682
C	2.914389	3.624122	-1.93093
H	3.511722	4.527114	-2.09992
H	3.048494	2.968023	-2.79974
H	1.858054	3.911372	-1.89027
C	4.774631	2.439726	-0.75147
H	5.094615	1.952513	0.177753
H	4.883615	1.713092	-1.56428
H	5.457741	3.274682	-0.94364
C	1.115975	0.259333	4.312026
H	1.385314	1.289021	4.050974
H	0.827864	0.237194	5.36896
H	2.009014	-0.36918	4.199695
C	-0.30937	-1.70461	3.717608
H	-0.56847	-1.85074	4.772159
H	-1.15658	-2.06032	3.118629
H	0.562773	-2.32777	3.488494
C	-1.26801	0.599201	3.639279
H	-2.05874	0.29605	2.947175
H	-1.64066	0.489321	4.664022
H	-1.06063	1.664404	3.475601
C	2.308616	-4.31517	-0.07485
H	2.836992	-5.26368	-0.2227
H	2.344833	-4.06256	0.990871
H	1.25922	-4.47516	-0.35447
C	2.767609	-3.50481	-2.39488
H	3.255933	-2.7344	-3.00527
H	3.220321	-4.46838	-2.65448
H	1.707499	-3.53356	-2.66826
C	4.403225	-3.04042	-0.5603
H	4.833874	-2.18825	-1.0977
H	4.531738	-2.86667	0.515763
H	4.974437	-3.93815	-0.82204
N	-3.25023	-1.24133	0.205634
C	-4.25839	-0.96221	-0.65502
C	-3.00532	-2.51343	0.49515
C	-5.01812	-2.00489	-1.24155
C	-3.70686	-3.59438	-0.03151
H	-2.18812	-2.68901	1.191638
C	-4.74018	-3.3193	-0.92636
H	-5.79095	-1.75445	-1.95852
H	-3.45515	-4.60952	0.254408
H	-5.31857	-4.12481	-1.36801
C	-4.55942	0.385406	-1.02356
H	-5.36236	0.483505	-1.74634
C	-3.9207	1.611295	-0.65264
C	-4.31248	2.785533	-1.33909
C	-2.90266	1.724739	0.319155
C	-3.71297	4.006739	-1.08851
H	-5.09437	2.714599	-2.08932
C	-2.32163	2.961084	0.570839
H	-2.6005	0.838476	0.858483
C	-2.70514	4.104218	-0.12966
H	-4.03308	4.888671	-1.63417
H	-1.54723	3.035897	1.324967

H	-2.23434	5.060586	0.07552
O	-0.69873	0.350685	-1.55068
O	-0.75911	-0.66603	-2.40225

^{2}2-4K

O	-1.84556	-1.49133	-0.82448
O	1.919656	-1.48482	-0.50693
O	-0.23624	-0.41335	2.325899
K	0.152164	-0.50824	-2.15453
K	-0.0971	-2.61722	0.805883
K	-2.4198	0.206959	1.025097
K	2.111381	0.24532	1.384466
C	-2.84078	-2.19314	-1.48516
C	2.995126	-2.22417	-0.96551
C	-0.31707	-0.41377	3.708618
C	-3.37768	-1.38354	-2.68216
H	-2.58085	-1.2185	-3.41937
H	-4.20357	-1.8806	-3.20077
H	-3.7247	-0.4038	-2.33805
C	-4.02272	-2.48696	-0.53834
H	-4.80488	-3.09589	-1.00189
H	-3.66325	-3.01725	0.350945
H	-4.49396	-1.54986	-0.21296
C	-2.30372	-3.53929	-2.01022
H	-1.43957	-3.37137	-2.66358
H	-1.97506	-4.16973	-1.17495
H	-3.04836	-4.10678	-2.57702
C	2.949084	-3.66239	-0.41129
H	2.03661	-4.16587	-0.75423
H	3.799911	-4.27439	-0.72643
H	2.945565	-3.6342	0.685175
C	4.329731	-1.58476	-0.52796
H	5.20797	-2.11737	-0.90589
H	4.378654	-0.5505	-0.88684
H	4.400651	-1.57815	0.567698
C	2.989644	-2.30839	-2.50656
H	3.057735	-1.30108	-2.93714
H	3.820666	-2.89461	-2.91041
H	2.058519	-2.77673	-2.84886
C	0.075171	0.963675	4.281626
H	-0.03298	1.024364	5.369096
H	1.122468	1.195606	4.04626
H	-0.54855	1.741881	3.828558
C	-1.75184	-0.73271	4.181362
H	-2.08834	-1.67576	3.73539
H	-1.83618	-0.82509	5.268212
H	-2.444	0.06402	3.877022
C	0.621572	-1.47834	4.31338
H	0.329125	-2.47904	3.97425
H	1.655309	-1.30516	3.988789
H	0.616549	-1.48512	5.407609
N	1.581047	2.11648	-1.45491
C	1.453058	3.010209	-0.43713
C	2.806999	1.825233	-1.88476
C	2.596907	3.579009	0.173724
C	3.976655	2.356817	-1.34711
H	2.875429	1.110392	-2.70084
C	3.860164	3.252008	-0.28612
H	2.469713	4.28845	0.984646
H	4.941396	2.071361	-1.74918
H	4.74151	3.696095	0.165043
C	0.163648	3.380405	0.066784
H	0.189144	3.966303	0.981709
C	-1.14331	3.129483	-0.45679
C	-2.25674	3.473305	0.351204
C	-1.40869	2.597094	-1.74093
C	-3.55589	3.260599	-0.08416
H	-2.08163	3.924554	1.324071
C	-2.71037	2.382454	-2.16635
H	-0.57396	2.395179	-2.39876
C	-3.79132	2.700607	-1.3425
H	-4.3904	3.550763	0.546727
H	-2.89225	1.977513	-3.15631
H	-4.80677	2.540524	-1.68869

^{3}6-4K

O	2.136372	-0.46248	-1.64138
O	0.164425	2.477449	-0.4635
O	1.78806	0.227248	1.99209
K	-0.3349	0.379396	-1.96689
K	2.590422	1.610244	-0.09956
K	1.720802	-1.91933	0.481189
K	-0.61702	1.281773	1.71644
C	2.921572	-0.88426	-2.69299
C	-0.19422	3.765294	-0.79616
C	2.38689	0.184467	3.23296
C	2.124658	-1.80857	-3.63722
H	1.289948	-1.2572	-4.08887

H	2.730381	-2.21563	-4.45427
H	1.70551	-2.64281	-3.06575
C	4.148738	-1.66801	-2.1793
H	4.834963	-1.96883	-2.97828
H	4.704447	-1.05133	-1.46379
H	3.825397	-2.58081	-1.66304
C	3.431677	0.320691	-3.51089
H	2.581372	0.919819	-3.85522
H	4.061493	0.962685	-2.88242
H	4.022771	0.029449	-4.38603
C	1.025605	4.70839	-0.71527
H	1.796776	4.37714	-1.42235
H	0.784326	5.750918	-0.94978
H	1.443699	4.677437	0.298197
C	-1.28234	4.301862	0.158931
H	-1.60199	5.32143	-0.08367
H	-2.16271	3.651303	0.118731
H	-0.89664	4.314144	1.186929
C	-0.75252	3.827513	-2.23469
H	-1.65459	3.209175	-2.30567
H	-1.01819	4.842051	-2.55134
H	-0.00141	3.441134	-2.9346
C	1.412014	-0.36387	4.296294
H	1.870104	-0.47288	5.28545
H	0.55127	0.30811	4.40443
H	1.03318	-1.34112	3.979128
C	3.633065	-0.72805	3.211787
H	4.318979	-0.39506	2.4246
H	4.178994	-0.736	4.161217
H	3.341693	-1.76397	2.995273
C	2.839041	1.593869	3.670546
H	3.584717	1.984789	2.967416
H	1.982442	2.277524	3.663002
H	3.282043	1.614177	4.672195
N	-2.84995	0.13091	-0.6275
C	-3.0448	-0.23281	0.680774
C	-3.55711	1.158179	-1.11834
C	-3.95869	0.537345	1.479764
C	-4.44965	1.933569	-0.40908
H	-3.38178	1.385417	-2.17145
C	-4.63873	1.598584	0.944042
H	-4.12259	0.239812	2.511616
H	-4.97544	2.755262	-0.87923
H	-5.33444	2.164051	1.558092

C	-2.33726	-1.29425	1.282519
H	-2.54508	-1.43936	2.340683
C	-1.56369	-2.33301	0.681691
C	-0.95036	-3.31066	1.51886
C	-1.36831	-2.51933	-0.71484
C	-0.21678	-4.36871	1.009624
H	-1.09509	-3.23209	2.593472
C	-0.62393	-3.582	-1.21495
H	-1.8893	-1.85484	-1.39087
C	-0.02193	-4.51401	-0.3702
H	0.202632	-5.10166	1.694867
H	-0.52346	-3.69114	-2.29176
H	0.538977	-5.35189	-0.77034
O	-4.50796	-2.32664	-1.38166
O	-4.72066	-2.62415	-0.23179

4-4K

O	0.816096	2.406951	0.537542
O	0.805953	-1.1537	1.83425
O	2.91826	-0.25656	-1.14957
K	-1.0717	0.552609	1.025282
K	2.610456	0.680089	1.276129
K	1.155131	1.535481	-1.9245
K	0.956313	-1.97464	-0.65781
C	0.649096	3.724954	0.886527
C	0.89899	-1.86327	3.005976
C	4.100242	-0.57433	-1.77438
C	-0.48169	3.882515	1.92777
H	-0.25837	3.25784	2.800498
H	-0.61183	4.914819	2.27197
H	-1.43421	3.552477	1.496177
C	0.277603	4.577011	-0.34843
H	0.078018	5.627592	-0.10917
H	1.098489	4.55336	-1.07697
H	-0.6174	4.155054	-0.81873
C	1.946535	4.300359	1.494067
H	2.197938	3.753427	2.411205
H	2.768292	4.171832	0.780115
H	1.874859	5.364149	1.747485
C	2.191811	-1.50453	3.77683
H	2.187586	-0.43656	4.030134
H	2.308496	-2.0644	4.711249
H	3.065361	-1.71643	3.147881
C	0.928141	-3.38337	2.730567

H	0.981605	-3.98929	3.642263

H	1.802086	-3.62464	2.113211
C	-0.29755	-1.5565	3.933057
H	-1.23367	-1.80929	3.424002
H	-0.26585	-2.10539	4.881091
H	-0.31434	-0.48428	4.16059
C	3.876979	-1.65993	-2.8498
H	4.781753	-1.90002	-3.41935
H	3.527472	-2.58697	-2.3772
H	3.10495	-1.3236	-3.5503
C	4.705914	0.667133	-2.4647
H	4.824824	1.469587	-1.72818
H	5.680673	0.475497	-2.92699
H	4.029383	1.024097	-3.25159
C	5.132267	-1.10985	-0.75835
H	5.373079	-0.33073	-0.02417
H	4.703558	-1.96192	-0.21931
H	6.071729	-1.43104	-1.22205
N	-2.64389	-1.90705	0.182607
C	-2.71731	-1.92759	-1.14939
C	-2.37898	-3.04973	0.823777
C	-2.51964	-3.10131	-1.88186
C	-2.15859	-4.25835	0.178515
H	-2.32741	-2.98905	1.906804
C	-2.2312	-4.27984	-1.21251
H	-2.57547	-3.06621	-2.96438
H	-1.94247	-5.15344	0.751212
H	-2.07573	-5.20373	-1.76155
C	-2.91912	-0.63322	-1.93297
H	-3.62342	-0.85138	-2.74991
C	-3.48397	0.535907	-1.14033
C	-2.96475	1.812854	-1.3519
C	-4.54323	0.382295	-0.24563
C	-3.46654	2.909193	-0.65807
H	-2.14809	1.920247	-2.05406
C	-5.04974	1.477517	0.447965
H	-4.96363	-0.60232	-0.07253
C	-4.50685	2.743931	0.251837
H	-3.04153	3.893976	-0.82488
H	-5.8719	1.340079	1.143669
H	-4.89842	3.597355	0.796365
O	-1.71633	-0.32488	-2.58533
O	-0.70682	-0.19269	-1.55126

H	0.029018	-3.67277	2.177721

3-1K-DMSO

C	0.392263	2.747584	-1.50201
C	-0.65625	2.001197	-0.98371
C	-0.7268	1.700227	0.399634
C	0.314621	2.199992	1.21656
C	1.348285	2.958372	0.6903
C	1.404275	3.23282	-0.67411
H	0.407717	2.974382	-2.56449
H	-1.45136	1.65745	-1.63402
H	0.293297	1.974921	2.276715
H	2.124961	3.33245	1.35123
H	2.213505	3.828195	-1.08492
C	-1.76893	0.93347	1.017927
C	-2.78461	0.141719	0.404393
C	-3.90594	-0.26957	1.170399
C	-4.89329	-1.03452	0.59546
H	-3.97365	0.038872	2.207939
C	-3.64212	-0.99502	-1.42721
C	-4.77753	-1.40483	-0.74923
H	-5.75579	-1.34207	1.179023
H	-3.50128	-1.27865	-2.46967
H	-5.53587	-1.99792	-1.24653
N	-2.66164	-0.2646	-0.89033
K	0.074658	-1.02424	-0.87772
O	-0.25171	-0.97836	1.819419
O	0.983306	-0.62456	1.787677
H	-1.84845	1.032032	2.093461
S	3.811565	-1.29479	-0.51962
O	2.556066	-1.60193	-1.32478
C	3.602367	0.373375	0.139225
H	4.453974	0.615618	0.779417
H	3.5742	1.058404	-0.70898
H	2.664848	0.439349	0.696353
C	3.654589	-2.18189	1.046375
H	3.675211	-3.24781	0.814493
H	4.507689	-1.93109	1.681464
H	2.711897	-1.90344	1.528789

3-1K-2DMSO

C	-2.39655	-3.12542	1.572453
C	-2.56528	-1.77504	1.302256
C	-2.90946	-1.32407	0.004471

C	-3.08831	-2.31393	-0.98706
C	-2.92466	-3.6625	-0.706
C	-2.56731	-4.08239	0.571542
H	-2.14562	-3.43785	2.58263
H	-2.44252	-1.04507	2.092745
H	-3.34683	-1.99923	-1.99186
H	-3.06766	-4.39274	-1.49713
H	-2.43602	-5.13731	0.789854
C	-3.07477	0.059907	-0.3545
C	-2.5786	1.198082	0.323799
C	-3.04839	2.500498	-0.02416
C	-2.56692	3.609052	0.626366
H	-3.80468	2.593346	-0.79654
C	-1.15668	2.165138	1.889185
C	-1.59283	3.45525	1.626862
H	-2.9415	4.596394	0.371013
H	-0.38362	1.997933	2.639131
H	-1.20166	4.300224	2.182027
N	-1.60254	1.070868	1.273788
K	0.19548	-0.92861	0.164461
O	-1.31895	0.001892	-2.2444
O	-0.83805	-1.15784	-2.40904
H	-3.70262	0.257933	-1.21522
S	3.834162	-1.41279	-0.09293
O	2.568783	-2.03444	0.46589
C	4.412111	-0.23072	1.14225
H	5.394538	0.150672	0.853793
H	3.673717	0.571548	1.141584
H	4.455016	-0.72275	2.116717
C	5.126034	-2.65336	0.157228
H	4.895193	-3.49952	-0.49076
H	6.098651	-2.235	-0.11137
H	5.114434	-2.97676	1.200241
S	1.924054	2.390336	-0.97335
O	1.864889	1.155637	-0.0811
C	0.283817	2.626627	-1.67929
H	0.298729	3.535284	-2.28602
H	0.045408	1.761137	-2.29945
H	-0.45777	2.705832	-0.883
C	1.888798	3.807137	0.147016
H	2.800074	3.777962	0.74551
H	1.85967	4.729238	-0.43774
H	1.00784	3.727903	0.787888

3-1K-3DMSO

C	-0.46531	3.575276	-1.87332
C	-1.52587	2.769696	-1.4799
C	-1.94241	2.715407	-0.13046
C	-1.24447	3.528206	0.788001
C	-0.18958	4.335219	0.387355
C	0.21762	4.359345	-0.94422
H	-0.17569	3.60162	-2.92021
H	-2.05391	2.172094	-2.21295
H	-1.53544	3.491053	1.832072
H	0.326278	4.945498	1.123525
H	1.045905	4.986775	-1.2573
C	-3.01382	1.87924	0.349162
C	-3.45585	0.649165	-0.2008
C	-4.61825	0.015124	0.321918
C	-5.02655	-1.19879	-0.17414
H	-5.17332	0.509054	1.112319
C	-3.14645	-1.15028	-1.63595
C	-4.284	-1.81033	-1.19452
H	-5.91907	-1.67694	0.219413
H	-2.51462	-1.60718	-2.3957
H	-4.58022	-2.75831	-1.62944
N	-2.72556	0.025313	-1.17011
K	0.123969	0.259922	-0.65539
O	-1.72319	0.72394	2.128619
O	-0.47254	0.931509	1.9931
H	-3.54895	2.231278	1.222586
S	3.736439	-0.43312	-1.26839
O	2.68662	0.648019	-1.46277
C	2.988957	-2.00185	-1.74124
H	3.737955	-2.79269	-1.65404
H	2.150046	-2.18973	-1.06746
H	2.620249	-1.93189	-2.76758
C	4.851987	-0.27298	-2.68333
H	5.375003	0.678797	-2.58382
H	5.571505	-1.09496	-2.6826
H	4.26323	-0.27285	-3.60303
S	-0.31434	-3.29243	0.398547
O	-0.03655	-2.44497	-0.83762
C	-1.18793	-2.24912	1.578451
H	-1.51578	-2.86175	2.421404
H	-0.48373	-1.49075	1.918121
H	-2.04196	-1.76941	1.095245
C	-1.71436	-4.34572	-0.04986

H	-1.38378	-5.00608	-0.85239
H	-2.01784	-4.93724	0.816945
H	-2.53495	-3.71345	-0.39515
S	2.300196	-0.09333	2.385593
O	1.83765	-0.94233	1.206491
C	4.075517	-0.42761	2.524512
H	4.509361	0.197553	3.308033
H	4.182859	-1.47991	2.790864
H	4.555205	-0.24141	1.562575
C	2.445741	1.60807	1.78493
H	1.440305	2.027865	1.741738
H	3.05864	2.183185	2.482416
H	2.883713	1.58832	0.783419

6-1K-DMSO

C	3.578399	-1.25991	-1.23534
C	2.586814	-0.42134	-0.73622
C	2.353068	-0.29469	0.66014
C	3.217581	-1.04781	1.497842
C	4.199258	-1.87896	0.989245
C	4.387682	-2.01072	-0.38857
H	3.72779	-1.3119	-2.31144
H	1.985939	0.171618	-1.41545
H	3.093691	-0.96327	2.574749
H	4.831727	-2.43524	1.676312
H	5.158379	-2.66302	-0.78535
C	1.328876	0.521794	1.255441
C	0.260069	1.193813	0.642278
C	-0.44233	2.230777	1.350749
C	-1.45015	2.930894	0.747186
H	-0.12275	2.475361	2.358892
C	-1.15439	1.578657	-1.18209
C	-1.83372	2.614483	-0.57286
H	-1.94916	3.733258	1.284887
H	-1.42659	1.278577	-2.19464
H	-2.61435	3.156776	-1.09264
N	-0.1675	0.865275	-0.62553
K	0.055854	-1.87157	-0.31548
H	1.47334	0.74121	2.310001
O	2.132021	2.783293	-1.37671
O	2.537546	2.943541	-0.25079
S	-3.50378	-0.90077	-0.37226
O	-2.5181	-2.05733	-0.271
C	-5.12749	-1.63629	-0.07683
H	-5.88007	-0.84756	-0.01132
H	-5.35005	-2.29063	-0.92028
H	-5.09423	-2.22084	0.844955
C	-3.37703	0.006684	1.178059
H	-2.39486	0.479396	1.209073
H	-4.1428	0.784201	1.206242
H	-3.49579	-0.69313	2.008635

6-1K-2DMSO

C	1.341299	-2.80114	0.330845
C	0.1335	-2.1261	0.190749
C	-0.31664	-1.65354	-1.0685
C	0.522746	-1.96219	-2.17248
C	1.714377	-2.64998	-2.02658
C	2.156992	-3.06155	-0.76645
H	1.644211	-3.13977	1.318241
H	-0.52023	-2.00158	1.045839
H	0.204168	-1.65142	-3.16464
H	2.315931	-2.86268	-2.90677
H	3.095756	-3.59229	-0.65005
C	-1.53837	-0.93823	-1.29858
C	-2.42162	-0.33467	-0.39113
C	-3.67065	0.192627	-0.8749
C	-4.55956	0.790705	-0.02785
H	-3.89906	0.086703	-1.93103
C	-3.03768	0.391226	1.745466
C	-4.25462	0.897086	1.34547
H	-5.50213	1.172853	-0.41113
H	-2.75711	0.453233	2.79945
H	-4.93938	1.343176	2.056328
N	-2.12316	-0.178	0.944113
K	0.484599	0.482028	1.671107
H	-1.8565	-0.90399	-2.33792
O	-2.85531	-3.10251	1.211892
O	-3.01654	-3.2868	0.029191
S	-0.0347	3.309503	-0.88275
O	0.786815	2.647038	0.219847
C	-0.18423	2.099939	-2.21162
H	-0.8342	2.509217	-2.98877
H	0.815639	1.944288	-2.61966
H	-0.5905	1.153475	-1.82881
C	-1.75371	3.273346	-0.33391
H	-1.83159	3.930275	0.533293

H	-2.39381	3.647902	-1.13548
H	-2.04172	2.255249	-0.06869
S	3.968629	-0.19445	0.580205
O	3.014795	-0.10282	1.75915
C	3.079576	0.424747	-0.86496
H	3.784748	0.543793	-1.69115
H	2.331132	-0.32125	-1.13783
H	2.599785	1.374538	-0.61004
C	5.080702	1.222049	0.761689
H	5.664999	1.06414	1.668843
H	5.746729	1.283537	-0.10181
H	4.484111	2.131892	0.858557

6-1K-3DMSO

C	4.098193	-0.54105	-0.79991
C	3.014687	0.266779	-0.46635
C	2.611074	0.438753	0.88684
C	3.339552	-0.32112	1.844216
C	4.405864	-1.12921	1.495178
C	4.807645	-1.25239	0.162205
H	4.398482	-0.60312	-1.84395
H	2.503563	0.839423	-1.22997
H	3.065958	-0.22519	2.892045
H	4.940202	-1.66745	2.274161
H	5.655512	-1.87233	-0.11081
C	1.600285	1.346903	1.349667
C	0.870491	2.299715	0.616617
C	0.239156	3.399546	1.292958
C	-0.40444	4.376121	0.583098
H	0.326435	3.46364	2.373163
C	0.092755	3.1714	-1.40484
C	-0.47974	4.286887	-0.82234
H	-0.84606	5.222742	1.102972
H	0.031796	3.035948	-2.48497
H	-0.95163	5.055044	-1.42399
N	0.72585	2.196361	-0.74635
K	-0.50123	-0.30145	-0.25804
H	1.561131	1.469531	2.429016
O	3.596693	3.34852	-0.74975
O	3.612141	3.530178	0.443615
S	-3.90088	1.380816	-0.36512
O	-2.89364	0.56384	-1.16304
C	-3.12838	1.819199	1.20593
H	-3.82278	2.4419	1.775123
H	-2.94569	0.87653	1.726071
H	-2.19126	2.356709	1.035361
C	-3.89784	3.028707	-1.10232
H	-4.30049	2.9349	-2.11149
H	-4.53178	3.691864	-0.50906
H	-2.86927	3.397741	-1.13617
S	1.054915	-3.77434	-1.01926
O	-0.19628	-2.91457	-0.85136
C	2.047316	-2.96715	-2.28983
H	2.990029	-3.50597	-2.40707
H	1.476918	-3.00635	-3.21853
H	2.243076	-1.93479	-1.9933
C	2.104184	-3.39103	0.392808
H	1.624307	-3.81315	1.276803
H	3.090352	-3.83639	0.254146
H	2.20433	-2.30933	0.499422
S	-3.3436	-2.41656	1.080306
O	-2.38388	-1.28294	1.411655
C	-3.39696	-2.58547	-0.72081
H	-4.17243	-3.3109	-0.97768
H	-3.63506	-1.5969	-1.11972
H	-2.41925	-2.90415	-1.0902
C	-2.43313	-3.94135	1.415638
H	-2.26231	-3.99535	2.49137
H	-3.02506	-4.79888	1.087315
H	-1.48808	-3.88302	0.87097

3-2K-2DMSO

C	3.419686	-2.44741	1.974
C	2.232551	-2.79984	1.346599
C	2.106391	-2.72898	-0.05755
C	3.229396	-2.30073	-0.79227
C	4.413213	-1.95492	-0.15898
C	4.515095	-2.01648	1.22784
H	3.495874	-2.52216	3.054983
H	1.389986	-3.1442	1.933288
H	3.139037	-2.21529	-1.8692
H	5.257922	-1.61645	-0.75057
H	5.439577	-1.73873	1.72377
C	0.901653	-3.07771	-0.77115
C	-0.43312	-3.01346	-0.28718
C	-1.49478	-3.5603	-1.05754
C	-2.79433	-3.46067	-0.61026
H	-1.26311	-4.06045	-1.9921

C	-1.96733	-2.295	1.296331
C	-3.05092	-2.82237	0.607568
H	-3.60872	-3.87629	-1.19578
H	-2.12448	-1.76468	2.233737
H	-4.05721	-2.72298	0.995857
N	-0.70439	-2.35746	0.876726
K	0.956821	0.133851	1.037661
O	0.689607	-0.93848	-2.12165
O	1.44975	0.01135	-1.66449
C	0.17462	3.444318	1.213755
C	-0.95135	2.934963	0.285245
H	-0.09017	3.219861	2.253081
H	1.128416	2.954278	0.989579
H	0.331413	4.526662	1.130442
C	-0.52733	3.211353	-1.17626
C	-2.21598	3.764108	0.584802
H	-1.3675	3.008492	-1.85442
H	-0.21603	4.248073	-1.34985
H	0.298683	2.546125	-1.44681
H	-2.06712	4.837539	0.420321
H	-3.04051	3.434108	-0.0559
H	-2.51824	3.617497	1.626885
O	-1.18204	1.585843	0.471312
K	-1.71787	0.058229	-1.5596
H	1.024897	-3.43929	-1.7844
S	4.166759	1.621277	-0.2482
O	3.206475	1.480717	0.918734
C	3.284415	2.487399	-1.56341
H	3.997382	2.770996	-2.34144
H	2.541435	1.795023	-1.96451
H	2.791777	3.367688	-1.14422
C	5.262491	2.984664	0.214311
H	5.848498	2.654773	1.072846
H	5.926667	3.227778	-0.618
H	4.655207	3.848049	0.494537
S	-4.82886	1.090164	0.053695
O	-4.27662	0.119401	-0.98336
C	-6.38055	0.351138	0.61411
H	-6.78029	0.929772	1.44967
H	-7.0813	0.370209	-0.22134
H	-6.19197	-0.6821	0.914084
C	-3.87641	0.858599	1.561215
H	-2.82866	1.141834	1.314034
H	-4.2825	1.518091	2.332733
H	-3.94666	-0.18724	1.868906

3-2K-4DMSO

C	5.08204	-1.87245	1.848157
C	3.73139	-2.13237	1.68829
C	3.246189	-2.71769	0.495529
C	4.189504	-3.01021	-0.5199
C	5.537328	-2.75318	-0.34795
C	5.994879	-2.18856	0.843978
H	5.428788	-1.41541	2.769093
H	3.026985	-1.89482	2.47309
H	3.838593	-3.45457	-1.44691
H	6.238814	-2.99864	-1.13952
H	7.053443	-1.99415	0.985778
C	1.882856	-3.06593	0.250249
C	0.711802	-2.86024	1.047602
C	-0.50704	-3.44097	0.625508
C	-1.65844	-3.23576	1.361062
H	-0.52605	-4.02733	-0.28682
C	-0.35233	-1.92514	2.869636
C	-1.58762	-2.46039	2.518684
H	-2.61258	-3.63086	1.030409
H	-0.2608	-1.30149	3.75583
H	-2.47167	-2.24256	3.104447
N	0.763831	-2.09831	2.166723
K	1.369949	0.769422	0.840176
O	0.422637	-1.01418	-1.24476
O	0.655124	0.127836	-1.88191
C	-1.1348	2.894356	3.418643
C	-1.9115	2.147347	2.313817
H	-0.3716	2.227067	3.832919
H	-0.62476	3.769499	3.006404
H	-1.77858	3.232978	4.239149
C	-2.96206	3.107803	1.713162
C	-2.67189	0.985994	2.994683
H	-3.52366	2.588027	0.926643
H	-3.67697	3.483675	2.454614
H	-2.46898	3.96572	1.247691
H	-3.37431	1.336253	3.761722
H	-3.23418	0.399114	2.260254
H	-1.95828	0.310812	3.473774
O	-1.05805	1.688559	1.333489
K	-1.94202	-0.21866	-0.24654
H	1.694473	-3.55671	-0.69947

S	4.596486	1.850769	-0.39901
O	3.996876	1.302865	0.885745
C	6.305675	1.250779	-0.41959
H	6.77302	1.508717	-1.37297
H	6.834634	1.74593	0.395962
H	6.308292	0.171602	-0.25966
C	3.987361	0.795369	-1.73286
H	2.909996	0.956056	-1.81626
H	4.480863	1.078417	-2.66614
H	4.196402	-0.2482	-1.48245
S	-5.59479	-1.14254	0.321393
O	-4.15169	-1.44999	0.684111
C	-6.32545	-0.39529	1.797962
H	-7.3282	-0.02916	1.566839
H	-6.38122	-1.16924	2.564273
H	-5.68077	0.415694	2.143317
C	-5.58357	0.345181	-0.70319
H	-5.0101	0.110418	-1.60446
H	-6.61214	0.610078	-0.95903
H	-5.10548	1.16294	-0.15703
S	0.774747	3.886225	-1.2379
O	1.836096	2.883319	-0.82302
C	-0.76133	2.969347	-1.50763
H	-1.52302	3.668627	-1.86382
H	-0.55006	2.210928	-2.26204
H	-1.05215	2.508195	-0.54958
C	0.248133	4.674375	0.298865
H	1.103897	5.216202	0.703498
H	-0.57556	5.364721	0.101329
H	-0.06842	3.86862	0.967495
S	-2.57694	-1.10915	-3.85232
O	-3.18084	-0.45088	-2.61307
C	-1.22432	-0.0447	-4.38944
H	-0.7414	-0.50642	-5.25471
H	-1.66674	0.907767	-4.68652
H	-0.5113	0.087186	-3.56011
C	-1.55677	-2.47552	-3.25654
H	-2.23092	-3.2004	-2.79605
H	-1.05463	-2.93668	-4.11081
H	-0.82472	-2.0931	-2.52804

6-2K-2DMSO

C	3.892678	1.255567	-1.78039
C	2.791799	1.692937	-1.05399
C	2.699807	1.48986	0.34728
C	3.797231	0.824734	0.948934
C	4.892763	0.400284	0.217706
C	4.955314	0.602413	-1.16256
H	3.927918	1.4505	-2.84941
H	1.994241	2.231782	-1.55077
H	3.775882	0.659987	2.0231
H	5.715905	-0.09063	0.731675
H	5.821258	0.28372	-1.73461
C	1.617052	1.946251	1.18061
C	0.394274	2.505877	0.813388
C	-0.40412	3.207428	1.793352
C	-1.55619	3.849219	1.428076
H	-0.03044	3.274835	2.81069
C	-1.22956	3.045642	-0.78755
C	-2.0062	3.784682	0.088073
H	-2.116	4.419371	2.16598
H	-1.54488	2.94705	-1.82731
H	-2.89806	4.299671	-0.2471
N	-0.10288	2.398238	-0.4735
K	0.550245	-0.35266	-0.92117
C	-1.0269	-3.67735	-0.03465
C	-1.39675	-2.55697	0.96058
H	-1.79718	-3.75068	-0.80926
H	-0.07399	-3.44516	-0.52042
H	-0.93845	-4.65937	0.447047
C	-0.28981	-2.47687	2.03266
C	-2.71974	-2.94105	1.654194
H	-0.52572	-1.6916	2.759444
H	-0.15411	-3.41687	2.58181
H	0.661609	-2.21135	1.560728
H	-2.66404	-3.89955	2.18289
H	-2.99794	-2.17185	2.38512
H	-3.52347	-3.00579	0.914388
O	-1.52213	-1.34594	0.308556
K	-2.51189	0.830715	1.207904
H	1.837162	1.963784	2.244722
O	1.754887	4.795533	-0.95323
O	2.209277	4.815082	0.165287
S	3.18431	-2.79279	0.172248
O	2.190046	-2.37814	-0.89749
C	2.796305	-4.51937	0.541213
H	3.546077	-4.92978	1.221258
H	1.814353	-4.52934	1.015757

H	2.75846	-5.08677	-0.39112
C	4.740819	-3.0802	-0.69397
H	5.07088	-2.10754	-1.05916
H	5.477972	-3.49424	-0.00227
H	4.561617	-3.7581	-1.53112
S	-5.07712	-0.98256	-0.68511
O	-4.79444	0.323026	0.046276
C	-6.30902	-0.57097	-1.94136
H	-6.47041	-1.43465	-2.59017
H	-7.23601	-0.31282	-1.42809
H	-5.95493	0.286674	-2.51755
C	-3.67976	-1.27593	-1.77837
H	-2.79888	-1.39524	-1.10971
H	-3.86188	-2.18794	-2.35311
H	-3.5615	-0.41699	-2.44586

6-2K-4DMSO

C	4.033418	-2.10181	0.093702
C	2.767212	-2.41736	0.572855
C	1.754248	-2.91301	-0.28128
C	2.117933	-3.09951	-1.63316
C	3.380753	-2.78203	-2.10714
C	4.353309	-2.26712	-1.25151
H	4.783831	-1.72914	0.786593
H	2.541422	-2.2923	1.625165
H	1.372134	-3.49299	-2.31963
H	3.608871	-2.93275	-3.1589
H	5.343836	-2.01988	-1.62111
C	0.422981	-3.27043	0.165949
C	-0.27318	-2.78444	1.270805
C	-1.49032	-3.42619	1.714278
C	-2.17967	-2.95705	2.79679
H	-1.82324	-4.31695	1.191141
C	-0.53401	-1.25897	3.032336
C	-1.70249	-1.82788	3.503719
H	-3.08331	-3.46463	3.125223
H	-0.11419	-0.39364	3.548303
H	-2.19905	-1.44759	4.38792
N	0.152075	-1.66041	1.955819
K	1.489799	0.277003	0.342219
C	0.15328	2.48739	-2.76512
C	-0.62089	1.319181	-2.12475
H	-0.33713	3.437103	-2.52395
H	1.173929	2.510757	-2.37088
H	0.209342	2.402626	-3.85687
C	0.098308	0.009233	-2.52859
C	-2.04365	1.287803	-2.72594
H	-0.30902	-0.84373	-1.97119
H	0.002351	-0.20467	-3.59972
H	1.171349	0.065675	-2.32018
H	-2.03235	1.161958	-3.81569
H	-2.62933	0.468818	-2.2974
H	-2.56838	2.222736	-2.50889
O	-0.67442	1.460619	-0.74087
K	-2.31869	-0.29251	0.521473
H	-0.04075	-4.09186	-0.37308
O	1.661705	-3.79647	3.519355
O	1.516047	-4.70387	2.735781
S	4.698625	1.477185	-0.6908
O	3.307556	1.364868	-1.28143
C	5.796177	1.841628	-2.07881
H	6.797155	2.075911	-1.70977
H	5.828257	0.94932	-2.70496
H	5.381418	2.67644	-2.6479
C	4.767458	3.092604	0.113081
H	4.043603	3.04755	0.931429
H	5.773752	3.2734	0.498032
H	4.479596	3.864283	-0.60552
S	-3.85085	-2.61193	-2.2901
O	-3.80149	-1.49625	-1.26342
C	-2.86508	-2.07936	-3.70964
H	-2.83296	-2.89054	-4.44052
H	-3.35569	-1.20686	-4.14154
H	-1.86067	-1.80487	-3.3844
C	-2.71202	-3.88469	-1.70905
H	-3.15365	-4.33697	-0.82095
H	-2.58065	-4.64281	-2.48449
H	-1.75605	-3.42402	-1.44394
S	1.113423	3.467159	2.114353
O	2.130867	2.336357	1.982581
C	0.82043	4.066451	0.436249
H	0.101034	4.8884	0.471151
H	1.774487	4.429354	0.051818
H	0.434968	3.234274	-0.16975
C	-0.50172	2.680195	2.333536
H	-0.42872	2.024042	3.201627
H	-1.25795	3.444001	2.521367
H	-0.7556	2.124066	1.428379

S	-4.09099	2.796767	0.1542
O	-3.67007	1.894536	1.304879
C	-5.13193	4.077441	0.891551
H	-5.34794	4.846477	0.14654
H	-6.05879	3.606231	1.220259
H	-4.61031	4.504282	1.751064
C	-2.66484	3.819374	-0.24121
H	-1.85866	3.100023	-0.47586
H	-2.89667	4.442122	-1.10875
H	-2.40482	4.439912	0.619591

3-3K-3DMSO

C	2.987739	-0.67817	1.777651
C	1.820226	-1.41305	1.915628
C	1.513253	-2.08813	3.115878
C	2.483415	-2.07372	4.14079
C	3.658658	-1.35409	3.999434
C	3.905437	-0.63652	2.829026
H	3.154836	-0.12422	0.850549
H	1.124101	-1.50304	1.091864
H	2.284419	-2.61196	5.063507
H	4.382025	-1.3409	4.809181
H	4.814753	-0.05049	2.731379
C	0.27635	-2.77667	3.332037
C	-0.97969	-2.56209	2.674495
C	-2.02996	-3.48383	2.88781
C	-3.2497	-3.28575	2.270282
H	-1.86042	-4.34662	3.523721
C	-2.34737	-1.28087	1.323443
C	-3.42109	-2.16136	1.467572
H	-4.05778	-3.99742	2.413562
H	-2.4459	-0.38144	0.703312
H	-4.34759	-1.9657	0.939368
N	-1.16043	-1.4692	1.894627
K	0.151843	0.704771	0.016103
O	-0.18418	-1.84618	-1.18402
O	-0.15208	-1.74493	-2.51777
C	-2.42984	3.384523	-0.3293
C	-3.16119	2.091645	-0.75394
H	-1.89258	3.185786	0.602377
H	-1.68806	3.645252	-1.09312
H	-3.10097	4.24221	-0.1887
C	-3.81844	2.340456	-2.13127
C	-4.27807	1.797923	0.274576
H	-4.36448	1.442597	-2.4442
H	-4.51745	3.186834	-2.13765
H	-3.03583	2.530076	-2.87235
H	-5.0635	2.564871	0.311829
H	-4.72345	0.832468	0.012811
H	-3.84249	1.69801	1.274096
O	-2.29223	1.039557	-0.81526
K	-2.65515	-1.22779	-1.94272
C	3.672999	2.711322	-0.10948
C	3.165169	1.988658	-1.37524
H	2.915325	2.626901	0.674308
H	4.583041	2.223066	0.255054
H	3.902947	3.771884	-0.27897
C	4.258077	2.078051	-2.46203
C	1.928379	2.750953	-1.90789
H	3.929251	1.529022	-3.35193
H	4.484216	3.108793	-2.75943
H	5.186107	1.625995	-2.10021
H	2.169711	3.765365	-2.24943
H	1.494334	2.200203	-2.74876
H	1.165015	2.846774	-1.12993
O	2.858271	0.678233	-1.08524
K	2.368443	-1.68638	-1.89022
H	0.266833	-3.51468	4.130689
S	6.078035	-2.05926	-1.64054
O	4.774699	-2.78052	-1.94708
C	6.393544	-0.9766	-3.05394
H	7.276199	-0.36358	-2.85857
H	6.573063	-1.61761	-3.91804
H	5.523169	-0.34216	-3.22693
C	5.689416	-0.76908	-0.44043
H	5.507157	-1.26488	0.514236
H	6.549339	-0.10084	-0.34526
H	4.782743	-0.21668	-0.74447
S	-6.73694	-1.3534	-1.37753
O	-5.23597	-1.54229	-1.46433
C	-7.12094	-1.07954	0.369229
H	-8.17864	-0.82709	0.471765
H	-6.91315	-2.00973	0.899495
H	-6.48794	-0.28109	0.76085
C	-7.10074	0.325457	-1.94336
H	-6.84674	0.374312	-3.00292
H	-8.16738	0.521235	-1.81193
H	-6.49556	1.045035	-1.39047

S	0.849466	4.092742	2.168003
O	0.50747	2.705105	1.676979
C	0.797481	5.185811	0.727366
H	0.896241	6.223437	1.054178
H	1.636866	4.913282	0.087583
H	-0.13993	5.02687	0.19084
C	-0.64634	4.722105	2.972574
H	-0.82435	4.097607	3.848783
H	-0.49004	5.758582	3.280167
H	-1.48944	4.63868	2.284562

6-3K-DMSO

O	2.156729	0.573499	1.573686
O	0.718619	-0.72264	-1.9903
K	-1.27143	-2.37068	-1.65884
K	2.183691	-1.69142	0.27484
K	0.572396	1.280134	-0.2848
C	2.412944	0.844735	2.902134
C	1.377292	-0.77424	-3.20449
C	1.790876	-2.22279	-3.54879
H	0.90502	-2.86822	-3.61663
H	2.323546	-2.29994	-4.50294
H	2.444345	-2.62357	-2.76684
C	0.473201	-0.25285	-4.34024
H	0.185152	0.7833	-4.13169
H	0.95691	-0.28308	-5.32305
H	-0.44138	-0.85587	-4.3951
C	2.660441	0.080899	-3.17754
H	2.401489	1.121342	-2.94464
H	3.336504	-0.29553	-2.40484
H	3.19329	0.075629	-4.1361
C	1.941286	2.271912	3.254502
H	2.143283	2.55129	4.295094
H	2.446767	2.991215	2.598924
H	0.86232	2.353689	3.08451
C	1.680368	-0.15184	3.821541
H	2.023399	-1.17115	3.611258
H	1.852562	0.049526	4.885277
H	0.603995	-0.12719	3.635245
C	3.923331	0.748482	3.2051
H	4.289887	-0.2422	2.915153
H	4.480599	1.494853	2.629912
H	4.153657	0.901886	4.26552
C	-0.11698	-4.5818	0.810223
C	-1.26279	-5.3595	0.846678
C	-2.45931	-4.69237	1.15896
C	-2.46143	-3.33537	1.366846
C	-1.24868	-2.57505	1.251439
N	-0.07669	-3.25841	0.980531
H	-3.38543	-5.25186	1.26371
H	0.845691	-5.05729	0.615503
H	-1.22039	-6.43037	0.690008
H	-3.37143	-2.83401	1.667206
C	-1.16812	-1.17793	1.387288
H	-0.1792	-0.78798	1.61384
C	-2.24651	-0.23148	1.476546
C	-3.52101	-0.38638	0.869156
C	-2.03962	1.002208	2.150499
C	-4.49453	0.599763	0.938197
H	-3.73382	-1.27279	0.283565
C	-3.02498	1.974775	2.227588
H	-1.09196	1.155411	2.66018
C	-4.26727	1.795561	1.617265
H	-5.44771	0.430862	0.44415
H	-2.83368	2.879746	2.800085
H	-5.04146	2.551935	1.692406
O	-2.78056	-1.97007	4.23164
O	-1.64327	-1.57178	4.302969
S	-0.82813	4.894181	-0.2475
O	-0.38233	3.646233	-0.99176
C	-2.62976	4.801911	-0.13433
H	-2.9941	5.618239	0.493608
H	-3.0232	4.918298	-1.14514
H	-2.92773	3.834183	0.276894
C	-0.47656	4.594328	1.498276
H	0.60616	4.540068	1.617712
H	-0.87344	5.4147	2.100405
H	-0.9266	3.645174	1.793186
S	-3.65383	0.372243	-2.66624
O	-3.3635	-1.1169	-2.5528
C	-2.24393	1.229584	-1.93792
H	-2.21004	2.269141	-2.26668
H	-2.38044	1.190797	-0.85654
H	-1.33055	0.697902	-2.23249
C	-3.30848	0.809847	-4.3872
H	-4.01785	0.263728	-5.01005
H	-3.44785	1.88477	-4.52222
H	-2.28744	0.519096	-4.6395

S	5.4927	0.032175	-0.10811
O	4.654534	-1.17421	-0.51022
C	6.531981	0.381312	-1.54702
H	7.238555	-0.44243	-1.65523
H	5.89624	0.441686	-2.433
C	4.430018	1.483928	-0.21073
H	3.57781	1.280643	0.472686
H	4.998643	2.353154	0.130402
H	4.091864	1.623146	-1.23959
H	7.072116	1.317734	-1.39068
